# Measuring and forecasting progress towards the education-related SDG targets

**DOI:** 10.1038/s41586-020-2198-8

**Published:** 2020-04-15

**Authors:** Joseph Friedman, Hunter York, Nicholas Graetz, Lauren Woyczynski, Joanna Whisnant, Simon I. Hay, Emmanuela Gakidou

**Affiliations:** 1 Institute for Health Metrics and Evaluation, University of Washington, Seattle, WA USA; 2 Center for Social Medicine and Humanities, University of California Los Angeles, Los Angeles, CA USA; 3 Population Studies Center, University of Pennsylvania, Philadelphia, PA USA; 4 Department of Health Metrics Sciences, School of Medicine, University of Washington, Seattle, WA USA

**Keywords:** Education, Society

## Abstract

Education is a key dimension of well-being and a crucial indicator of development^1–4^. The Sustainable Development Goals (SDGs) prioritize progress in education, with a new focus on inequality^5–7^. Here we model the within-country distribution of years of schooling, and use this model to explore educational inequality since 1970 and to forecast progress towards the education-related 2030 SDG targets. We show that although the world is largely on track to achieve near-universal primary education by 2030, substantial challenges remain in the completion rates for secondary and tertiary education. Globally, the gender gap in schooling had nearly closed by 2018 but gender disparities remained acute in parts of sub-Saharan Africa, and North Africa and the Middle East. It is predicted that, by 2030, females will have achieved significantly higher educational attainment than males in 18 countries. Inequality in education reached a peak globally in 2017 and is projected to decrease steadily up to 2030. The distributions and inequality metrics presented here represent a framework that can be used to track the progress of each country towards the SDG targets and the level of inequality over time. Reducing educational inequality is one way to promote a fairer distribution of human capital and the development of more equitable human societies.

## Abstract (web summary)

Great progress toward the education-related SDG targets has been made; however, global estimates of within-country distributions of education reveal gender disparities and high levels of total inequality in many parts of the world.

## Main

The value of education is well-recognized, both as a primary human right and as a key driver of progress in economic development, health, fertility, politics, social empowerment, and human capital^3–13^. The international community recognized educational attainment as a key development priority in the Millennium Development Goals (MDGs), which became a key focus for a large variety of global actors. The education-related MDG targets focused largely on expanding primary education up to 2015^14^, and great progress in this regard was seen as a result. In the SDGs—the follow-up to the MDGs with a target year of 2030—education was again highly prioritized, with a wider scope that emphasized reducing inequalities.

## Increases in global schooling rates

**Fig. 1:**
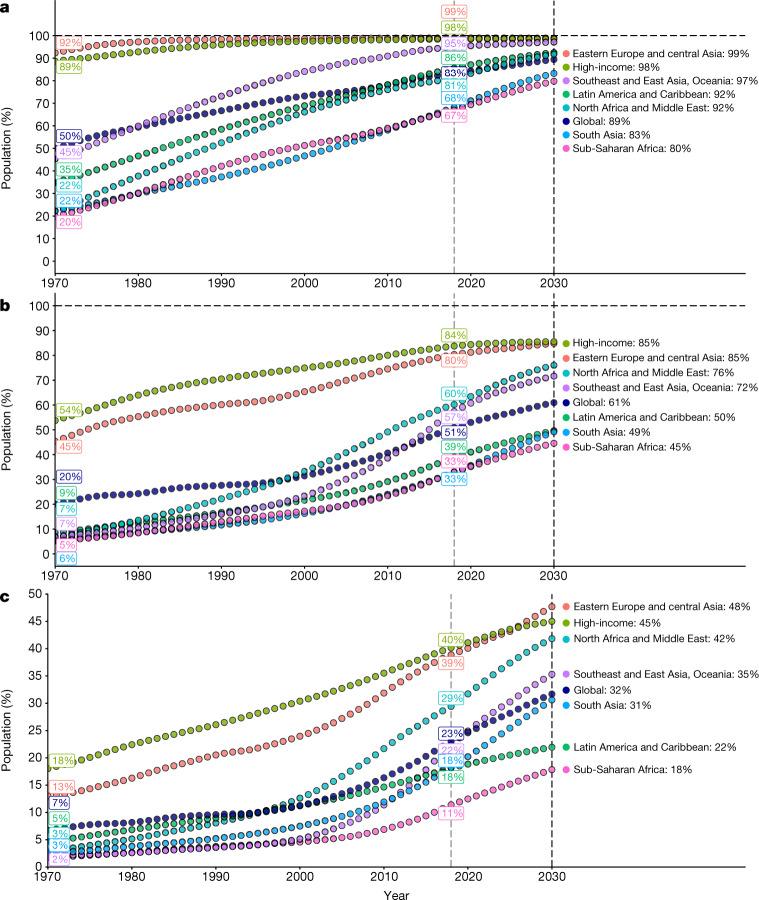
Regional attainment of primary, secondary, and tertiary schooling from 1970 to 2030. **a**–**c**, Attainment rates of 6+ (**a**), 12+ (**b**), and 15+ (**c**) years of schooling are shown. All trends reflect 25–29-year-old individuals separated by major world region. The vertical dashed lines indicate 2018, when the forecasts begin, and 2030, the target year for the SDGs. Source data

**Extended Data Fig. 1:**
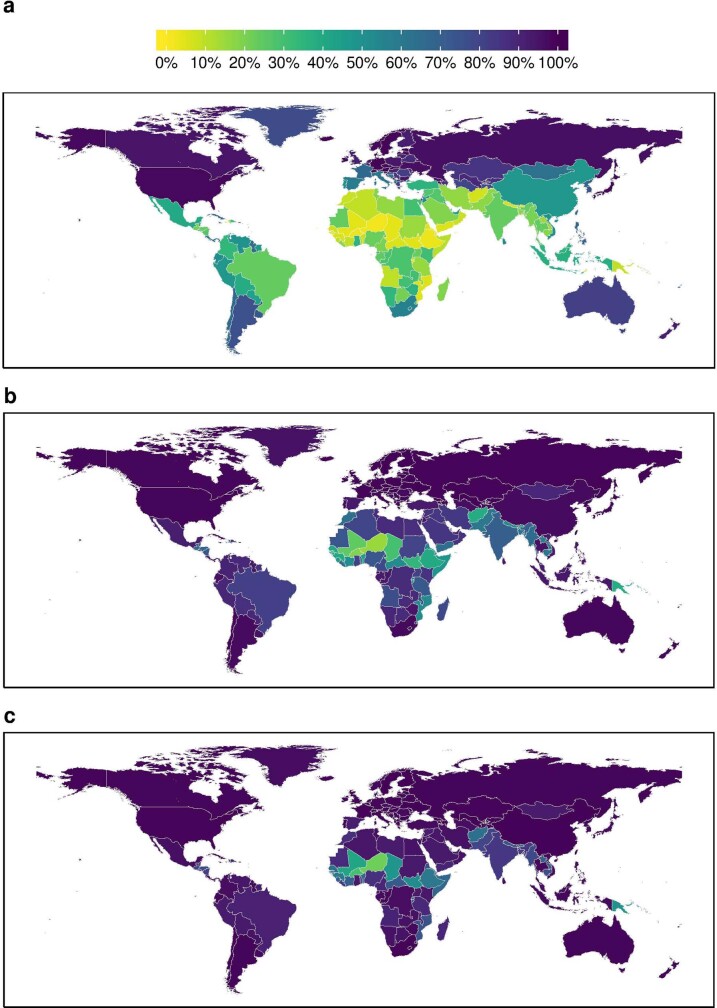
Completion of 6 or more years of schooling. **a**–**c**, The percentage of the population aged 25–29 completing at least 6 years of schooling is shown by country, for 1970 (**a**), 2018 (**b**), and 2030 (**c**). Maps were produced using R v.3.5.0.

SDG target 4.1 calls for universal primary schooling. Progress towards this goal has been, and is projected to continue to be, substantial (Fig. 1). Globally, the proportion of 25–29-year olds with at least 6 years of schooling rose from 50.1% (95% uncertainty interval: 49.3–51.0%) in 1970 to 83.2% (82.1–84.0%) in 2018 and is projected to reach 89.4% (87.4–91.0%) by 2030. Even as far back as 1970, countries in high-income regions and in eastern Europe and central Asia had on average already achieved near universal primary attainment. In the remaining regions, rates of primary attainment have risen substantially. Although this progress is to be celebrated, important gaps remain in a subset of nations that are not projected to achieve near universal levels of primary attainment by 2030, largely due to gaps in schooling among women (see Extended Data Fig. 1).

SDG target 4.1 also calls for universal secondary schooling. However, secondary attainment estimates reveal a much more heterogeneous picture. In 1970, countries generally fell into one of two categories; nearly 50% of the global population aged 25–29 residing in highly educated regions had already attained 12 years of schooling, whereas the rest of the world saw rates at or below 10%. Although global attainment of at least 12 years of schooling has risen steadily since 1970, no major world region has achieved near universal levels. All regions have seen progress, yet the inter-regional disparities remain massive in 2018 and are projected to decrease only slightly in the coming years.

SDG target 4.3 addresses tertiary education, calling for ‘equal access’ for all individuals. Tertiary education exhibited a substantial scale-up between 1970 and 2018 that is projected to continue in the coming decade, although global completion rates remain low. Similar to the trend in secondary education, the high-income and eastern European and central Asian regions exhibit substantially higher rates throughout the time period shown, and are projected to achieve about half of their population completing tertiary education by 2030. The remaining regions have also seen progress, with much of the growth seen after 2000. The increase is particularly notable in North Africa and the Middle East as well as in Southeast Asia, East Asia, and Oceania.

In summary, regional disparities in tertiary education completion are increasing over time and are projected to continue to do so, whereas secondary gaps are expected to decrease only slightly. The success of narrowing the global gap for primary education has not been extended to higher levels of education, which raises concerns about gaps in opportunities amplifying across regions in the coming decade.

## Progress towards gender equity

Gender equity has been a central focus of the SDG targets. SDG target 5 calls for gender equity broadly, and target 4.5 calls for the elimination of all gender disparities in education. We find that great strides have been made in reversing educational disparities for women globally, and in all regions of the world.

**Fig. 2:**
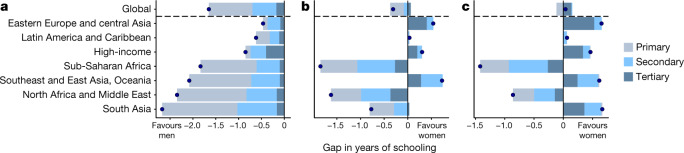
Regional gender gaps in primary, secondary, tertiary, and total schooling. **a**–**c**, The gender gap is shown for 1970 (**a**), 2018 (**b**), and 2030 (**c**). The total gap in years of schooling is represented by a dot, for individuals aged 25–29, separated by each regional group. The grey, light blue, and dark blue bars represent the contributions of primary, secondary, and tertiary schooling, respectively, to the total gender gap. Source data

To benchmark the progress of each country towards gender parity in education, we calculate the absolute gap in the mean years of schooling, and assess the contribution of primary, secondary, and tertiary schooling to these gaps (Fig. 2). In 1970, men aged 25–29 years had completed on average 1.7 (1.6–1.8) additional years of education compared with women of the same age. By 2018, this gap had nearly closed, falling to only 0.3 (−0.2–0.8) years, and is projected to reverse by 2030. Previous modelling studies of global gender differences in educational attainment that have focused on all adults 25 and older show progress, but note that women are not yet close to catching up to men^15^. By focusing only on young women and men, we show that among the most recently educated members of societies, women had in fact nearly closed the gender gap in 2018. Young men had statistically significantly higher levels of attainment compared with women, at the 95% confidence level, in 142 countries in 1970, 27 countries in 2018, and only 4 countries by 2030. For 2030, the countries in which women’s education is predicted to still lag behind that of men are predominantly in sub-Saharan Africa, Southeast Asia, East Asia, and Oceania. In addition, by 2030, women are expected to achieve statistically significantly higher mean years of schooling than men in 18 countries—a tremendous reversal of the global landscape that was observed in 1970.

In absolute terms, the largest component of this reduction has been observed in primary education. In 1970, men aged 25–29 completed 0.9 (0.9–1.0) additional years of primary schooling compared with women, which fell to only 0.3 (0.2–0.4) years in 2018. This reflects progress in nearly every region; all had primary education gaps favouring men in 1970. By 2018, these gaps had shrunk by considerable margins in every region, and many disappeared entirely. Nevertheless, a small number of countries are forecast to have persistent gaps in attainment of at least 6 years of schooling, largely in North Africa and the Middle East, as well as sub-Saharan Africa (Supplementary Fig. 14).

Secondary and tertiary education both show a more heterogeneous pattern, in which women are overtaking men in most regions of the world, whereas large-magnitude disparities seen in sub-Saharan Africa and North Africa and the Middle East are projected to persist. Our estimates indicate that in 2012, women aged 25–29 overtook men in the global average of tertiary attainment, and they are forecast to do so for secondary attainment in 2026. Unlike primary attainment, which has largely converged globally in a place of gender parity, women have overtaken men by substantial margins in many nations in Latin America, Asia, and Europe. This phenomenon has been reported for many nations in the Organisation for Economic Co-operation and Development (OECD)^16,17^ and elsewhere^18–20^, in which boys increasingly fall behind girls in schooling as nations develop. Our results indicate the commonality of this trend for many regions of the world, and show how these advances have contributed to closing the overall gender gap. Notably, our results indicate that these gaps are projected to grow with time.

## Assessment of inequalities in education

Although gender equity is of crucial importance, it only captures one dimension of inequality in education. Beyond gender, SDG target 4.5 calls for broad social equity in educational attainment, across lines of ethnicity, race, socio-economic status, ability, and other identities^21^. The particular social groupings that are relevant vary across countries, but insight between countries can be gleaned by assessing the total inequality.

To facilitate benchmarking between nations and a global assessment of trends in educational inequality, we use a metric of the total within-country inequality in education, the average interpersonal difference (AID), which represents the average difference between any two individuals in a population. Results and discussion using alternative metrics of inequality, including relative measures such as the Gini coefficient, are presented in the Supplementary Information.

**Fig. 3:**
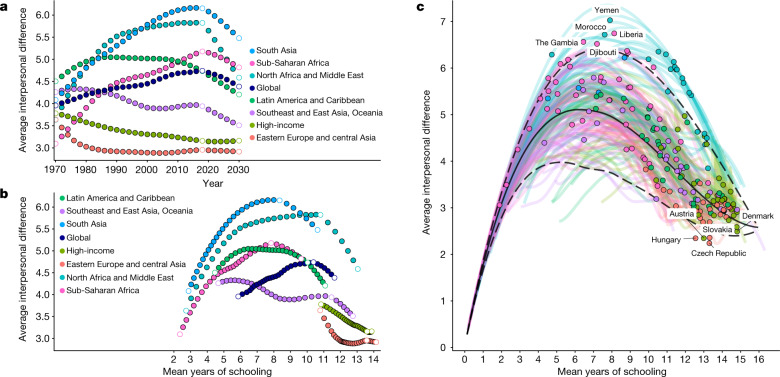
Trajectories in educational inequality. **a**, Trends in educational inequality are shown over time, with labels indicating the rank of inequality levels in 2030. **b**, Trends in educational inequality are shown with respect to mean years of schooling, with labels indicating the rank of inequality levels in 1970. Results are shown globally and regionally for every second year from 1970 to 2030, for individuals aged 25–29. The white dots mark 1970, the beginning of the estimates, 2018, the beginning of forecasts, and 2030, the SDG target year. **c**, National trends in the AID and mean years of schooling are shown from 1970 to 2030, with the value for 2018 shown as a bold point. The five highest and lowest values in 2018 are labelled. The solid line shows the median level of inequality for a given degree of mean years of schooling, across all years of data from 1970 to 2030, and the dashed lines show the smoothed ninety-fifth and fifth percentiles. Quantiles were calculated over modelled estimates from *n* = 195 countries. Source data

Globally, inequality rose steadily before peaking in 2017 with a 4.6-year (4.5–4.7) average within-country difference between any two given individuals (Fig. 3a). Subsequently, inequality has been decreasing and is projected to continue to do so up to 2030. Looking at the arc of inequality in education over time across regions and countries, a consistent Kuznets curve can be observed in almost every setting. A Kuznets curve describes a development trend in which progress is associated with first increased and then decreased inequality, creating an inverse-U-shaped curve^22^.

We observe substantial variation in the maximum level of inequality reached during each period, which in some cases reflect threefold differences in the degree of equality for a given average level of schooling. In this way, these curves provide a valuable tool for comparing the level of inequality of each country compared with their neighbours, relative to their overall level of progress.

Latin America and the Caribbean had the highest levels of inequality in 1970, with an AID of 4.5 years (4.4–4.6) (Fig. 3a). Over time, however, Latin America and the Caribbean has had an only intermediate-height Kuznets curve, despite substantial progress in the mean years of educational attainment (Fig. 3b). Latin America and the Caribbbean stands out as having less inequality in education at each point in the development arc compared with regions such as South Asia or North Africa and the Middle East, as shown by a lower overall Kuznets curve. This result highlights the need to assess inequality for each region with respect to its level of development by looking across decades to understand variation in the arc of educational expansion.

Between 1970 and 2018, sub-Saharan Africa and South Asia saw great advances in education, and also large increases in inequality. South Asia had the highest level of educational inequality globally in 2018, with an AID value of 6.0 (5.7–6.3). Its Kuznets curve is largely similar to that of North Africa and the Middle East. If sub-Saharan Africa continues to develop at its current trajectory, we expect its trend to look similar to that of Latin America and the Caribbean, which is approximately 30 years further along the development arc. Taking a more granular look, substantial variation can be seen between nations in sub-Saharan Africa (Fig. 3c). In 2018, several countries in western sub-Saharan Africa displayed the highest inequality values in the world, well above the ninetieth percentile mark for their level of mean attainment. Nevertheless, several nations in southern Africa are below the tenth percentile of inequality values for their mean attainment.

The region of Southeast and East Asia and Oceania is noteworthy for having the flattest Kuznets curve, and therefore the least unequal trajectory of development among low- and middle-income countries. Eastern Europe and central Asia underwent rapid gains in education from 1970 to 1995, achieving mean values similar to high-income countries by 2018, with lower overall inequality.

## Centring equality in global progress

Educational inequalities exist in many different forms and need to be addressed in order for societies to maximize well-being and the potential for education to facilitate economic development. Gender gaps are projected to persist for girls in much of the developing world and widen for boys in a subset of developed countries^16,23^. Disparities can also be found along dimensions of wealth, ethnicity, race, ability, and other social groupings^20,24^. Previous work has shown substantial inequalities in education between urban and rural areas^5,25,26^, and along lines of wealth^20^. These inequalities are easy to miss when drawing on national average measures of attainment.

The distributions and inequality metrics presented here provide a framework that can be used to track the progress of each country towards the SDG targets and levels of inequality over time. Once detected, inequalities can be reduced with the implementation of specific policies. For example, eliminating school fees, improving local access to schools, increasing the number of years of compulsory schooling, and providing food, stipends, and other resources for children at school are known to increase participation among the most economically disadvantaged children, and the creation of special governmental bodies can reduce gaps for children of minority ethnic groups^25,27,28^. It is therefore essential to examine progress in average levels of attainment with an understanding of the full within-country distribution and inequality. Gains in education are linked to improvements in numerous other sectors of society^3,4,13^. Ensuring equality in education will translate into positive effects in the equality of human productivity, health, and well-being.

## Methods

No statistical methods were used to predetermine sample size. The experiments were not randomized, and investigators were not blinded to allocation during experiments and outcome assessment.

### Overview

Our study follows the Guidelines for Accurate and Transparent Health Estimates Reporting (GATHER)^29^. We use a multi-stage model to estimate the average years of schooling, and the single-year distribution of educational attainment, for 1970 to 2018, and create projections to 2030. These models draw on a database of 3,180 nationally representative censuses and surveys. Estimates are created for the 195 counties and territories examined in the Global Burden of Disease 2017 study^30^. In the first stage, we model mean years of schooling and the proportion of the population without any formal schooling from 1970 to 2018. This is performed using a cohort extrapolation model and a subsequent age period model with Gaussian process regression to synthesis all data and create final estimates with uncertainty. The second stage entails an ensemble *K*-nearest neighbours algorithm to estimate the distribution of education from 1970 to 2018, drawing on previously estimated quantities. Finally, trends in these distributions are projected to 2030 using a rate of change approach, and mean years of schooling values for 2019–2030 are calculated from the resulting distributions. All analyses are run using 1,000 draws to propagate model and data uncertainty through to subsequent steps. All estimation steps are validated, and all hyper-parameters are optimized, using out of sample predictive validity.

### Data sources

We compiled a database of 3,180 nationally representative surveys and censuses describing the distribution of years of schooling by age and sex. Data sources providing single years of schooling are used directly, while those providing larger bins of educational attainment, for example ‘some primary attainment’ are probabilistically split into single-year proportions using a previously published crosswalk model^31^. Data are top-coded to 18 years, as it is a common choice among providers of single-year education data^32^, and it is reasonable to assume that the importance of education for health or social capital diminishes greatly after the completion of 18 years, which represents 2 to 3 years of post-university education in most educational systems.

### Data adjustment model

Data are adjusted for systematic biases between data providers in a regional and location-specific fashion. Gold-standard data are identified using expert knowledge of the high-volume data providers that have robust processes in place to ensure data quality. In almost all cases, census data obtained from the IPUMS data repository are considered as the gold standard, or Demographic Health Survey data where IPUMS are not available. Supplementary Table 3 lists the location-specific gold-standard data providers. Regional effects are applied to all data to adjust them to the gold standard available in that region. Subsequently, in countries that had gold-standard data available, country-specific effects are used to adjust for within-county biases between data sources. This has the benefit of being able to correct for biases in all countries, even when gold-standard data are not available in that country, using regional effects. Country-specific effects ensure consistent time trends with minimal discontinuities.

We use a mixed-effects regression model with random effects for data provider and nested random effects for data provider within country. This model is run separately for each region, and is formulated as follows:

$${\rm{l}}{\rm{o}}{\rm{g}}{\rm{i}}{\rm{t}}({P}_{Q,A,S,Y,L})={\beta }_{0}+{\beta }_{1}\times {\rm{a}}{\rm{g}}{\rm{e}}+{\beta }_{2}\times {\rm{s}}{\rm{e}}{\rm{x}}+{\beta }_{3}\times {\rm{l}}{\rm{o}}{\rm{c}}{\rm{a}}{\rm{t}}{\rm{i}}{\rm{o}}{\rm{n}}+{\beta }_{4}\,\times {\rm{y}}{\rm{e}}{\rm{a}}{\rm{r}}+{u}_{{\rm{d}}{\rm{a}}{\rm{t}}{\rm{a}}{\rm{p}}{\rm{r}}{\rm{o}}{\rm{v}}{\rm{i}}{\rm{d}}{\rm{e}}{\rm{r}}}+{u}_{{\rm{l}}{\rm{o}}{\rm{c}}{\rm{a}}{\rm{t}}{\rm{i}}{\rm{o}}{\rm{n}}:{\rm{d}}{\rm{a}}{\rm{t}}{\rm{a}}{\rm{p}}{\rm{r}}{\rm{o}}{\rm{v}}{\rm{i}}{\rm{d}}{\rm{e}}{\rm{r}}}$$

in which *P*_*Q,A,S,Y,L*_ is the quantity of interest, either proportion of the population with no education or $\frac{{\rm{mean}}\,{\rm{years}}\,{\rm{of}}\,{\rm{educational}}\,{\rm{attainment}}}{18}$ for a given age, sex, year, and location. *u*_data provider_ is a region-specific random effect that captures the average bias between data providers across all countries within that region, and *u*_location:data provider_ is a nested location-specific random effect that captures the additional bias between a location-specific gold standard (where applicable) and the other sources present in that location.

To calculate source adjustments for each data provider, the *u*_data provider_ value for each data provider is compared with the regional gold standard, and the difference is applied to all surveys. Subsequently, in locations that have gold-standard data present, *u*_location:data provider_ effects are applied in the same fashion.

### Cohort extrapolation

We use an age-cohort modelling process to project cohorts through time, leveraging the stability of cohort-specific educational attainment after age 25. To model the changes by age within cohorts, we use data from all available cohorts with multiple observations at or after age 25. For each quantity being modelled, we calculated ${y}_{Q,L,S,C,{A}_{x}}$, which is the logit difference of the *P*_*Q,A,S,Y,L*_′ (the adjusted input data) at time *x* and at time *y*, for all possible combinations of repeat cohort observations. We restrict repeat cohort observations to those that are less than or equal to 10 years apart and to those where both observations occur after 1990 to avoid the attribution of differences in measurements to mortality as opposed to advances in survey and census design. In addition, we normalize all repeat cohort observation pairings so that the average change at 65 years of age is 0 to account for systematic bias between survey iterations (such as improvements in sampling). This is similar to other previously described approaches^33^, in which only excess mortality beyond the age of 65 is considered. This calculation is shown below:

$${y}_{Q,L,S,C,{A}_{x}}={\rm{logit}}({P}_{L,S,C,A,{{\rm{Src}}}_{x}}{}^{{\prime} })-{\rm{logit}}({P}_{L,S,C,A,{{\rm{Src}}}_{y}}{}^{{\prime} })-{{\rm{bias}}}_{L,P,S,C,{\rm{Src}}}$$

in which *Q* is the quantity being modelled, *L* is location, *S* is sex, *C* is cohort, *A* is age, Src is data provider, and bias_*L,P,S,C*,Src_ is the average change for cohorts as they age from 60 to 70 between the two surveys. This is the age period for which we expect the educational attainment of a cohort to be least prone to changes due to migration and mortality, and any changes observed during this period are therefore used as a measure of inherent bias between multiple waves of a survey or census.

These logit differences were examined with respect to several predictor variables. We then modelled the logit difference using a number of linear mixed-effects models, which were evaluated using out-of-sample predictive validity (see Supplementary Information). The best performing model specification is displayed here:

$${y}_{Q,L,S,C,{A}_{x}}=I+{u}_{{\rm{location}}:{\rm{super}}{\rm{region}}}$$

in which *I* is a natural spline with a knot at age 70 intended to capture the potential nonlinearity in the rate of change of differential mortality by education over age. *u*_location:super region_ are random intercepts on location, nested within super-regional random intercepts.

### Age-period model

Age-period models were fit on all values of ${P}_{Q,A,S,Y,L}{\prime\prime}$, which reflect the adjusted input data after cohort extrapolation, to interpolate data for observed cohorts, and to extrapolate to all parts of the desired time series, producing ${P}_{Q,S,Y,L}{}^{\prime\prime\prime }$, single-year estimates of attainment from 1970 to 2018. Several linear mixed-effects models were used and evaluated using out-of-sample predictive validity (see Supplementary Information). Separately for each sex, and region grouping used in the GBD study, the quantity of interest of the country–age–year-specific population,${P}_{Q,A,S,Y,L}{}^{\prime\prime\prime }$ was estimated:

$${\rm{l}}{\rm{o}}{\rm{g}}{\rm{i}}{\rm{t}}({P}_{Q,A,S,Y,L}{\prime\prime} )=\,{\beta }_{s,r}+{\delta }_{s,r}{\rm{y}}{\rm{e}}{\rm{a}}{\rm{r}}+{I}_{s,r}+\,{\alpha }_{c,a,s}$$

in which ${\beta }_{s,r}$ is a sex- and region-specific intercept; *δ*_*s,r*_ captures the linear secular trend for each sex and region; *I*_*s,r*_ is a natural spline on age to capture the nonlinear age pattern by sex and region, with knots at 45 and 65 years of age; and ${\alpha }_{c,a,s}$ is a country-sex-specific random intercept.

### Gaussian process regression

Gaussian process regression (GPR) was used to ensure final model results are consistent with input data and incorporate model and data uncertainty to produce uncertainty intervals. GPR has been used extensively as a data synthesis tool^34^. GPR uses a covariance function to smooth the residuals from the age-period model, taking into account the uncertainty in each data point. GPR also synthesizes both data and model uncertainty, in order to produce estimate uncertainty intervals. GPR assumes that the trend in the underlying data follows a Gaussian process, which is defined using a mean function *m*(∙) and a covariance function Cov(∙). Therefore, separately for each *Q* quantity being estimated, the location–sex–age–year-specific outcome measures are defined:

$${\rm{logit}}({y}_{Q,L,S,C,A})=\,{g}_{Q,L,S,A,Y}\,+\,{{\epsilon }}_{Q,L,S,A,Y}\,$$

Where the error term is normally distributed:

$${{\epsilon }}_{Q,L,S,A,Y}={\rm{normal}}(0,{\sigma }_{p}^{2})$$

The error variance, ${\sigma }_{p}^{2}$ is composed of the squared standard error of the observed data point, as well as the prediction errors from the age-cohort imputation process. The mean function of the model is defined as the age-period model predictions, as detailed above. The covariance function of the model is derived using a Matérn covariance function, consistent with prior applications of GPR:

$$M(y,y{\prime} )={\sigma }^{2}\frac{{2}^{1-\nu }}{\varGamma (\nu )}\,{\left(\frac{d(y,y{\prime} )\sqrt{2\nu }}{l}\right)}^{\nu }{K}_{{\rm{\nu }}}\left(\frac{d(y,y{\prime} )\sqrt{2\nu }}{l}\right)$$

where *d*(∙) is a distance function, *σ*^2^ is the marginal variance, *ν* is a smoothness hyper parameter defining the differentiability of the function, *l* is a link-scale parameter approximately equivalent to the number of years at which two points are no longer correlated, *Κ*_ν_ is the Bessel function, and *Γ*(∙) is the gamma function. Similar to previous applications of GPR, we approximate ${\sigma }_{p}^{2}$ as the super-region and sex-specific residual from the mean function, with *ν* set to 2 and *l* to 40, to reflect the inherent smoothness of educational attainment trends over time.

### Ensemble *K*-nearest neighbours distribution model

To create a full time-series of distributions of single-years of educational attainment to 2018, we used a *K*-nearest neighbours algorithm to reconstruct an ensemble distribution for each location–age–sex–year (LASY) combination. To pick *K* candidate distributions for each LASY combination, we used two modelled entities produced by the above methods, mean educational attainment and proportion of the LASY population with 0 years of schooling, to find the most similar distributions in our database of 3,180 surveys and censuses. The metric used to find the most similar distributions was the Mahalanobis distance:

$${D}_{M}^{i}({H}^{i})=\sqrt{{({H}^{i}-{I}^{{\rm{LASY}}})}^{T}{S}^{-1}({H}^{i}-\,{I}^{{\rm{LASY}}})}$$

in which ${H}^{i}$ is a multivariate vector $({\rm{logit}}\left(\frac{{{\rm{mean}}}^{i}}{18}\right),\,{\rm{logit}}({{\rm{prop}}}_{0}^{i}))$ corresponding to a survey–age–sex–year entry in our educational database, *I *^LASY^ is a multivariate vector $({\rm{logit}}\left(\frac{{{\rm{mean}}}^{{\rm{LASY}}}}{18}\right),\,{\rm{logit}}({{\rm{prop}}}_{0}^{{\rm{LASY}}}))$ repre-senting the modelled entities described above, and *S *^−1^ is the covariance matrix between vectors ${\rm{logit}}\left(\frac{{{\rm{mean}}}^{i}}{18}\right)$ and ${\rm{logit}}({{\rm{prop}}}_{0}^{i})$.

For each *I* ^LASY^, *K* distributions with the smallest Mahalanobis distances are chosen as candidate distributions for the final ensemble distribution. To collapse *K* distributions to a final ensemble distribution, we use a weighted average of the candidate distributions based on a location, age, and cohort distance defined as:

$${{\rm{D}}{\rm{i}}{\rm{s}}{\rm{t}}{\rm{a}}{\rm{n}}{\rm{c}}{\rm{e}}}^{i}={({P}_{{\rm{a}}{\rm{g}}{\rm{e}}}\times {{\rm{D}}{\rm{i}}{\rm{s}}{\rm{t}}{\rm{a}}{\rm{n}}{\rm{c}}{\rm{e}}}_{{\rm{a}}{\rm{g}}{\rm{e}}}^{i})}^{\psi }\,+{({P}_{{\rm{c}}{\rm{o}}{\rm{h}}{\rm{o}}{\rm{r}}{\rm{t}}}\times {{\rm{D}}{\rm{i}}{\rm{s}}{\rm{t}}{\rm{a}}{\rm{n}}{\rm{c}}{\rm{e}}}_{{\rm{c}}{\rm{o}}{\rm{h}}{\rm{o}}{\rm{r}}{\rm{t}}}^{i})}^{\psi }+{({P}_{{\rm{s}}{\rm{p}}{\rm{a}}{\rm{c}}{\rm{e}}}\times {{\rm{D}}{\rm{i}}{\rm{s}}{\rm{t}}{\rm{a}}{\rm{n}}{\rm{c}}{\rm{e}}}_{{\rm{l}}{\rm{o}}{\rm{c}}{\rm{a}}{\rm{t}}{\rm{i}}{\rm{o}}{\rm{n}}}^{i})}^{\psi }$$

All values of *P* and Distance are rescaled to lie between 0.001 and 1. ${{\rm{Distance}}}_{{\rm{location}}}^{i}$ is 0.001 for same country, 0.33 for same region, 0.66 for same super-region, and 1 otherwise.

$${{\rm{Weights}}}^{i}=\frac{1}{{{\rm{Distance}}}^{i}}$$

*ψ* is a hyperparameter controlling how sharply weights decrease as Distance^*i*^ increases. To collapse *K* distributions to a final ensemble distribution for each LASY combination we calculated:

$${{\rm{Proportion}}}_{{\rm{eduyrs}}}^{{\rm{LASY}}}=\frac{{\sum }_{i=1}^{K}{{\rm{Weights}}}^{i}\times {{\rm{proportion}}}_{{\rm{eduyrs}}}^{i}}{{\sum }_{i=1}^{K}{{\rm{Weights}}}^{i}}$$

in which Proportion_eduyrs_ is the proportion in each educational bin, 0–18.

Final ensemble distributions were then smoothed by bin using a Loess smoother with a span of *η* over time to ensure plausible time series for each draw. All hyperparameters were optimized using out-of-sample predictive validity (detailed in the Supplementary Information), and chosen values include: *K* = 80; *P*_age_ = 0.25; *P*_cohort_ = 0.85; *P*_space_ = 0.7; *ψ* = 2.5; *η* = 0.5.

### Rate of change distribution forecasting model

To forecast the distribution of education and mean years of schooling, we use a rate of change (ROC) model at the single-year bin level. This has the benefit of producing projections of mean attainment that respect the nonlinear dynamics of distributional growth. The model is fit in a timeseries-specific fashion, separately by sex and country. For each single-year bin, we derive a ROC using a weighted average of the ROC for the last 15 years:

$${{\rm{ROC}}}_{{\rm{eduyr}}}^{{\rm{LAS}}}=\,\mathop{\sum }\limits_{i=2004}^{2018}\frac{{\rm{logit}}{({{\rm{proportion}}}_{{\rm{eduyr}}})}^{{{\rm{LAS}}}^{i}}-\,{\rm{logit}}{({{\rm{proportion}}}_{{\rm{eduyr}}})}^{{{\rm{LAS}}}^{i-1}}}{15}$$

Where ${{\rm{ROC}}}_{{\rm{eduyr}}}^{{\rm{LAS}}}$ is the average rate of change over the last 15 years within each location–age–sex (LAS) combination for each single-year bin of education (0–18).

The ROC model was leveraged only where the cohort extrapolation model could not inform our estimates. This begins in 2019 for 25–29-year-olds, 2024 for 30–34-year-olds, and 2029 for 35–40-year-olds. For the results presented in the main text, for 25–29-year-olds, this method was used for 2019 onwards.

### SDG progress and inequality metrics

Drawing on these estimates of the distribution of years of schooling, we calculate several metrics detailing global progress towards the SDG 4 targets. We calculate the proportion of the population of individuals age 25–29 who have completed primary, secondary, and tertiary education, defined as completing at least 6, 12, and 15 years of schooling, respectively. We describe gender equality using the ratio of female to male attainment of primary and secondary education, as well as the gap in mean years of schooling between men and women. Aggregate measures at the national level for both sexes, and at the regional level were calculated, using projected population estimates drawn from the World Population Prospects dataset^35^. We also present a novel index of educational inequality among young people in each country, the average AID. This index is defined as the average value of the absolute differences between all possible pairs of individuals in the population. The AID is also mathematically equivalent to the Gini coefficient, multiplied by two times the mean of the distribution^36^.

### Predictive validity

The main aims of this analysis are predictive in nature, and we therefore assessed each stage of our model, and each model selection decision, with respect to predictive capacity. We focused mainly on ‘out-of-sample’ predictive ability, which reflects how well the model predicts data that was not directly available. This most mimics the true task that we want our model to accomplish, that is, to make accurate predictions for the geographies and time periods that do not have input data available. To assess out-of-sample predictive validity, we followed the general strategy of dividing our database into ‘training’ and ‘testing’ data. The model was fit on the training data, and the results were compared with the testing data. The ‘error’ of the model represents the average amount that our model was incorrect compared with the ‘true’ data that was held out. Each step of the modelling process was assessed for how well it predicted (out-of-sample) the mean years of schooling for a given population, as well as other aspects of the distribution, such as the proportion with 0 years of schooling. We also assessed the degree to which predictive validity varied by time period, across regions, and by which type of data source was held out. There were small differences in predictive validity across these dimensions, for example, models tended to perform slightly better in the 2000–2018 period where the most data are available; however, they were generally modest. Furthermore, we found that the best performing models tended to perform optimally across almost all geographies/time periods, so it was not necessary to use multiple models for a single step. All predictive validity results, and a discussion of their implications, can be found in the Supplementary Information.

### Reporting summary

Further information on research design is available in the Nature Research Reporting Summary linked to this paper.

## Online content

Any methods, additional references, Nature Research reporting summaries, source data, extended data, supplementary information, acknowledgements, peer review information; details of author contributions and competing interests; and statements of data and code availability are available at 10.1038/s41586-020-2198-8.

## Supplementary information

Supplementary Information This file contains supplementary text describing results and discussion of estimates presented with alternative inequality metrics, comparisons to previous education modelling efforts, model selection and predictive validity, Supplemental Figures 1-15, Supplemental Tables 1-3, and Supplementary References.

Reporting Summary

## Source data

Source Data Fig. 1

Source Data Fig. 2

Source Data Fig. 3

## Figures and Tables

**Extended Data Fig. 2:**
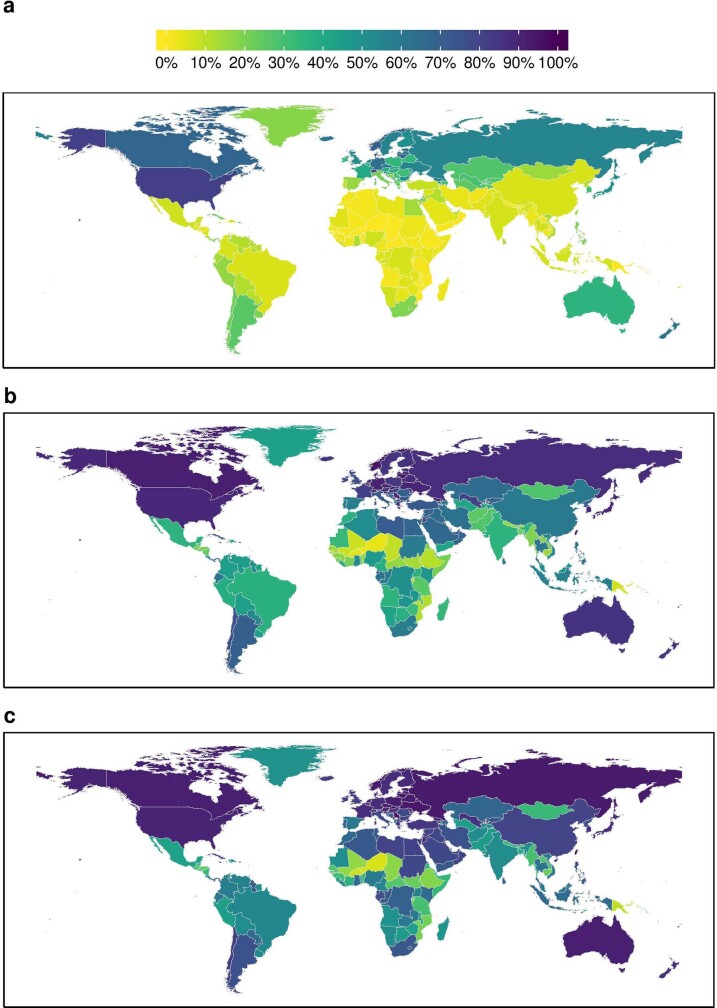
Completion of 12 or more years of schooling. **a**–**c**, The percentage of the population aged 25–29 completing at least 12 years of schooling is shown by country, for 1970 (**a**), 2018 (**b**), and 2030 (**c**). Maps were produced using R v.3.5.0.

**Extended Data Fig. 3:**
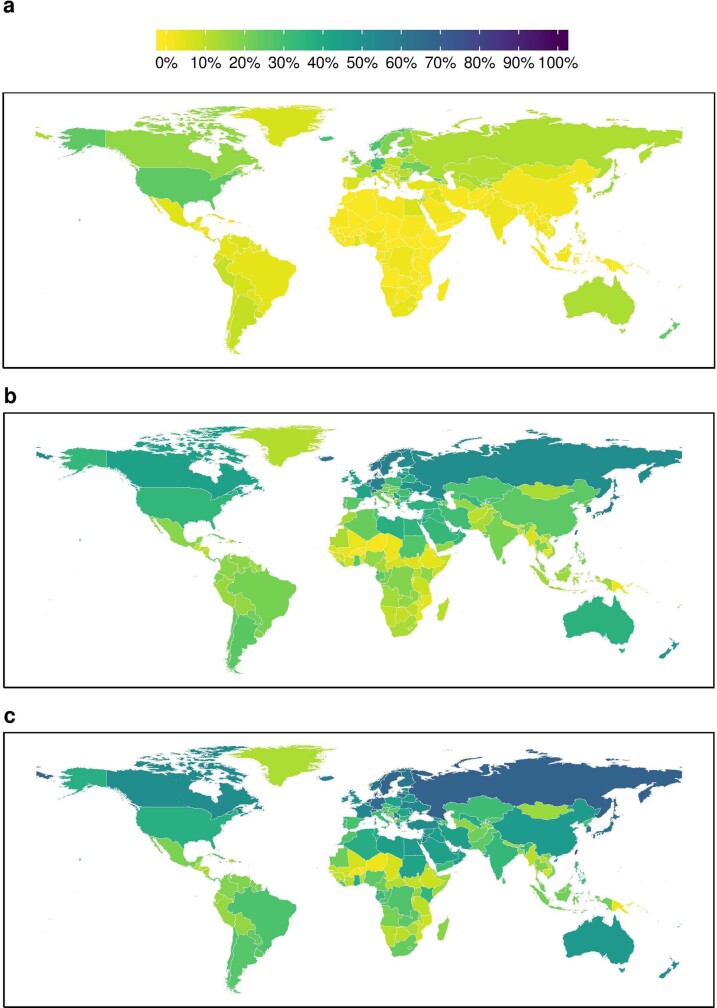
Completion of 15 or more years of schooling. **a**–**c**, The percentage of the population aged 25–29 completing at least 15 years of schooling is shown by country, for 1970 (**a**), 2018 (**b**), and 2030 (**c**). Maps were produced using R v.3.5.0.

**Extended Data Fig. 4:**
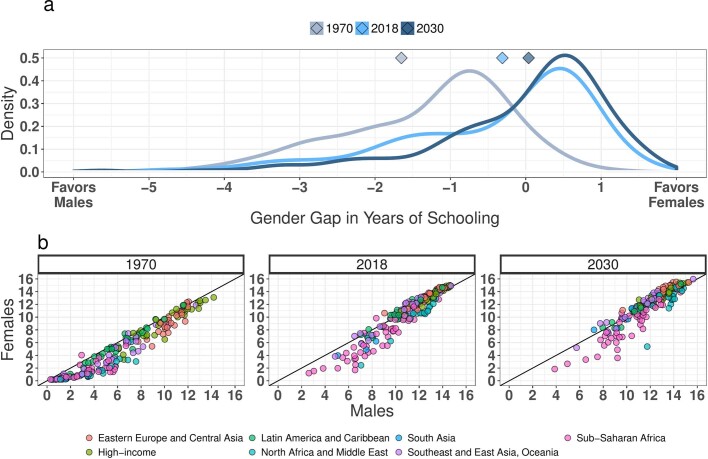
Years of schooling among men and women. **a**, The distribution of the gap in mean years of schooling between men and women, aged 25–29, is shown for 1970, 2018, and 2030, with the population-weighted mean for each time point represented with a diamond. Means were calculated over modelled estimates from *n* = 195 countries. **b**, Years of schooling is represented for men on the *x* axis and women on the *y* axis for 1970, 2018, and 2030, in which each point indicates the value for one country, colour-coded by regional grouping. A point above the line indicates additional schooling for women relative to their male counterparts.

## Data Availability

This study used data that are available from public online repositories, most of which require a straightforward registration process and usage agreement with the data provider. A detailed table of data sources and availability can be found in the Supplementary Information. Although the authors are restricted from providing the data directly in most cases, specific datasets may be made available by request and with permission from the data provider. The authors may be contacted for assistance in acquiring data for the replication of this study. All maps presented in this study have been produced by the authors and no permissions are required for publication. Administrative boundaries were retrieved from the Global Administrative Unit Layers (GAUL) dataset^37^.

## Code availability

All code used for these analyses is available here: https://github.com/Joseph-Friedman/education_inequality.
