# Supplementary material for: Measuring and forecasting progress towards the education-related SDG targets
Source: Nature. 2020 Apr 15;580(7805):636–9. doi: 10.1038/s41586-020-2198-8 (PMC7332421; doi:10.1038/s41586-020-2198-8)
Supplement: Supplementary file 1 — This file contains supplementary text describing results and discussion of estimates presented with alternative inequality metrics, comparisons to previous education modelling efforts, model selection and predictive validity, Supplemental Figures 1-15, Supplemental Tables 1-3, and Supplementary References. [file 41586_2020_2198_MOESM1_ESM.pdf]

nature

https://doi.org/10.1038/s41586-020-2198-8

Supplementary information

Measuring and forecasting progress towards the education-related SDG targets

In the format provided by the authors and unedited

Joseph Friedman, Hunter York, Nicholas Graetz, Lauren Woyczynski, Joanna Whisnant, Simon I. Hay & Emmanuela Gakidou ✉

Supplemental Information for "Measuring and forecasting progress towards the education-related SDG targets"

Contents

Supplemental Discussion....................3

Choice of Inequality Metric – Theoretical Framework....................3

Supplemental Figure 1. The Relationship Between Mean Years of Schooling and Inequality Metrics 3

Supplemental Figure 2. Global Trajectories in Educational Inequality and Mean Attainment by Metric....................4

Choice of Inequality Metric – Comparison of Results....................5

Supplemental Figure 3. Global Trajectories in Educational Inequality Over Time by Metric....................5

Supplemental Figure 4. "Most Unequal" Societies Under Gini and AID Assumptions....................7

Choice of Inequality Metric – Conclusions....................7

Comparison to Previous Education Modeling Efforts....................8

Correlation to Previous Education Modeling Efforts....................9

Supplemental Figure 5. Mean Years of Schooling from Barro-Lee and Current Study....................10

Supplemental Figure 6. Mean Years of Schooling from Wittgenstein Centre and Current Study....................11

Limitations....................11

Model Selection and Predictive Validity....................12

Data Adjustment Model....................13

Supplemental Figure 7. OOS-PV Statistics for Source-Type Adjustment Candidate Models....................14

Supplemental Figure 8. OOS-PV for Source-Type Adjustment Candidate Models by Decade and Task 15

Cohort Extrapolation....................16

Supplemental Figure 9. OOS-PV for Cohort-Extrapolation Candidate Models....................16

Supplemental Figure 10. OOS-PV for Cohort-Extrapolation Candidate Models by Decade....................17

Ensemble K-Nearest Neighbors Distribution Model....................18

Supplemental Figure 11. OOS-PV for KNN Candidate Hyperparameter Specifications....................18

Forecasting Model....................19

Supplemental Figure 12. OOS-PV for KNN Candidate Forecasting Models....................20

....................22

Supplemental Figure 13. Other Dimensions of OOS-PV Considered for Forecasting Models....................22

Supplemental References....................23

Supplemental Tables and Figures 26

Supplement Table 1. Country Specific Indicators for 1970,2018, and 2030.................................... 39

Supplement Table 2. Country Specific Data Sources by Year and Data Provider 51

Supplement Table 3. Country- and Region-Specific Gold Standard Data Providers.......................... 56

Supplemental Figure 14. Country Specific Gender Gaps in Years of Schooling................................ 60

Supplemental Figure 15. Country Specific Kuznets Curves... 64

Supplemental Discussion

Choice of Inequality Metric – Theoretical Framework

The discussion of which metric inherently best captures inequality is epistemological in nature[1]. Previous assessments of inequality metrics for educational attainment have been limited by data availability[2], or have tended focused on single metrics, most often the Gini coefficient or standard deviation[3,4]. We draw on comprehensive estimates of the distribution of education to assess which inequality metrics can function reasonably in the representation of educational inequality. Supplemental figure 1 details the relationship between average years of schooling and a number of inequality metrics. Both the Gini coefficient and the average interpersonal difference (AID) belong to the family of inter-individual difference metrics[5,6], which are calculated via the generalized formula shown below:

$$\mathrm{IID}(\alpha, \beta) = \frac{\sum_{i=1}^{n} \sum_{j=1}^{n} |e_i - e_j|^{\alpha}}{n^2 + (2 * \bar{e})^{\beta}}$$

Where β indicates the degree of to which differences are absolute, or relative to the mean of the population, and α indicates the increased degree of increased weighting of outliers. For the Gini coefficient, both α and β are set to 1, while the AID has an α of 1 and a β of 0. Therefore, the Gini coefficient is equal to the AID, divided by two times the mean. The standard deviation compares the value of all individuals to the mean, rather than other individuals. The coefficient of variation is estimated as the standard deviation divided by the mean value. Finally, the ratio of the 90th percentile to

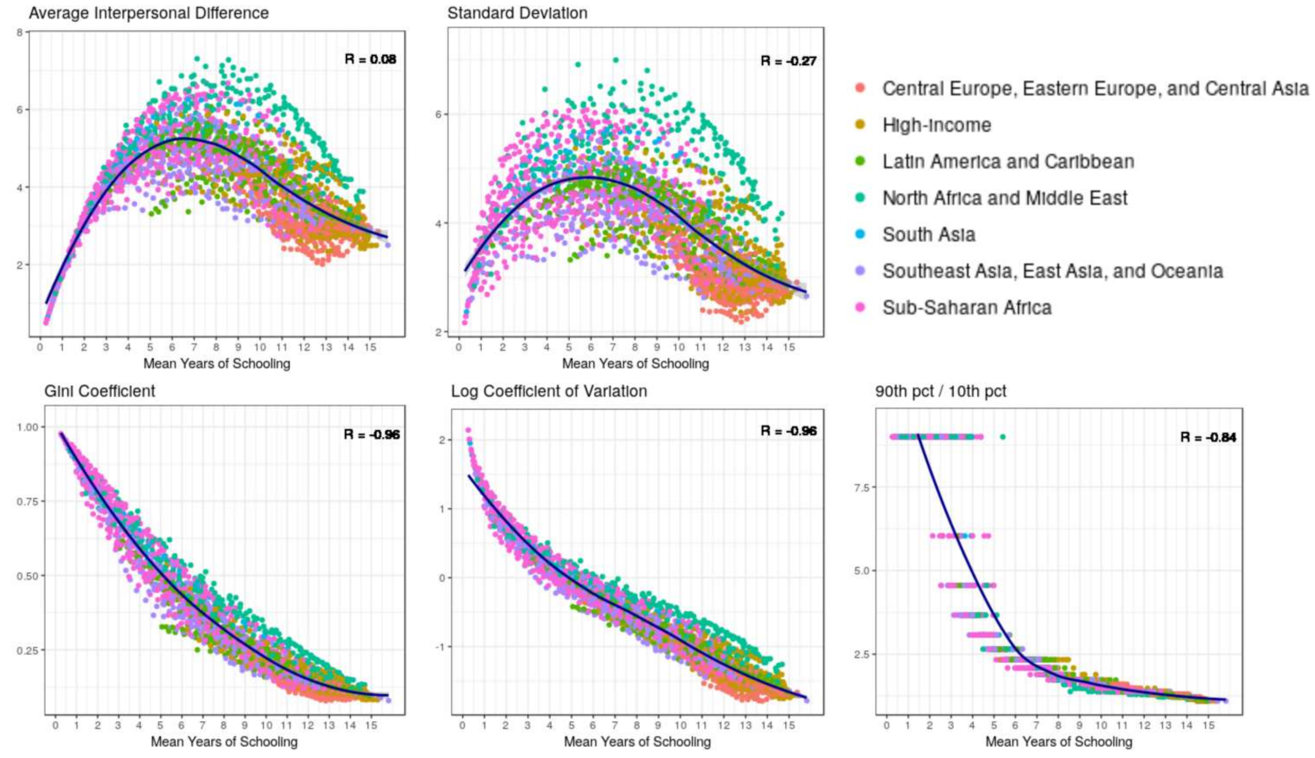

Supplemental Figure 1. The Relationship Between Mean Years of Schooling and Inequality Metrics

Points are plotted for every 5th year, 1970 through 2030, and represent national values for individuals age 25-29. Color indicates super-regional grouping. Loess lines of best fit are added to show central tendency. Pearson's r correlation coefficients are shown for each relationship and were calculated over n=11,895 country-years of modelled estimates.

the 10th percentile is another commonly used relative inequality metric, that does not use the mean of the distribution. For variables such as per-person income that vary highly between contexts, a relative measure such as the Gini coefficient is an intuitive choice. In phenomena that do not vary greatly between contexts, researchers could potentially consider absolute measures, like the standard deviation, as more appropriate.

In the first stage, we explore the relationship between each metric and average years of schooling. This is a useful heuristic to understand how each metric behaves over the observed range of average values. We find that relative measures of inequality, such as the Gini coefficient, coefficient of variation, or 90th to 10th percentile ratio are highly collinear with average years of schooling, with correlation coefficients of -0.96, -0.96, and -0.84 respectively. This is expected, as relative metrics like the Gini coefficient have the mean value of the distribution in their denominator. Therefore, in the context of a highly bounded indicator like education, this effect induces very high correlation. Highly uneducated populations have high Gini coefficients as only a few individuals have more than zero years of schooling. As populations become more educated, their Gini coefficient heads steadily towards zero, with minimal variation between populations. Absolute measures of inequality, such as the AID, have more variable relationships with average attainment. Overall the correlation coefficient between the AID and mean years of schooling was 0.08. Employing either the AID or the standard deviation provides estimates of inequality that vary significantly more for a given mean years of schooling compared to average measures of inequality.

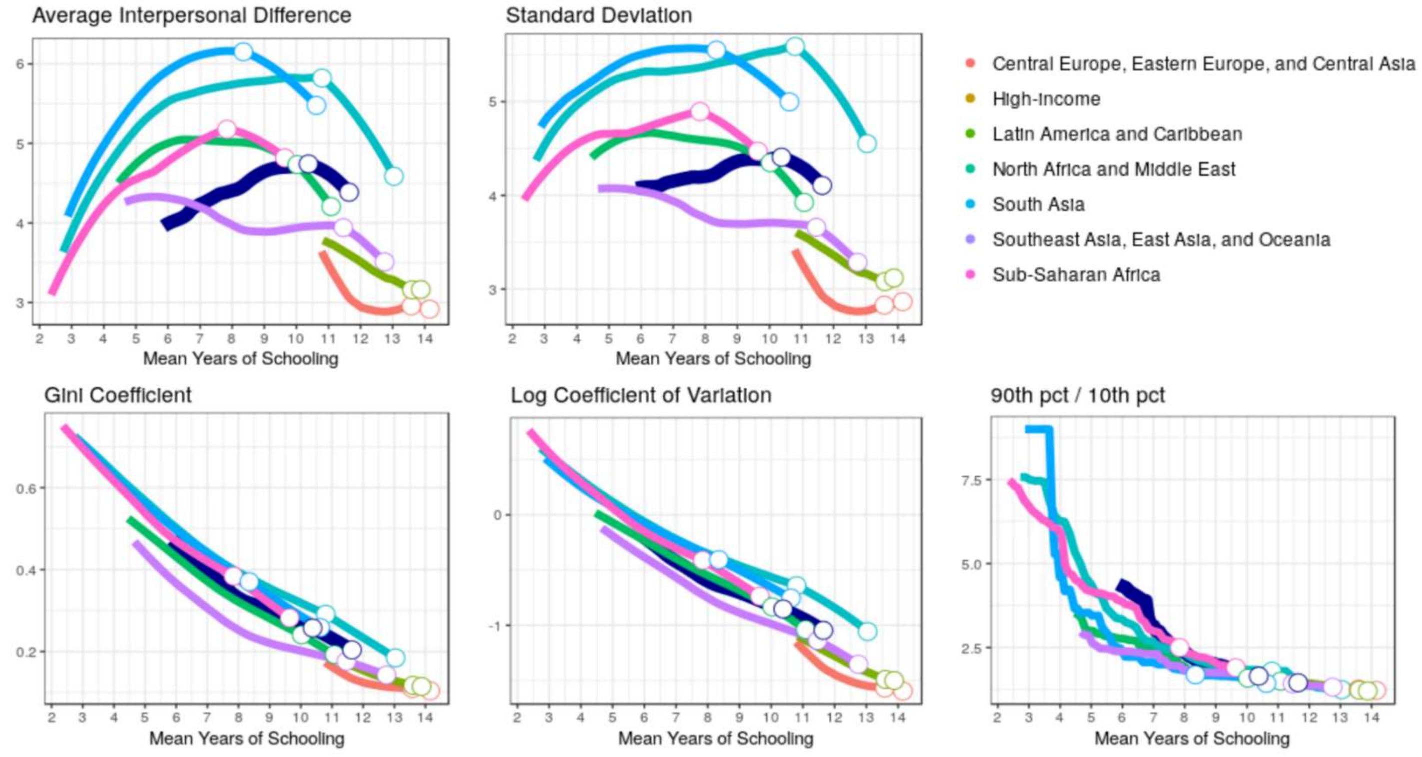

Supplemental Figure 2. Global Trajectories in Educational Inequality and Mean Attainment by Metric

Educational inequality represented by a number of metrics is represented on the y axis against the mean years of schooling globally and for each super-region. The dots mark 2018, the beginning of forecasts, and 2030, the SDG target year. Regional trends were aggregated from n=195 national level modeled estimates.

Choice of Inequality Metric – Comparison of Results

Supplemental figures 2 and 3 show how the choice of inequality metric affects conclusions drawn about the global and regional trajectories of inequality in education. Both the standard deviation and the AID show highly similar trends in global and regional inequality, as described in detail in the main text. Kuznets curves are observed for nearly all geographies, though there is tremendous difference in the height achieved by each curve. The relative inequality metrics show maximal inequality in 1970 when mean attainment levels are lowest and exhibit sharp and steady declines through 2030 as mean attainment rises globally.

In comparing the AID and Gini coefficient as metrics of inequality, it is important to consider that for a given mean value, the variability between geographies shown by either metric is identical. Given that the mean is in the denominator of the Gini coefficient, at, for example, 12 years of mean attainment, the rank and magnitude of variation between each region observed in figure 2 is identical. The choice of metric, therefore, is most influential in comparing inequality over time and ranges of mean attainment values. Here we must make a normative decision around which societies appear most and least "unequal" according to our interpretation of the concept[1]. If we were to accept the implications of the

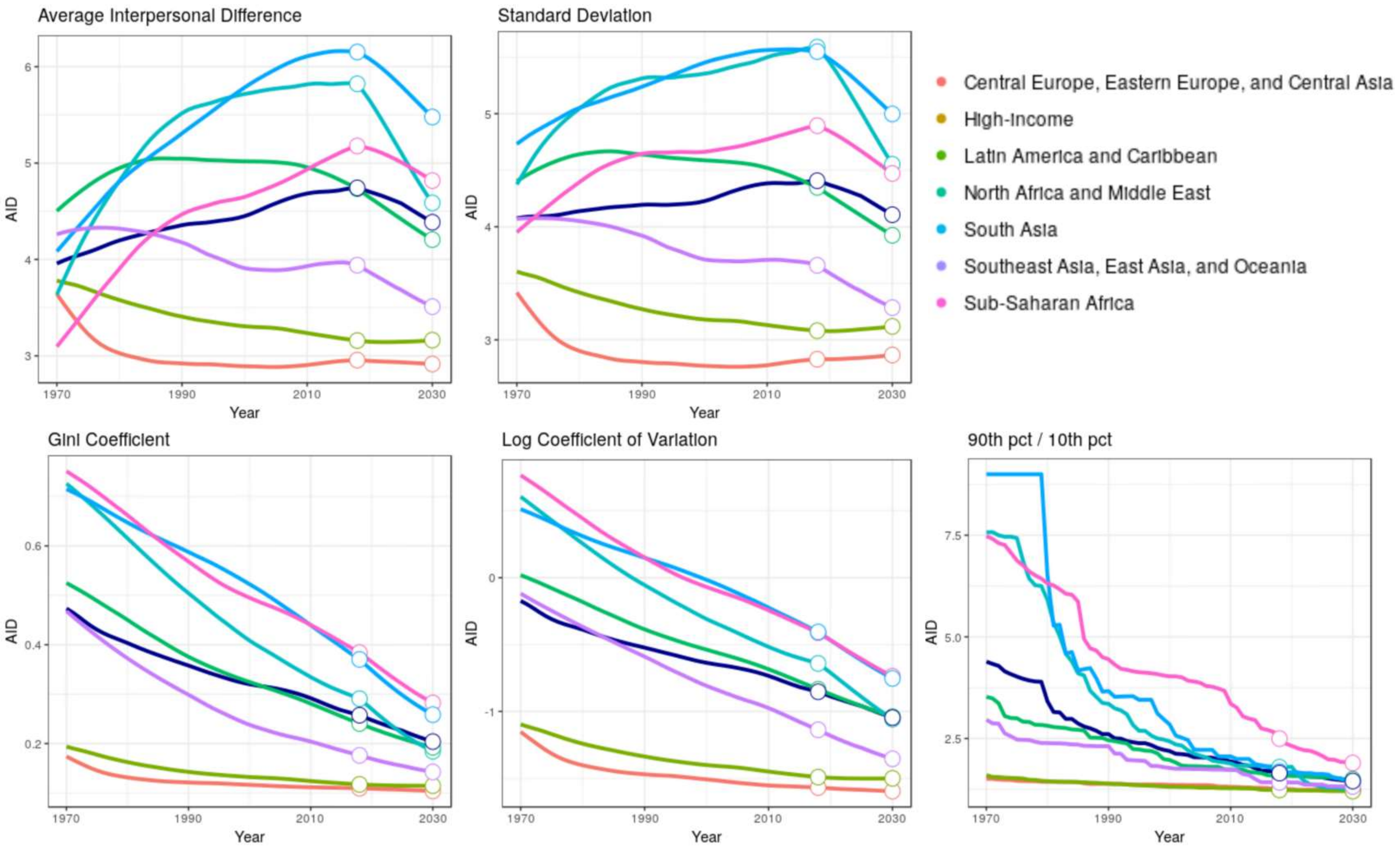

Supplemental Figure 3. Global Trajectories in Educational Inequality Over Time by Metric

Educational inequality represented by a number of metrics is represented on the y axis over time globally and for each super-region. The dots mark 2018, the beginning of forecasts, and 2030, the SDG target year. Regional trends were aggregated from n=195 national level modeled estimates.

Gini coefficient, we would conclude that the most unequal nations observed by our study are those that are just beginning to educate their populations, those that have the lowest mean attainment values and just a few individuals achieving any years of schooling. This kind of distribution of education was commonplace among low-income nations in 1970, however by 2018 all nations had at least begun to educate their population to a meaningful degree, and most nations appeared to be near the peak of their Kuznets curve, if not significantly further along it. If we consider the AID metric, however, we would conclude that the most unequal societies are those developing nations that have both a high proportion of the people that still receives little to no schooling, and another large segment that receives many years of schooling. Supplemental Figure 4 illustrates the kind of societies that are deemed “most unequal” under either metric. The figure is best interpreted alongside supplemental figure 1 for context. Under the Gini coefficient assumptions, Niger in 1970 would be considered a “maximally unequal” context, with a Gini of .98, because almost 0% of the population has any schooling. The few years of available schooling is attained by a small number of individuals, who virtually all have fewer than 7 years. An example “maximally unequal” society under the AID assumptions is exemplified by Yemen in 2018. Here we can see an example of a society where over 30% of the population of 25-29-year-olds still has zero schooling, while over 40% of the same population has 12 years or more of schooling. As stated earlier, the choice of metric is normative. The authors of this study consider that Yemen in 2018 has much greater inequality than Niger in 1970 and have chosen the AID metric as the measure by which to present results in this paper. Recognizing that it is unlikely that consensus can ever be reached on which is the best measure of inequality, regardless of the application of that measure, we present results for alternative measures in this Supplement and also make available the results of our entire database which makes it easy for other researchers to produce the measure of inequality that they believe is most relevant to their research endeavors.

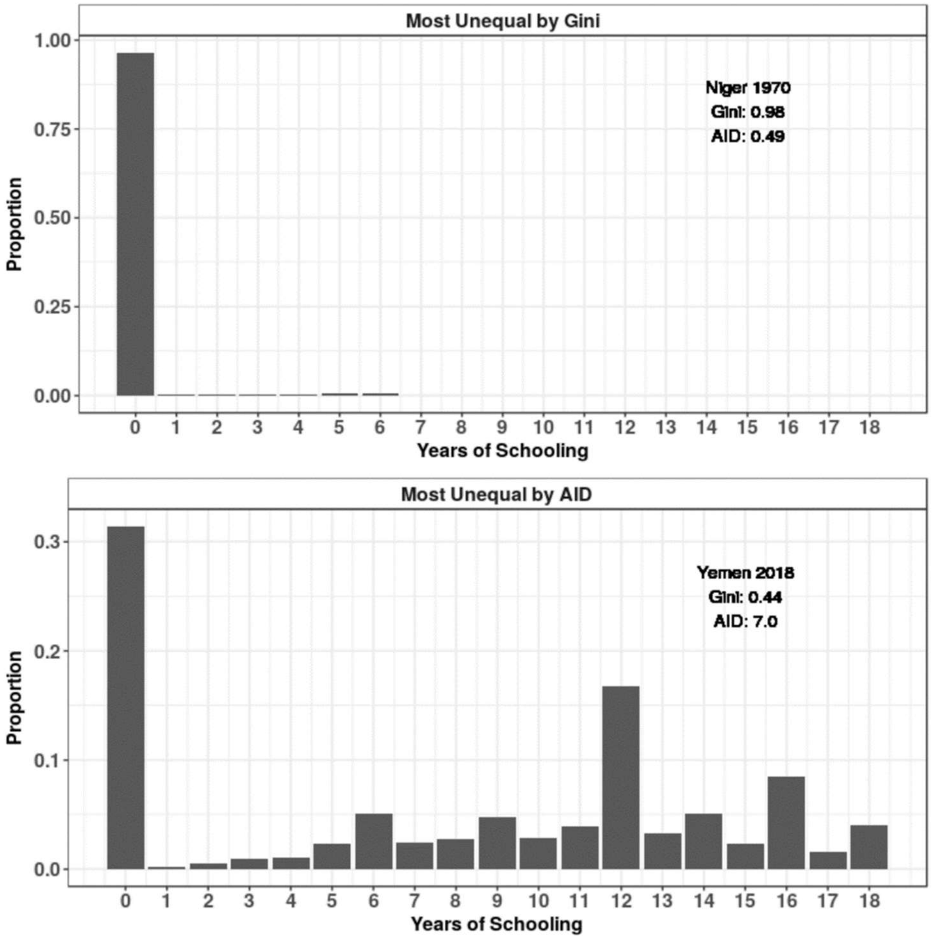

Supplemental Figure 4. "Most Unequal" Societies Under Gini and AID Assumptions

The distribution of years of schooling for two example populations illustrating "maximally unequal" populations under the assumptions of the Gini and AID metrics. Each panel reflects individuals age 25-29, for a single country-year. This figure is best interpreted alongside supplemental figure 1 for context.

Choice of Inequality Metric – Conclusions

Ultimately, we chose to use the AID as our measure of educational inequality in the main text of this study. We believe that for education, an absolute metric such as the standard deviation or AID is the most policy-relevant measure of inequality. In considering the range of distributions we observed in our study, we concur with the interpretation yielded by the AID metric, that the most unequal societies, in terms of education, are those at the peak of their Kuznets curve that have large proportions of individuals with zero schooling, and large proportions with post-secondary education. Given that we believe that a metric of inequality provides an additional measure for policy makers to care about and aim to improve upon, we find that the AID is the most policy relevant metrics as at a given level of educational attainment it captures adequately how unequally the distribution years of schooling within a given population is.

We suggest that the degree of relativity of an inequality metric, which can be changed by modifying the value of β in the equation above, should reflect the inherent boundedness of a measure. For income,

which has hyperbolic variation between countries, using a β of 1 is intuitive. Likewise, for years of schooling, which is limited in range by the human life course, a β value of 0 is a reasonable choice. For other intermediately-bounded measures, employing a set of inequality metrics with a range of β values would be advisable.

For education, we ultimately chose to use the average interpersonal difference as the measure of educational inequality to present results by, as we find that the quantity it represents—the average difference in educational attainment between any two individuals in a population—to be easy to understand and relevant both to our conceptualization of inequality and for policy makers. Given that the mean is "in the denominator" of the Gini coefficient, expanding the mean level of schooling will almost always reduce measured inequality, regardless of if it occurs among those who are best or least off. The AID on the other hand, changes depending on which part of the population is targeted for progress. This attribute makes it a more policy-relevant metric as it tracks, as countries are investing in education and increasing the mean years of schooling of their populations, how well they are doing in terms of distributing those years of schooling equally.

Comparison to Previous Education Modeling Efforts

Here we summarize major alternative education modeling efforts, and discuss differences in methodology, scope, and results and their interpretation. Two sets of education estimates that are used extensively and are closest in scope to the work presented in this study, include the work by Barro and Lee and the work by the Wittgenstein Centre.

The estimates produced by Barro and Lee are among the most highly cited, have pioneered many aspects of measuring educational attainment, and have been updated several times [7–10]. Their framework involves drawing mainly on census data and using a timeseries approach. They model broad bins (categories) of educational attainment, as defined by the ISCED classifications[11], and subsequently calculate means years of schooling using standard duration assumptions. As we have shown previously, the standard duration method is useful in enforcing consistency between mean years of schooling and the modeled distribution of education, however it does include some assumptions that can introduce bias and error into the estimates[12]. Recent estimates draw on about 600 total sources and cover 146 countries from 1950 through 2010.

A body of work produced by the Wittgenstein Centre has a distinct approach to modelling educational attainment that draws on demographic (cohort component) models[13–15]. This entails drawing on a recent data source for each country from a year close to the analytical base year, most recently 2015[15]. One base dataset is chosen per country, for a total of about 200 sources. Populations are then forecasted to 2100 and backcasted to 1950 using a number of expert-driven and empirical assumptions about education, fertility, migration, life expectancy, and other key factors. This approach is highly useful for studying the interconnection of education with other aspects of development, especially population growth. This kind of model allows for explicit examination of policy-levers for changing population-level dynamics, and holds key insights for the scientific understanding of population development, with implications for policy-makers[16]. Among other assumptions, the modeling framework generally assumed "global convergence" in forecasts, where all countries slowly drift towards global averages of parameters. Education is represented by a number of bins, including complete and incomplete attainment at numerous levels, as defined by ISCED standards.

Our work differs from these two major previous efforts in a few important ways that reflect its central aim of serving as a benchmarking and forecasting exercise towards the education-related SDG targets. We include 3,180 census and survey data sources, which represents the largest database of education data to our knowledge. Given the known sampling and non-sampling error in measures of education[17,18] we opt to include all possible sources of data and leverage a modeling framework that synthesizes disparate data using sampling variance and between-source deviation to estimate final results. Our data adjustment approach offers an additional correction for bias between data providers, creating a set of more directly comparable estimates. Including all possible data entails both advantages and challenges compared to other estimation strategies. The Wittgenstein approach benefits from internal consistency, as well as flexibility in the calculation of forecasting scenarios under varying assumptions. However, it has a limited capacity to automatically include multiple data sources across the available timeseries. By only using one data source, these estimates make the assumption that the data source chosen is valid and reliable and does not suffer from any non-sampling error. Alternatively, our approach allows for the inclusion of temporally overlapping data from numerous data providers, as well as data from multiple time periods. As a prediction effort, our work is more similar to that of Barro and Lee, using a timeseries modelling approach, although the details of our model vary substantially. We use nearly five times the input data, and model the full single year distribution of education, which better captures drop-out patterns relative to descriptions using wider bins of attainment, and allows for a more precise measurement of educational inequality[12]. We also provide projections through 2030, and novel metrics of educational inequality, that are focused on benchmarking regional and national progress towards meeting the education-related SDG targets.

The forecasts produced by the Wittgenstein Centre are highly useful in understanding and quantifying how investments in education will ripple out through other domains of development. For instance, the "SDG Education Scenario" allows us to appreciate how impactful achieving the education-related SDG targets could be for global health and well-being[15]. On the other hand, our work is a descriptive effort seeking to provide timely evidence on which countries are currently on track to achieve these important goals, and which countries have disparities that require renewed investments and attention. We employ a framework that is designed to maximize country-level predictive power, including an unprecedented level of data coverage and predictive validation for the field. Though our work is not directly comparable to the Wittgenstein forecasted scenarios (in methodological approach, the actual indices measured, and the main aims of the estimates), it is highly complementary. We produce country-level evidence about trajectories towards meeting international education targets, and the Wittgenstein scenarios provide strong evidence about the importance of meeting these goals for myriad areas of global development.

Correlation to Previous Education Modeling Efforts

In addition to out-of-sample predictive validity, the reliability of our estimates was assessed by comparing the mean years of schooling predictions to previously published estimates. A comparison to previous estimates of the distribution of education, or educational inequality, was not possible, given the lack of a comparable set of numbers. Other efforts to model the distribution of education have measured attainment using bins such as "primary completed." These bins measure ISCED levels of attainment, rather than a specific number of years that is comparable between countries[13]. Our analysis is focused on number of years, and therefore a direct comparison is not possible. Nevertheless, we were able to compare our mean attainment estimates to previous work, as a general measure of the similarity of data and modeling outcomes. We used the most recent age and sex-specific estimates from Barro and Lee, as well as the Wittgenstein Centre, as comparators [19,20].

Previous studies have documented systematic differences in the data preparation process between the Barro-Lee numbers and our process, that do result small but systematic difference in the numbers[12]. Nevertheless, a high degree of correlation was expected, and was observed. Overall, comparing the data provided at 5-year intervals from 1970 to 2010, a correlation coefficient was observed of 0.94. Supplemental figure 5 shows country-year-age-sex specific data from both series of estimates.

Supplemental figure 6 compares mean years of schooling values between the estimates from the Wittgenstein Centre and the current study. It is important to note that mean years of schooling is not the main outcome of our current work, which is focused on other metrics of the distribution and inequality in education that are more relevant to SDG targets. Nevertheless, comparing this feature of the distribution of education—the mean—may serve as a helpful heuristic in understanding some of the methodological differences between our work and other education modelling exercises, and the implications for the interpretation of results. We compare our estimates to the Wittgenstein Medium (SSP2) education/population growth scenario which assumes a continuation of recent trends and global convergence. For the in-sample period close to the base year of the Wittgenstein estimates our results are highly concordant, with correlation coefficients over .9 in every 5-year increment observed. However, we observe that our approach produces slightly more optimistic results in the forecasts than the baseline Wittgenstein scenarios. Importantly, we also show a wider range of country-level values, with a greater degree of outliers, which is logical in the absence of convergence assumptions. We feel that this is appropriate for a country-level benchmarking and forecasting exercise. Nevertheless, we

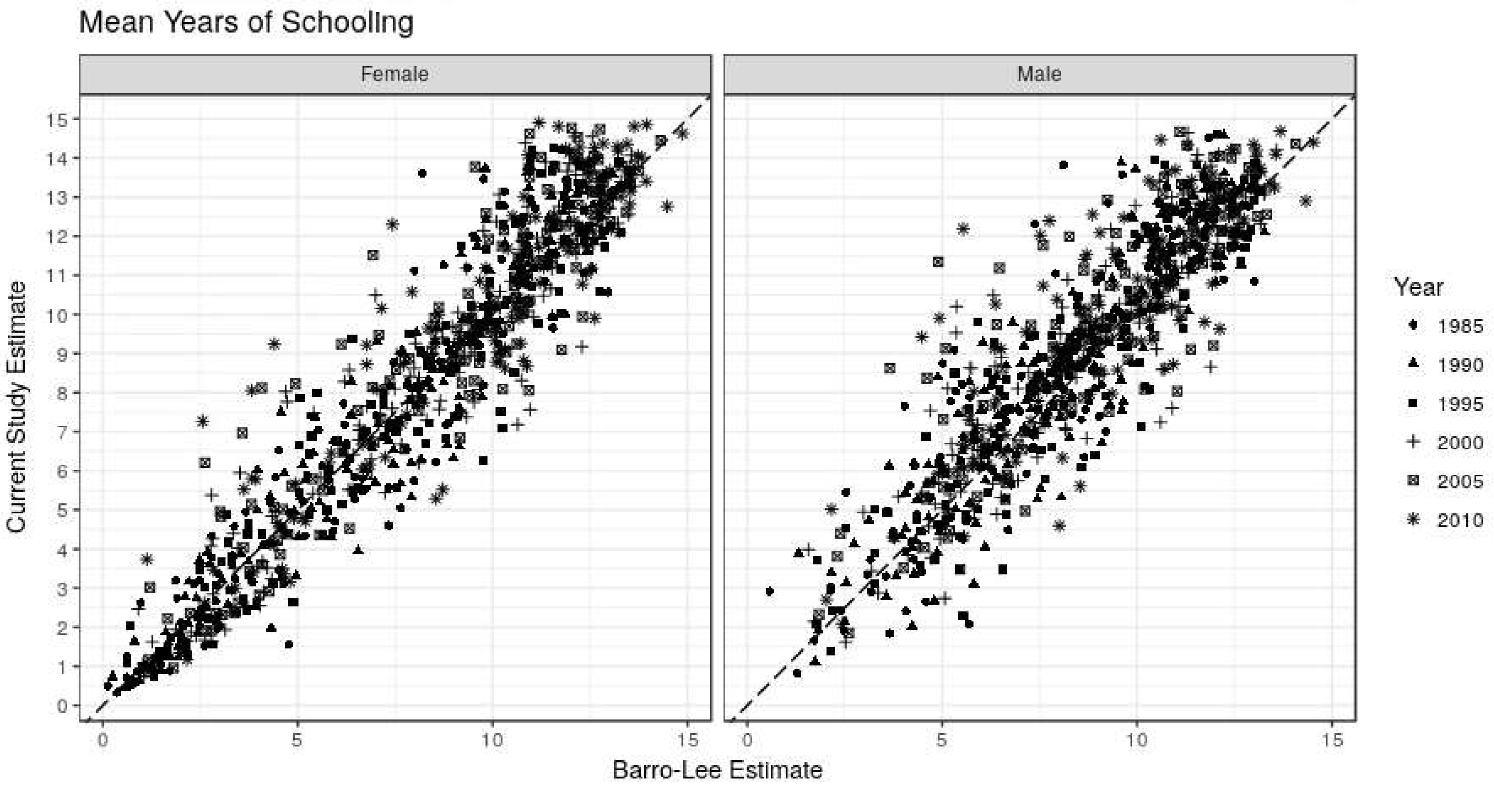

Supplemental Figure 5. Mean Years of Schooling from Barro-Lee and Current Study

Each point represents the average years of schooling for a country-year-age-sex group with overlapping data between the Barro and Lee 2013 estimates and the current study. All values reflect individuals age 25-29.

note that our estimates do show a significant degree of global convergence in the forecasted results, which is to be expected as countries approach inherent limits in bounded phenomena.

Finally, we reiterate that this exercise is not meant to be a comprehensive comparison between our work and prior efforts to model the distribution of education. The main outcome measures and contributions of our work are not directly comparable. Nevertheless, these limited comparisons may shed some light on systematic differences in methodological approach and the interpretation of results.

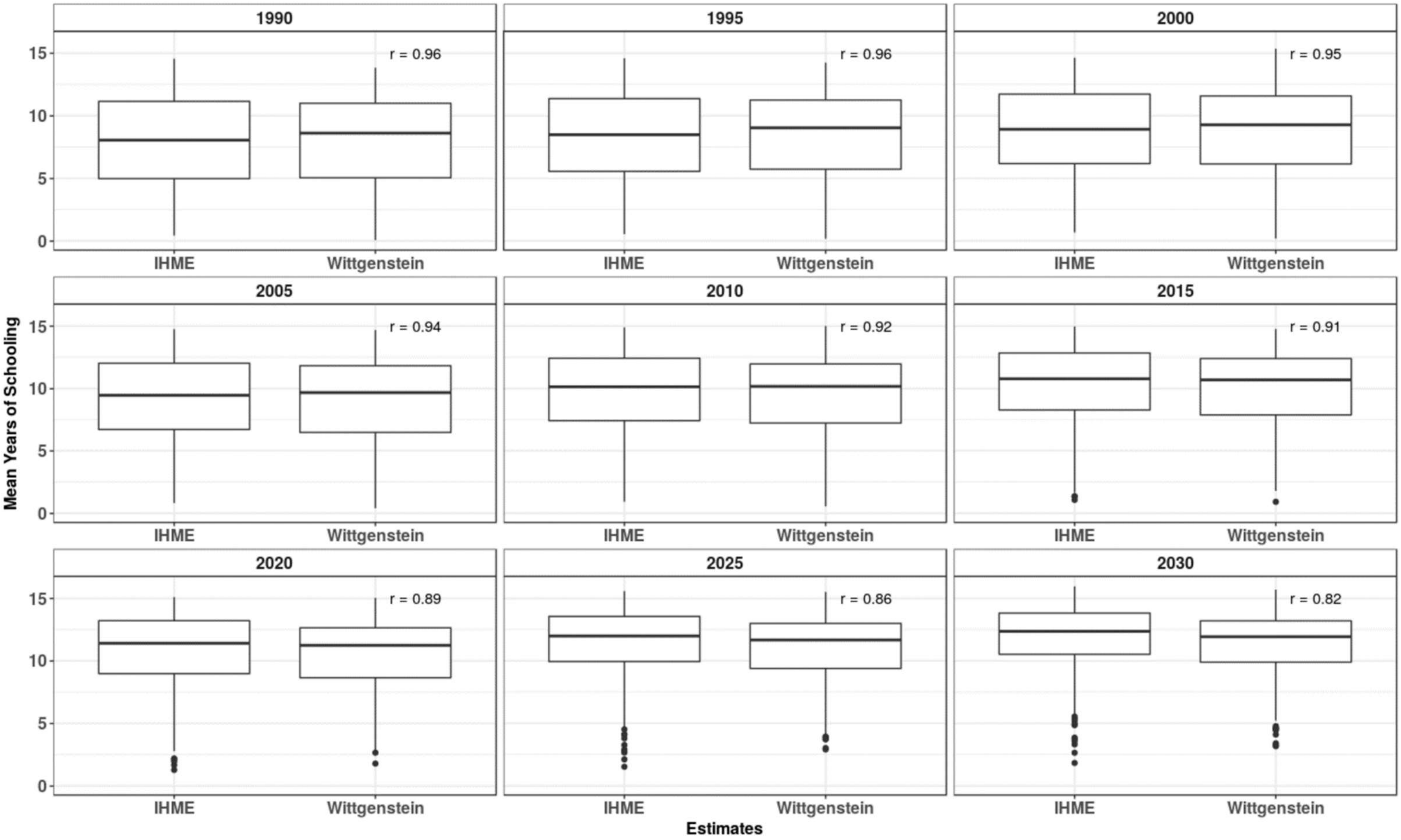

Supplemental Figure 6. Mean Years of Schooling from Wittgenstein Centre and Current Study

Each boxplot represents the distribution of average years of schooling for a country-year-age-sex group with overlapping data between the most recent Wittgenstein Centre estimates and the current study. All values reflect individuals age 25-29. The box and central line represent the 25th, 50th (median) and 75th percentiles of each distribution. The top whisker (vertical line) represents the largest value equal to or lower than 1.5 times the inter-quartile range plus the 75th percentile. The bottom whisker represents the smallest value equal to or greater the 25th percentile plus 1.5 times the inter-quartile range minus. Values outside of this range are shown with a point. The distribution of mean values is shown across countries and is not weight by population size. Pearson correlation coefficients are shown for each year and were fit on n=348 country-sex-year specific estimate pairs for years before and including 2010, and n=374 country-sex-year specific estimate pairs for the year 2015 and after.

Limitations

Creating comparable estimates of education is made more challenging by the great degree of heterogeneity in national education systems. For example, instead of modeling attainment of

“secondary education” using country-specific standards than can vary widely from 8 to 13 years, we employ globally standardized metrics of the number of individuals completing 12 years of schooling. These have the advantage of creating comparable metrics of attainment between countries but are not able to acknowledge particularities between schooling systems, and capture variations in the social capital that 12 years of education may confer in a given country. Our estimates also do not reflect the quality of education, simply the quantity of years attained. Schooling hours and quality of instruction vary between and within countries[8,9,21,22], which is an important mediator of the importance of education for health and development not accounted for here[23–25]. We also do not include the importance of non-traditional sources of education, such as open online courses[26], or other forms of learning outside of formal classroom environments.

Our analysis, like any exercise in prediction, is also limited by data availability. Data quality and the number of available data points differ by country. Therefore, we leverage regional information to make predictions about temporal trends in many countries. We may consequently be missing important country-specific trends in data-sparse areas. Nations with smaller populations and less developed economies tend to have more data gaps. These gaps are reflected in wider prediction intervals, which should be considered in the interpretation of results. We also explicitly examine our ability to reliably extrapolate using out-of-sample predictive validity (see supplement).

Our projections assume that trends observed in recent decades will continue into the future. We do not leverage expert knowledge or model the capacity of the education system directly. This approach may fall short in certain cases when inherent limitations of the educational system, e.g. the number of universities, may represent unobserved covariates that will lead to unexpected changes in trends. Nevertheless, a comparison to previous efforts modelling education for the set of overlapping countries and years shows a high degree of concordance between our estimates and previous measures. It is also important to note that international migration is an important factor that could affect the interpretation of our results. Differential migration by level of education could, for example, change the apparent equality of education in a country, regardless of how equally the educational system of that country actually operates. Finally, it is important to consider that most data sources conflate sex and gender and we are unable to differentiate between these different dimensions in a meaningful way in this work.

Model Selection and Predictive Validity

In order to determine the hyperparameters and model specifications for each model in our analysis, including source type adjustment, cohort extrapolation model, K-nearest neighbors algorithm, and forecasting models, we used several out-of-sample predictive-validity (OOS-PV) tasks[27–31]. To test how well each model or hyper-parameter set performed, we “knocked-out” a portion of the training database and used the remaining data to make predictions that could be compared directly to the known values. Given that each model varied in its purpose, holdout structures were constructed in a model-specific fashion to best reflection the prediction task at hand. Full details are provided in the sections below.

All models were evaluated using median absolute error, which represents a robust estimate of central tendency that is robust to outliers. We also include median error as a measure of bias, and mean root squared error as alternative error summary statistics. Models were evaluated with respect to total performance in mean years of schooling, as well as other distributional characteristics, such as the proportion with 0 years of schooling. We also assessed the degree to which OOS-PV varied by decade,

region, and type of data held out. In general, the best performing models tended to perform the best across almost all geographies/time periods, so it was not necessary to use multiple models for a single step.

Data Adjustment Model

The data adjustment model serves to correct for bias between different data sources. There are known sources of non-sampling error in educational data series, and we leverage information about these systemic differences to make adjusts to input data. This first requires the definition of “gold standard” data sources, which are presumed to be the most correct data source on average. In almost all cases we used IPUMS or DHS data as the gold standard, given their large samples sizes, transparent methods, and standardized data presentation format. For each region, IPUMS or DHS data were chosen as the gold standard depending on which source had more years of available data. That regional gold standard was subsequently also chosen for each country in the region, to serve as the country-specific gold standard, if there was at least one data point in that country from the data provider. If, for example, IPUMS was considered the regional gold standard, but a country in that region only had DHS data, then DHS would be chosen for the country. A country with both IPUMS and DHS data would follow the regional choice.

The data adjustment model makes corrections for two types of situations. A) when multiple data sources, including gold standard data, are available for the same country, and B) when no gold standard data is available for each country, but we still wish to correct for known underlying biases in the data providers that are available for that country. We therefore employ a model that acts on each of these levels, and consequently, design holdouts to test the predictive validity for each level. The models were compared to a baseline approach, of simply not adjusting the data.

Task 1: No Gold Standard Data Available

In this task, we simulate the total lack of gold standard data for a country, by removed all of the IPUMS or DHS data from one third of the locations for which it is present. We subsequently run the full model and compare—all other variables held constant— how predictive validity varies by model. In effect, the two-thirds of remaining gold standard data was used to adjust all non-gold-standard data, which was then compared to the “held-out” one third of the gold standard data. This had the effect of simulating the instances in which some gold standard data was present in the region, but not present in a country of interest, to see how well regional adjustments could apply across countries. Put another way, we assessed the degree to which biases by data provider were consistent between countries.

This was repeated three times, to minimize the impact of random noise on the OOS-PV statistics. The resulting error statistics were averaged over the three iterations to produce the visualizations below. This task sought to capture the ubiquitous bias by data provider that can be introduced via sampling and survey methodologies. For instance, phone-based surveys such as Eurobarometer, because they only access populations with a connection to a phone, can easily overestimate the educational attainment of a population, and so they should be adjusted downward accordingly.

Task 2: Gold Standard Data Present

In this task, we removed some, but not all, of the gold standard data points for locations in which there were more than two observations of gold standard data. In countries where there is more than one observation, we removed half of the data in order to test how well our data adjustment model performed. This assesses how well the model adjusts for bias within a country that does have gold standard data present. By removing half of the gold standard data, and using the remaining half to adjust non-gold-standard sources, we can assess the reliability of these adjustments, relative to the held-out gold standard data. This was repeated twice, and the resulting error statistics were averaged over the two iterations to produce the final validity statistics.

The models assessed for data adjustment all took the basic form:

$$logit(P_{Q,S,Y,L}) = \beta_0 + \beta_1 * Age + \beta_2 * Location + \beta_3 * Year{:}\,Location + \beta_4 * Sex{:}\,Location + u_{survey\ type} + u_{location:survey\ type}$$

Where $P_{Q,A,S,Y,L}$ is the quantity of interest (either proportion of the population with no education or $\frac{mean\ years\ of\ educational\ attainment}{18}$ for a given age, sex, year, location combination. $u_{survey\ type}$ is a region-specific random effect which captures the average bias between surveys and censuses across all countries within that region and $u_{location:survey\ type}$ is a location-spcific random effect which captures

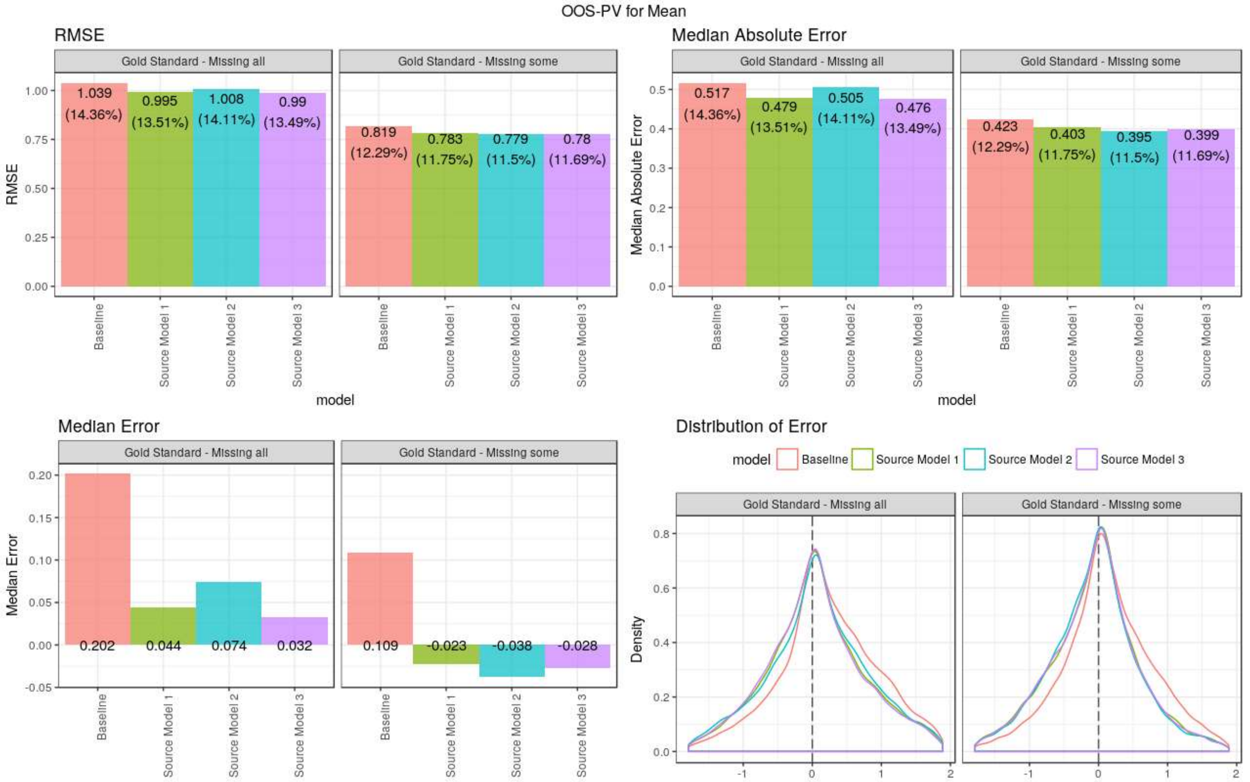

Supplemental Figure 7. OOS-PV Statistics for Source-Type Adjustment Candidate Models

The baseline model, shown in red, represents no data provider adjustment. It is compared to the 3 candidate data source models, as specified above. Predictive validity statistics were calculated for the 'Gold Standard – Missing All' and 'Gold Standard – Missing Some' prediction tasks using n=17,431, and n=16,489 country-year-age-sex-data provider specific error values respectively.

the additional bias between a location-specific gold standard (where applicable) and the other sources present in that location. The above model was run either separately by demographic group where:

- Model 1 was run separately by region and sex,
- Model 2 was run separately by super-region, and
- Model 3 was run separately by region.

Results

All model specifications showed improved performance across all metrics as compared to the baseline mode, representing no adjustment. This was true overall (supplemental figure 7) and when stratified by decade (supplemental figure 8). This validated the decision to perform some kind of data adjustment as opposed to leaving the input data unadjusted. The best performing model across the two tasks was model 3, which was run separately by region, though model 2 did perform slightly better in instances where some gold standard data where available. This result suggests that survey series vary consistently by region, and gender is a less important dimension. While it could be possible that some surveys which only target heads of households or women might have a gender-specific bias to them, that was not observed in a systematic manner in our results. It is important to note that “region” and “super region” are used here as defined in the global burden of disease study(GBD)[32]. In the main text we use GBD super-regions, and refer to them as “regional groupings” for simplicity.

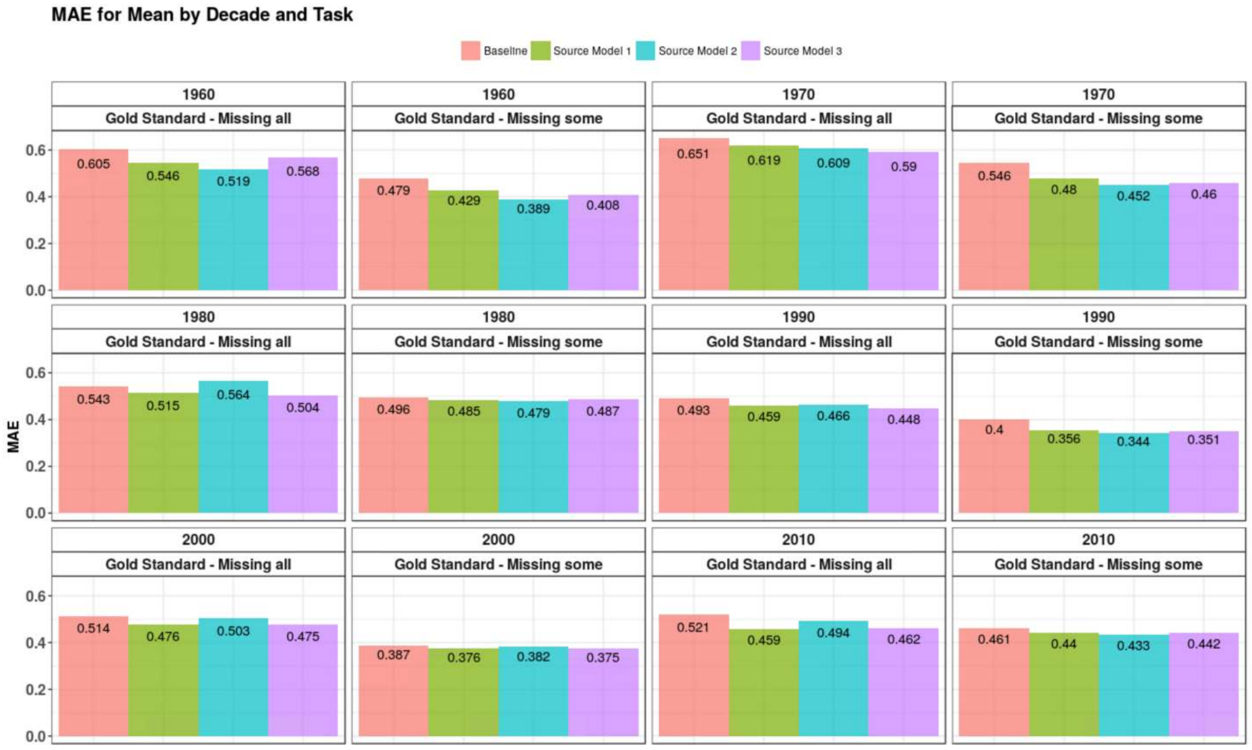

Supplemental Figure 8. OOS-PV for Source-Type Adjustment Candidate Models by Decade and Task

The baseline model, shown in red, represents no data provider adjustment. It is compared to the 3 candidate data source models, as specified above. Predictive validity statistics were calculated for the ‘Gold Standard – Missing All’ and ‘Gold Standard – Missing Some’ prediction tasks using n=17,431, and n=16,489 country-year-age-sex-data provider specific error values respectively.

Cohort Extrapolation

The cohort extrapolation model seeks to leverage the stability of the educational attainment of cohorts over time. This feature of education has been widely used for modeling purposes in numerous analyses[7,13,33]. Within a cohort, education is fairly stable after age 25. For example, the education of 35-year old's in 2000 is likely to be highly similar to the education of 45-year old's in 2010. However, we do wish to capture differential mortality by education over age 65. To test how well our extrapolation model performed, we used data from repeated surveys or censuses repeated at regular intervals. We only used data from within a single data family, e.g. DHS surveys from 2000 and 2005 from the same country. We used an OSS-PV approach, in which we held out repeat observations of the same cohort over time. In a 3-fold knockout scheme, one third of all sources containing repeat observations of a cohort over time were held out. We subsequently ran only the cohort extrapolation model, and the resulting adjusted data were compared against extrapolated data in which no change in education over time was assumed. The resulting error statistics were averaged over the three iterations to produce final predictive validity statistics.

The models considered include:

Model 1 - $y_{Q,L,S,C,A_x} = I + u_{Location:Super\ Region}$

Model 2 - $y_{Q,L,S,C,A_x} = I + u_{Location:Region}$

Model 3 - $y_{Q,L,S,C,A_x} = I + u_{Sex:Location:Super\ Region}$

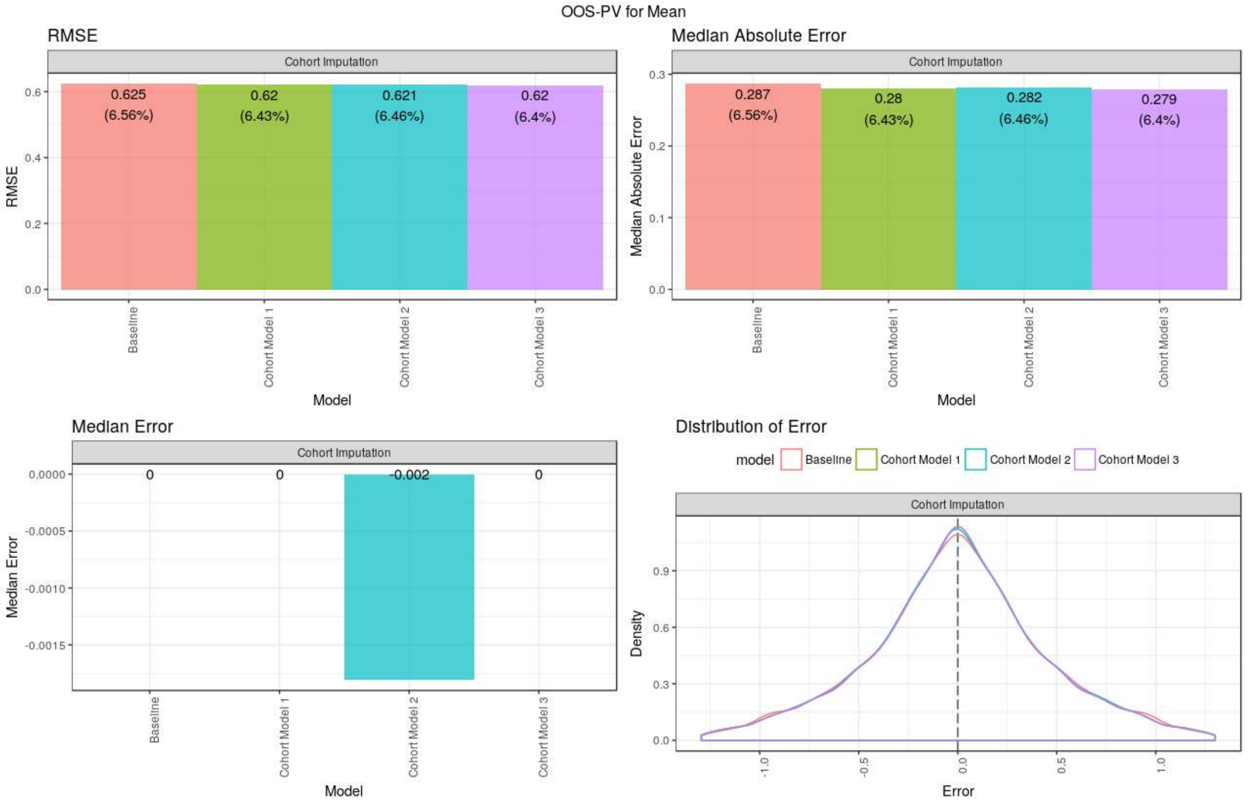

Supplemental Figure 9. OOS-PV for Cohort-Extrapolation Candidate Models

The performance of the three candidate models is shown for predicting within-cohort change in mean educational attainment over time, as compared to a baseline model which assumes no change. For each model, predictive validity statistics were calculated over n=11,126 country-year-cohort-sex-data provider specific error values.

Where *l* is a natural spline with a knot at age 70 intended to capture the potential nonlinearity in the rate of change of differential mortality by education over age and
u is a random intercept at varying levels of geography and sex

Results

All three models showed improvements in OOS-PV over the baseline model which assumed no differential mortality by education (supplemental figure 9). This was also observed almost uniformly when stratified by decade (shown in supplemental figure 10). Nevertheless, improvements in predictive validity were modest. This is likely due to the scarcity of data tracking cohorts between the ages of 65 and 85. Survey series like the DHS often have small sample sizes at older ages due to sampling techniques, causing diminished signal of change in the mean educational attainment of a cohort over time. Furthermore, global coefficients are applied to all cohorts, when the effects of education on preventing early adult mortality may differ by geography. Finally, these results do not account for differential migration by educational attainment. Despite these limitations, this model does show an improvement over the baseline model and has important implications for the final estimates.

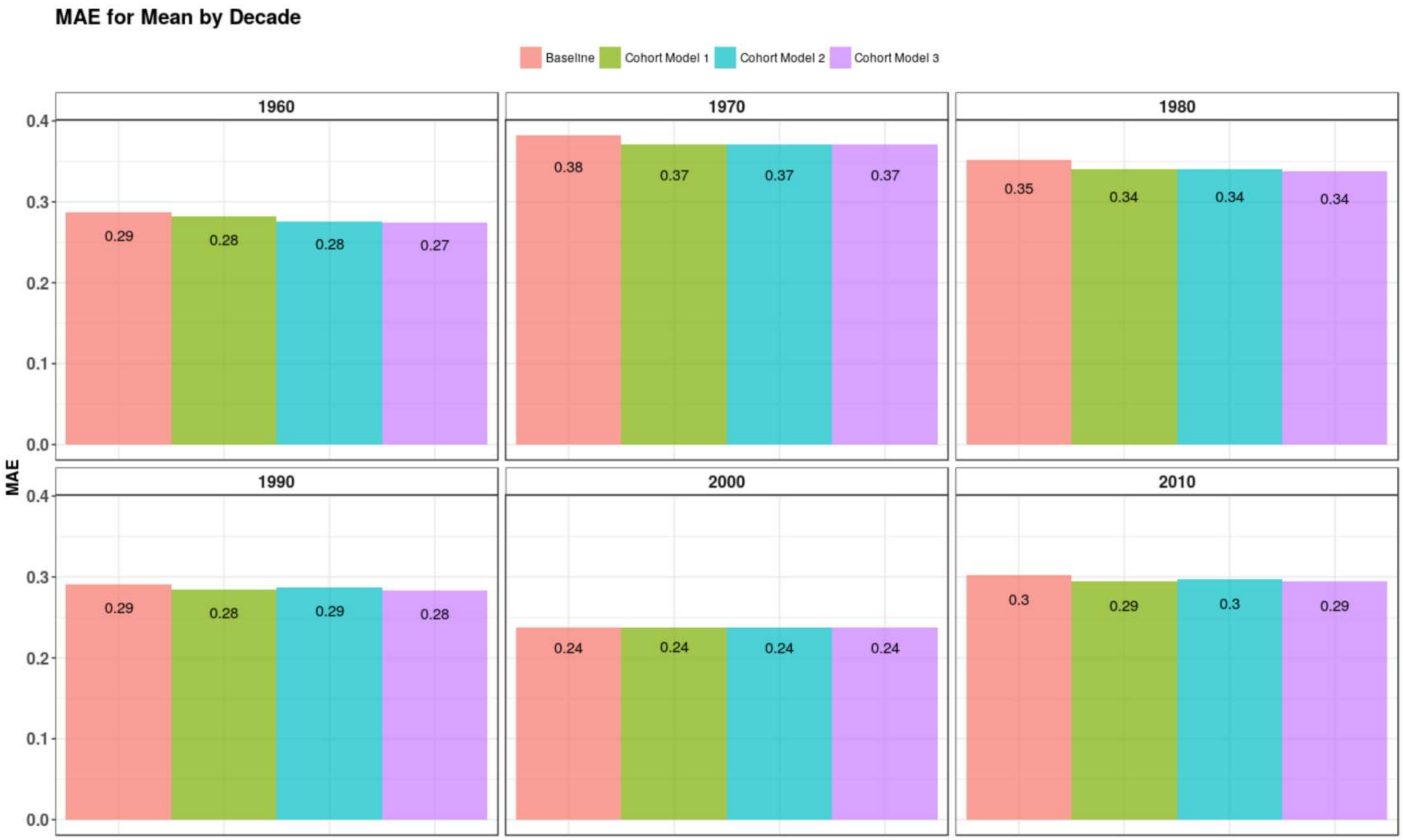

Supplemental Figure 10. OOS-PV for Cohort-Extrapolation Candidate Models by Decade
The performance of the three candidate models is shown for predicting within-cohort change in mean educational attainment over time, as compared to a baseline model which assumes no change. Results are stratified by decade. For each model, predictive validity statistics were calculated over n=11,126 country-year-cohort-sex-data provider specific error values

Ensemble K-Nearest Neighbors Distribution Model

The K Nearest Neighbors Distribution Model has a number of hyper-parameters (as defined in the methods section) which were optimized using OOS-PV. Essentially, we wanted to choose the set of hyper-parameters that would best allow us to predict distributions out of sample. We divided our dataset into two portions, 90% training data and 10% testing data. We fit the model using the training data and compared our results out of sample to the testing data that was held out. This was repeated 3 times to reduce random noise. Only gold standard data, from IPUMS and DHS, were used as testing data in this exercise due to their consistently large sample sizes and reporting data in single-year bins. A “grid search” of hyperparameter values was conducted, meaning that we tested all possible combinations of the below values:

- K: Multiples of 20 between 20 and 200
- Span: Multiples of 0.5 between 0.5 and 3
- Psi: Multiples of 0.5 between 0.5 and 3
- Age Weight (AW): Multiples of 0.05 between 0.1 and 0.9
- Space Weight (SW): Multiples of 0.05 between 0.1 and 0.9
- Cohort Weight (CW): Multiples of 0.05 between 0.1 and 0.9

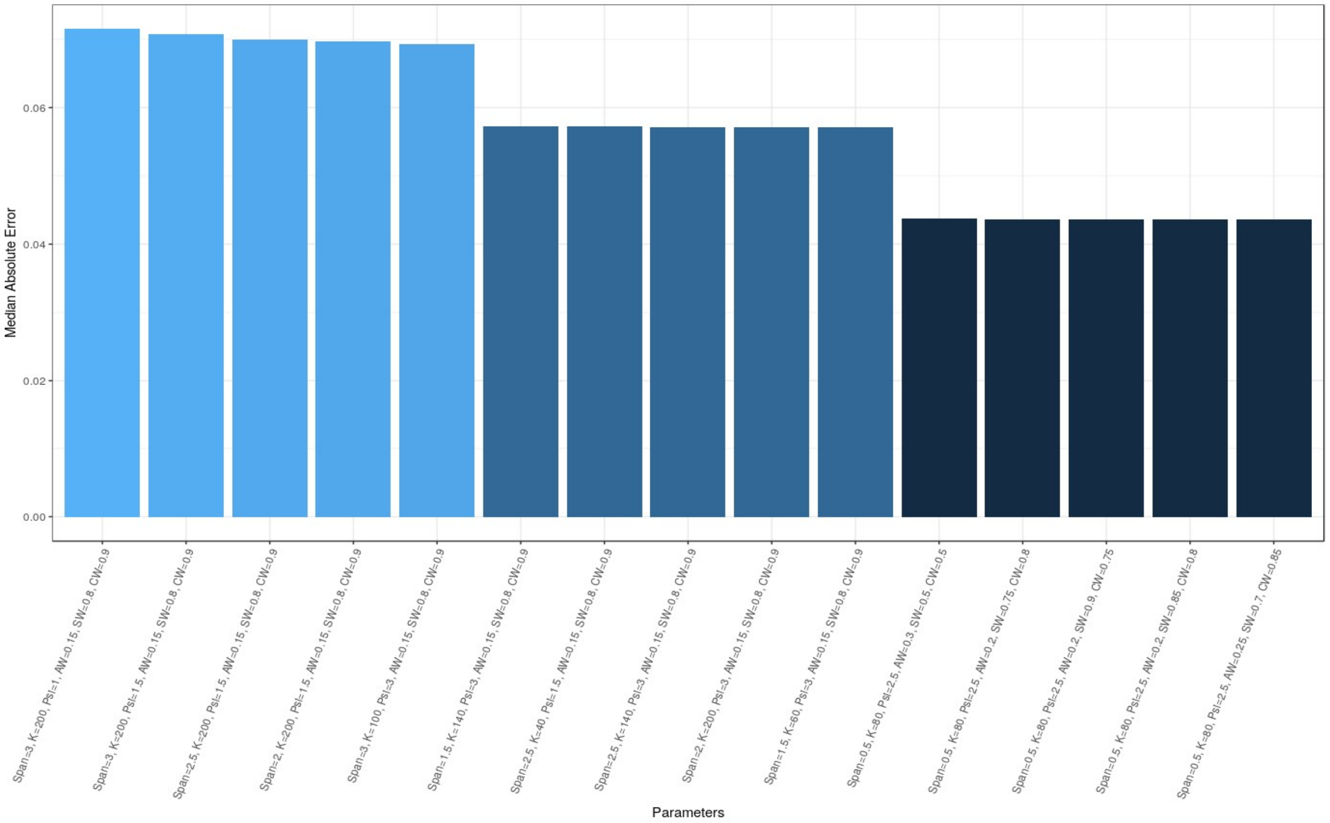

Supplemental Figure 11. OOS-PV for KNN Candidate Hyperparameter Specifications

OOS-PV is shown for the top, middle, and bottom-5 performing combinations of hyperparameters. The median absolute error shown reflects the average of performance in various aspects of the distribution, including: 0 education, 1-6 years, 7-12 years, and 13-18 years. Predictive validity statistics were calculated over n= 14,571 country-year-age-sex-knockout round-bin-specific error values.

Error statistics were combined for each multi-year bin of education attainment, including 0 education, 1-6 years, 7-12 years, and 13-18 years. We assessed predictive validity by taking the average median absolute error for each combination of hyperparameters across each level of education (shown in supplemental figure 11).

Results

The algorithm was relatively robust to the choice of hyperparameter specification. The average median absolute error ranged from 0.045 to 0.072 at the extreme ends of the spectrum. Generally speaking, the algorithm performed best at low levels of smoothing (span), with a medium amount of candidate distributions (K), higher weight on distributions closer in space/time/cohort space (Psi), and with a greater emphasis on cohort and space distance than age distance (CW, SW, and AW). The best performing combination of hyperparameters was the following:

- Span = 0.5
- K = 80
- Psi = 2.5
- Age Weight = 0.25
- Space Weight = 0.7
- Cohort Weight = 0.85
-

Forecasting Model

Finally, we assessed the predictive validity of several approaches to forecasting the distribution of education. In this task, the final 10 years of data were held out, and each forecasting models was used to predict the removed period of the data.

Two model versions, "Forecasting by Means" and "Forecasting by Distributions," were considered for this predictive task. In the former, the modeling framework specified in the main section of this paper was used for all years 1950-2030. That is to say that a linear prior was fit on the data and used with GPR to produce average educational attainment and proportion of the population with no education estimates throughout 2030. Subsequently, the KNN algorithm was run using these predicted inputs as inputs for choosing candidate distributions. This model assumes that a) it is appropriate to forecast mean years of schooling and the proportion with 0 schooling using linear models, and b) it is appropriate to apply past distributions to these projected parameters using the KNN distribution model. In sum, this model involves using the best-performing version of the main model used in the past and running it forward through 2019-2030 to produce forecasts.

The second model uses the distributions of education previously modeled from 1970 through 2018, as described in the methods section, and extrapolates the rate of change of each component of the distribution. We theorized that because mean years of education have not historically changed linearly, using distributions of single-year bins of educational attainment would be a better choice for extrapolation. If proportions increase more linearly in logit space, then we can more accurately predict these proportions and subsequently calculate a more accurate set of mean years of schooling. This was based on the notion that modeling individual component proportions of the distribution would more closely reflect the underlying mechanism of educational development in populations.

We first calculated the rate of change, separately by country and sex, for each multi-year bins of educational attainment (No education, 1-6 years of education, 7-12 years of education, and 13-18 years of education) for the last 10 years of the estimates. We subsequently apply this rate of change to forecast each timeseries through 2030, After this first stage provides estimates of the coarse bins, we employed a similar rate of change model for each single-year bin of educational attainment, to predict the granular distribution over time. We ensured all proportions were internally consistent with the aforementioned predicted multi-year bins, by raking the sum of the single year bins to the total proportion of each larger bin. Subsequently, we calculated the mean years of schooling from these distributions. Finally, we tested the predicted distributions against the 10 years of held-out testing data.

Results

The rate-of-change distribution model had better performance in predicting mean attainment compared to the model that directly forecasts mean attainment. This was evaluated in terms of overall OOS-PV for mean attainment (figure 12). We also assessed performance with respect to other outcome variables, and knockout region and data source (figure 13). This increase in performance is likely due to a more granular, distribution-based model that can better capture the nonlinear growth in mean attainment. While economic development does increase mean statistics linearly for a period, there is an inflection point at which increases in mean educational attainment tend to level off. Using distributions to predict mean educational attainment, is therefore a better choice than using the means themselves for forecasting, as it provides a method of better approximating the mechanisms of educational growth in the population. For example, in many cases the most rapid growth in mean attainment is derived from

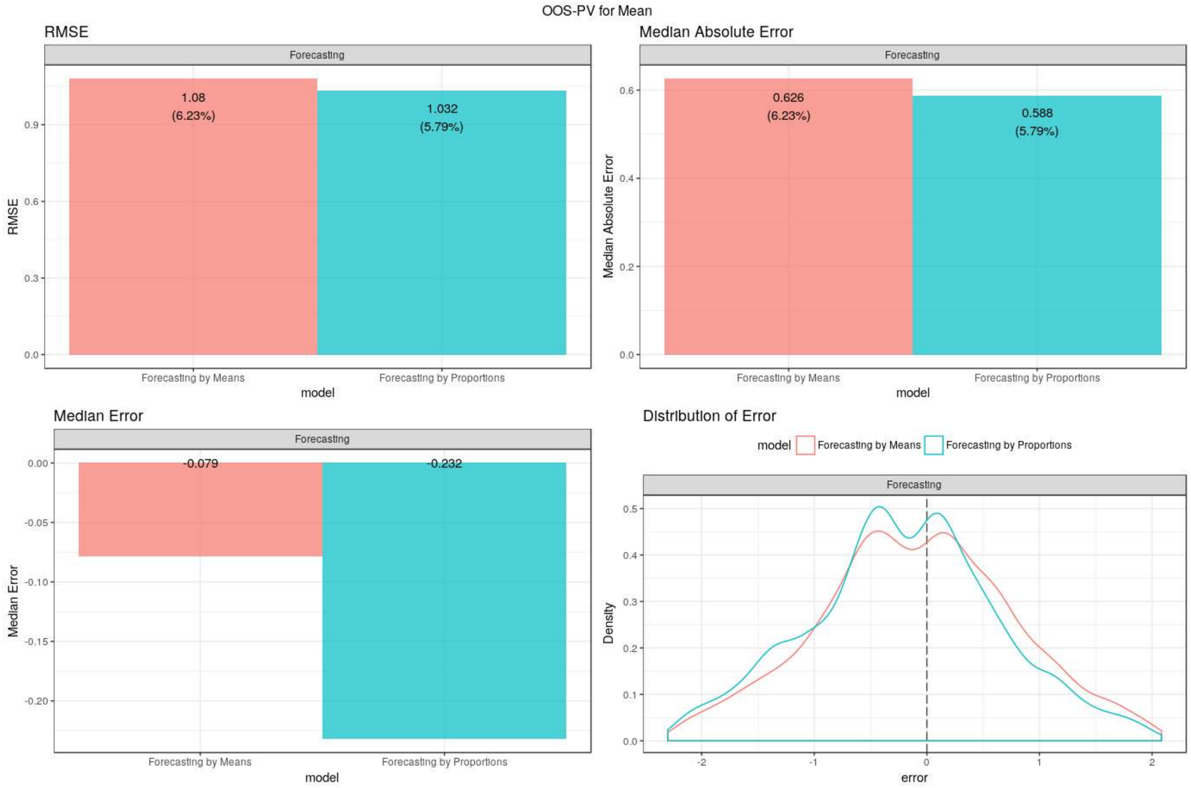

Supplemental Figure 12. OOS-PV for KNN Candidate Forecasting Models

OOS-PV is compared for the two candidate forecasting models. All metrics are shown for mean attainment. Predictive validity statistics were calculated over n=13,627 country-year-age-sex-knockout round specific error values.

reductions in the proportion completing zero years of schooling and increases in primary attainment. Once these changes are complete, then the relatively slower growth in secondary schooling would be the dominant driver in changes, reflected in a gradual leveling off of educational attainment. A completely linear model of mean attainment misses this nuance, whereas a distributional model can better approximate this mechanism. This type of distributional model is likely more reflective of the actual governmental levers and policies that are used to increase educational attainment among populations. For example, the elimination of school fees for a particular level of education, changing the number of years of compulsory schooling, building new schools for a particular level, or increasing the number of years required to complete a certain level, are all mechanisms that might create linear expansion in a specific level of schooling.

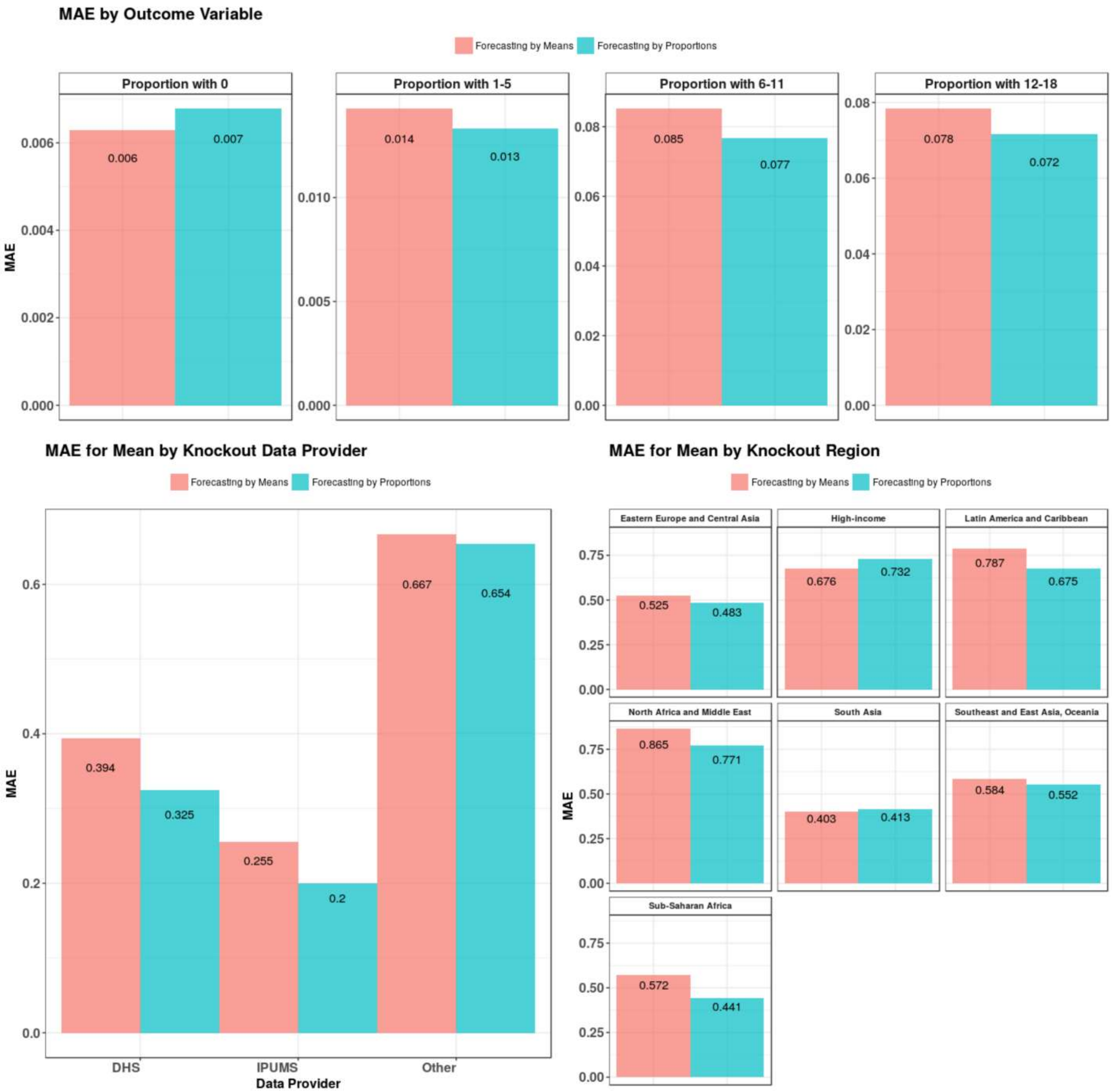

Supplemental Figure 13. Other Dimensions of OOS-PV Considered for Forecasting Models

Median absolute error (MAE) is shown by outcome variable, data provider, and knockout region to illustrate other dimensions of variation that were considered in selecting a forecasting model. Predictive validity statistics were calculated over n=13,627 country-year-age-sex-knockout round specific error values.

Supplemental References

1. Tempkin, L. *Inequality*. (Oxford University Press, 1993).
2. Thomas, V., Wang, Y. & Fan, X. *Measuring education inequality: Gini coefficients of education*. vol. 2525 (World Bank Publications, 2001).
3. Wail, B., Hanchane, S. & Kamal, A. A New Data Set of Educational Inequality in the World, 1950-2010: Gini Index of Education by Age Group. *SSRN Journal* (2011) doi:10.2139/ssrn.1895496.
4. Ziesemer, T. *Gini coefficients of education for 146 countries, 1950-2010*. https://ideas.repec.org/p/unm/unumer/2016044.html (2016).
5. Gakidou, E. Health Inequality: Definition, Measurement, and Determinants. (Harvard Unviersity, 2001).
6. Gakidou, E. & King, G. Measuring total health inequality: adding individual variation to group-level differences. *Int J Equity Health* **1**, 3 (2002).
7. Barro, R. J. & Lee, J. W. A new data set of educational attainment in the world, 1950–2010. *Journal of Development Economics* **104**, 184–198 (2013).
8. Barro, R. J. & Lee, J.-W. *Educational Attainment—Quantity and Quality of Schooling*. (Oxford University Press, 2015).
9. Barro, R. J. & Lee, J. W. International Measures of Schooling Years and Schooling Quality. *The American Economic Review* **86**, 218–223 (1996).
10. Barro, R. J. & Lee, J.-W. International comparisons of educational attainment. *Journal of monetary economics* **32**, 363–394 (1993).
11. International Standard Classification of Education (ISCED). *UNESCO* http://uis.unesco.org/en/topic/international-standard-classification-education-isced (2017).
12. Friedman, J., Graetz, N. & Gakidou, E. Improving the estimation of educational attainment: new methods for assessing average years of schooling from binned data. *PLOS ONE* (2018).

13. Kc, S., Barakat, B., Goujon, A., Skirbekk, V. & Lutz, W. Projection of populations by level of educational attainment, age, and sex for 120 countries for 2005-2050. *Demographic Research* **22**, 383–472 (2010).

14. Goujon, A. *et al.* A HARMONIZED DATASET ON GLOBAL EDUCATIONAL ATTAINMENT BETWEEN 1970 AND 2060 – AN ANALYTICAL WINDOW INTO RECENT TRENDS AND FUTURE PROSPECTS IN HUMAN CAPITAL DEVELOPMENT. *Journal of Demographic Economics* **82**, 315–363 (2016).

15. Lutz, W., Goujon, A., Kc, S., Stonawski, M. & Stilianakis, N. Demographic and human capital scenarios for the 21st century. 598.

16. Nilsson, M., Griggs, D. & Visbeck, M. Policy: Map the interactions between Sustainable Development Goals. *Nature* **534**, 320 (2016).

17. Battistin, E., De Nadai, M. & Sianesi, B. Misreported schooling, multiple measures and returns to educational qualifications. *Journal of Econometrics* **181**, 136–150 (2014).

18. Hu, Y., Xiao, R. & Zhong, X. Misclassification of Schooling in Survey and Transcript Samples. 19.

19. Barro-Lee.com. http://www.barrolee.com/.

20. Wittgenstein Centre Human Capital Data Explorer. http://dataexplorer.wittgensteincentre.org/wcde-v2/.

21. Unesco. Migration, displacement and education: building bridges, not walls. https://en.unesco.org/gem-report/report/2019/migration (2018).

22. Lim, S. S. *et al.* Measuring human capital: a systematic analysis of 195 countries and territories, 1990–2016. *The Lancet* (2018) doi:10.1016/S0140-6736(18)31941-X.

23. Filmer, D., Rogers, H., Angrist, N. & Sabarwal, S. *Learning-Adjusted Years of Schooling (LAYS): Defining a New Macro Measure of Education*. (The World Bank, 2018). doi:10.1596/1813-9450-8591.

24. Kraay, A. *Methodology for a World Bank Human Capital Index*. (The World Bank, 2018). doi:10.1596/1813-9450-8593.

25. World Development Report 2019: The Changing Nature of Work. *World Bank* https://www.worldbank.org/en/publication/wdr2019.

26. Kumar, S. Influence of open educational resources on educational practices in the Global South. *Nature Human Behaviour* 1 (2019) doi:10.1038/s41562-019-0624-4.

27. King, G. 'Truth' Is Stranger than Prediction, More Questionable than Causal Inference. *American Journal of Political Science* **35**, 1047–1053 (1991).

28. Hoeting, J. A., Madigan, D., Raftery, A. E. & Volinsky, C. T. Bayesian model averaging: a tutorial (with comments by M. Clyde, David Draper and E. I. George, and a rejoinder by the authors. *Statistical Science* **14**, 382–417 (1999).

29. Stone, M. Cross-validatory choice and assessment of statistical predictions. *Journal of the Royal Statistical Society. Series B (Methodological)* 111–147 (1974).

30. Power, M. Theoretical Modelling AspectsThe predictive validation of ecological and environmental models. *Ecological Modelling* **68**, 33–50 (1993).

31. Tashman, L. J. Out-of-sample tests of forecasting accuracy: an analysis and review. *International Journal of Forecasting* **16**, 437–450 (2000).

32. Dicker, D. *et al.* Global, regional, and national age-sex-specific mortality and life expectancy, 1950–2017: a systematic analysis for the Global Burden of Disease Study 2017. *The Lancet* **392**, 1684–1735 (2018).

33. Gakidou, E., Cowling, K., Lozano, R. & Murray, C. J. Increased educational attainment and its effect on child mortality in 175 countries between 1970 and 2009: a systematic analysis. *The Lancet* **376**, 959–974 (2010).

Supplemental Tables and Figures

Country	Year	Mean Male	Mean Female	Gender Gap	AID	Mean	Prop 0	Prop 6+	Prop 12+	Prop 15+
**Afghanistan**	1970	1.27	0.25	-1.01	1.40	0.76	0.89	0.07	0.02	0.01
**Afghanistan**	2018	7.06	2.44	-4.62	6.29	4.75	0.55	0.39	0.25	0.10
**Afghanistan**	2030	11.74	5.38	-6.37	7.28	8.56	0.25	0.64	0.51	0.22
**Albania**	1970	8.50	6.49	-2.01	4.51	7.49	0.05	0.64	0.22	0.06
**Albania**	2018	10.57	11.06	0.50	3.39	10.81	0.01	0.97	0.49	0.11
**Albania**	2030	10.84	11.70	0.86	3.35	11.27	0.01	0.98	0.55	0.13
**Algeria**	1970	1.99	0.69	-1.30	2.20	1.34	0.73	0.09	0.02	0.01
**Algeria**	2018	10.27	9.72	-0.55	6.19	10.00	0.16	0.79	0.52	0.23
**Algeria**	2030	12.51	13.02	0.51	4.43	12.77	0.04	0.94	0.72	0.38
**American Samoa**	1970	9.27	8.83	-0.45	5.33	9.05	0.09	0.80	0.35	0.13
**American Samoa**	2018	13.00	13.66	0.66	3.33	13.33	0.02	0.98	0.84	0.35
**American Samoa**	2030	13.69	14.48	0.79	3.14	14.09	0.01	0.99	0.89	0.45
**Andorra**	1970	11.36	10.66	-0.70	4.44	11.01	0.03	0.91	0.53	0.19
**Andorra**	2018	13.92	14.18	0.27	3.50	14.05	0.02	0.98	0.84	0.49
**Andorra**	2030	14.30	14.69	0.39	3.30	14.50	0.01	0.98	0.87	0.57
**Angola**	1970	2.39	0.58	-1.81	2.35	1.48	0.69	0.10	0.02	0.01
**Angola**	2018	10.24	7.60	-2.63	5.58	8.92	0.11	0.77	0.35	0.14
**Angola**	2030	11.67	10.05	-1.62	4.63	10.86	0.02	0.91	0.49	0.20
**Antigua and Barbuda**	1970	7.87	7.33	-0.54	4.89	7.60	0.07	0.71	0.19	0.08
**Antigua and Barbuda**	2018	12.25	12.86	0.61	3.19	12.56	0.01	0.98	0.73	0.24
**Antigua and Barbuda**	2030	12.78	13.51	0.73	2.83	13.14	0.00	0.99	0.82	0.28
**Argentina**	1970	7.82	7.55	-0.27	4.58	7.69	0.03	0.76	0.26	0.09
**Argentina**	2018	11.59	12.03	0.44	3.68	11.81	0.00	0.97	0.70	0.26
**Argentina**	2030	11.94	12.48	0.54	3.25	12.21	0.00	0.99	0.75	0.27
**Armenia**	1970	11.06	10.65	-0.41	3.26	10.86	0.01	0.96	0.40	0.14
**Armenia**	2018	12.36	13.24	0.89	2.78	12.80	0.00	0.99	0.74	0.30
**Armenia**	2030	12.58	13.71	1.13	2.83	13.15	0.00	0.99	0.77	0.35
**Australia**	1970	9.49	8.69	-0.80	5.12	9.09	0.08	0.81	0.35	0.12
**Australia**	2018	13.23	13.81	0.59	3.15	13.52	0.01	0.98	0.85	0.36
**Australia**	2030	13.77	14.67	0.90	2.93	14.22	0.01	0.99	0.91	0.46
**Austria**	1970	11.12	10.01	-1.12	2.98	10.56	0.00	0.99	0.39	0.07
**Austria**	2018	12.89	13.10	0.21	2.35	13.00	0.00	0.99	0.84	0.20
**Austria**	2030	13.07	13.76	0.69	2.42	13.41	0.00	0.99	0.89	0.25

**Azerbaijan**	1970	10.85	8.72	-2.13	4.32	9.78	0.05	0.86	0.29	0.12
**Azerbaijan**	2018	12.43	12.36	-0.07	2.96	12.40	0.00	0.99	0.61	0.22
**Azerbaijan**	2030	12.93	12.70	-0.23	3.04	12.81	0.00	0.99	0.68	0.26
**Bahrain**	1970	3.82	1.51	-2.31	3.92	2.66	0.60	0.24	0.07	0.03
**Bahrain**	2018	11.29	10.59	-0.70	6.30	10.94	0.16	0.82	0.63	0.30
**Bahrain**	2030	13.83	12.73	-1.10	4.70	13.28	0.06	0.94	0.80	0.44
**Bangladesh**	1970	3.68	1.12	-2.55	3.53	2.40	0.62	0.17	0.04	0.01
**Bangladesh**	2018	7.58	7.00	-0.57	5.48	7.29	0.16	0.63	0.22	0.09
**Bangladesh**	2030	9.55	9.76	0.21	5.10	9.66	0.04	0.82	0.38	0.19
**Barbados**	1970	8.54	8.19	-0.35	4.17	8.36	0.01	0.81	0.20	0.07
**Barbados**	2018	13.10	13.66	0.57	2.89	13.38	0.00	0.99	0.84	0.32
**Barbados**	2030	13.67	14.33	0.66	2.89	14.00	0.00	0.99	0.90	0.42
**Belarus**	1970	10.36	10.10	-0.27	4.12	10.23	0.02	0.88	0.34	0.12
**Belarus**	2018	13.49	14.32	0.82	2.86	13.91	0.00	0.99	0.88	0.38
**Belarus**	2030	14.09	14.77	0.68	2.77	14.43	0.00	0.99	0.92	0.47
**Belgium**	1970	10.35	9.42	-0.93	5.21	9.88	0.07	0.90	0.40	0.20
**Belgium**	2018	13.63	14.17	0.54	3.15	13.90	0.01	0.99	0.85	0.46
**Belgium**	2030	13.86	14.64	0.78	3.00	14.25	0.01	0.99	0.88	0.52
**Belize**	1970	5.37	5.09	-0.28	4.91	5.23	0.22	0.46	0.10	0.05
**Belize**	2018	9.54	9.75	0.21	4.54	9.65	0.03	0.87	0.35	0.13
**Belize**	2030	10.53	10.80	0.27	3.93	10.67	0.01	0.94	0.42	0.15
**Benin**	1970	1.48	0.43	-1.05	1.72	0.96	0.86	0.08	0.02	0.01
**Benin**	2018	6.51	3.42	-3.09	5.70	4.96	0.42	0.44	0.16	0.05
**Benin**	2030	9.11	6.01	-3.11	6.00	7.56	0.20	0.66	0.32	0.10
**Bermuda**	1970	6.62	7.40	0.78	6.15	7.01	0.27	0.62	0.26	0.10
**Bermuda**	2018	11.41	12.79	1.38	4.84	12.10	0.07	0.91	0.66	0.32
**Bermuda**	2030	12.13	13.43	1.30	4.28	12.78	0.05	0.94	0.72	0.36
**Bhutan**	1970	0.49	0.19	-0.30	0.65	0.34	0.95	0.03	0.01	0.00
**Bhutan**	2018	5.09	3.97	-1.12	5.35	4.53	0.43	0.37	0.12	0.05
**Bhutan**	2030	7.22	8.00	0.79	5.82	7.61	0.15	0.63	0.27	0.12
**Bolivia**	1970	5.77	3.80	-1.97	5.11	4.78	0.27	0.38	0.12	0.06
**Bolivia**	2018	10.70	9.78	-0.92	4.67	10.24	0.03	0.86	0.45	0.16
**Bolivia**	2030	11.39	10.82	-0.57	3.90	11.10	0.01	0.93	0.52	0.16
**Bosnia and Herzegovina**	1970	8.94	5.41	-3.52	5.15	7.17	0.13	0.62	0.19	0.06
**Bosnia and Herzegovina**	2018	12.05	12.07	0.02	2.94	12.06	0.01	0.98	0.66	0.16
**Bosnia and Herzegovina**	2030	12.34	12.67	0.33	2.98	12.51	0.00	0.99	0.71	0.20
**Botswana**	1970	2.80	2.51	-0.29	3.65	2.66	0.52	0.21	0.04	0.01
**Botswana**	2018	9.60	9.83	0.23	4.08	9.72	0.04	0.88	0.37	0.09
**Botswana**	2030	10.65	10.75	0.10	3.24	10.70	0.01	0.95	0.46	0.10
**Brazil**	1970	4.03	3.81	-0.23	4.38	3.92	0.30	0.24	0.06	0.04
**Brazil**	2018	9.72	10.34	0.62	5.13	10.03	0.03	0.81	0.36	0.21
**Brazil**	2030	11.28	11.82	0.53	4.53	11.55	0.01	0.91	0.53	0.28
**Brunei**	1970	6.54	3.35	-3.19	5.28	4.94	0.34	0.45	0.12	0.05

**Brunei**	2018	12.27	11.65	-0.62	4.97	11.96	0.07	0.91	0.64	0.30
**Brunei**	2030	13.05	12.78	-0.28	4.34	12.91	0.04	0.95	0.70	0.35
**Bulgaria**	1970	11.29	10.92	-0.37	3.75	11.10	0.02	0.94	0.50	0.16
**Bulgaria**	2018	12.57	13.36	0.78	3.12	12.96	0.00	0.99	0.72	0.32
**Bulgaria**	2030	12.84	13.89	1.05	3.05	13.36	0.00	0.99	0.79	0.34
**Burkina Faso**	1970	0.42	0.20	-0.22	0.60	0.31	0.96	0.03	0.01	0.00
**Burkina Faso**	2018	3.04	1.53	-1.52	3.50	2.28	0.65	0.19	0.05	0.02
**Burkina Faso**	2030	4.95	2.66	-2.29	4.63	3.81	0.41	0.30	0.08	0.03
**Burundi**	1970	1.40	0.63	-0.77	1.72	1.02	0.78	0.06	0.01	0.00
**Burundi**	2018	5.80	4.36	-1.44	5.08	5.08	0.29	0.45	0.11	0.04
**Burundi**	2030	8.15	7.04	-1.11	4.91	7.60	0.07	0.71	0.20	0.07
**Cambodia**	1970	3.44	1.65	-1.80	3.20	2.55	0.45	0.18	0.02	0.01
**Cambodia**	2018	7.10	5.89	-1.20	4.46	6.49	0.08	0.60	0.15	0.04
**Cambodia**	2030	8.44	7.49	-0.95	4.36	7.97	0.03	0.77	0.23	0.07
**Cameroon**	1970	3.60	1.14	-2.46	3.45	2.37	0.61	0.20	0.03	0.01
**Cameroon**	2018	9.78	8.23	-1.55	5.59	9.01	0.12	0.78	0.37	0.14
**Cameroon**	2030	11.28	10.55	-0.73	4.79	10.92	0.04	0.90	0.55	0.23
**Canada**	1970	11.90	11.63	-0.27	3.53	11.77	0.01	0.95	0.68	0.16
**Canada**	2018	13.95	14.25	0.30	2.93	14.10	0.01	0.99	0.91	0.44
**Canada**	2030	14.26	14.66	0.41	3.04	14.46	0.01	0.98	0.91	0.52
**Cape Verde**	1970	1.49	0.73	-0.76	2.01	1.11	0.86	0.09	0.03	0.02
**Cape Verde**	2018	7.31	7.49	0.18	5.73	7.40	0.19	0.66	0.25	0.09
**Cape Verde**	2030	9.57	9.92	0.35	4.89	9.75	0.04	0.85	0.40	0.16
**Central African Republic**	1970	1.65	0.31	-1.34	1.67	0.98	0.77	0.06	0.01	0.00
**Central African Republic**	2018	7.67	4.45	-3.22	5.42	6.06	0.21	0.53	0.16	0.06
**Central African Republic**	2030	9.62	6.89	-2.74	5.22	8.25	0.06	0.73	0.27	0.11
**Chad**	1970	0.77	0.23	-0.54	0.95	0.50	0.91	0.04	0.01	0.00
**Chad**	2018	5.08	1.97	-3.10	4.70	3.53	0.51	0.30	0.09	0.03
**Chad**	2030	7.51	3.76	-3.75	5.67	5.64	0.27	0.48	0.18	0.06
**Chile**	1970	7.60	6.76	-0.84	5.19	7.18	0.12	0.66	0.21	0.07
**Chile**	2018	12.72	12.50	-0.22	3.02	12.61	0.01	0.98	0.76	0.23
**Chile**	2030	13.19	13.10	-0.08	2.71	13.14	0.00	0.99	0.82	0.27
**China**	1970	5.70	3.50	-2.20	4.33	4.60	0.28	0.47	0.06	0.02
**China**	2018	11.45	12.32	0.87	3.86	11.88	0.01	0.97	0.60	0.25
**China**	2030	13.27	14.01	0.74	3.27	13.64	0.00	0.99	0.80	0.45
**Colombia**	1970	4.73	4.27	-0.46	4.33	4.50	0.20	0.34	0.07	0.04
**Colombia**	2018	10.57	10.47	-0.10	4.42	10.52	0.02	0.91	0.45	0.17
**Colombia**	2030	11.57	11.40	-0.16	3.67	11.48	0.01	0.97	0.58	0.19
**Comoros**	1970	1.07	0.34	-0.74	1.31	0.71	0.88	0.06	0.02	0.01
**Comoros**	2018	9.64	8.12	-1.52	6.33	8.88	0.17	0.74	0.40	0.18
**Comoros**	2030	11.17	10.83	-0.34	5.47	11.00	0.06	0.87	0.54	0.27
**Congo**	1970	5.37	1.39	-3.98	4.38	3.38	0.49	0.29	0.06	0.02
**Congo**	2018	11.72	11.30	-0.42	4.04	11.51	0.03	0.94	0.60	0.20

**Congo**	2030	12.76	12.78	0.03	3.51	12.77	0.01	0.98	0.73	0.28
**Costa Rica**	1970	6.74	6.04	-0.70	4.98	6.39	0.10	0.60	0.14	0.08
**Costa Rica**	2018	10.75	10.79	0.04	4.30	10.77	0.02	0.93	0.47	0.19
**Costa Rica**	2030	11.22	11.32	0.10	3.69	11.27	0.01	0.96	0.54	0.17
**Cote d'Ivoire**	1970	1.68	0.42	-1.26	1.88	1.05	0.84	0.09	0.02	0.01
**Cote d'Ivoire**	2018	7.01	4.45	-2.55	6.03	5.73	0.36	0.51	0.21	0.07
**Cote d'Ivoire**	2030	9.07	7.08	-1.99	5.99	8.08	0.17	0.70	0.36	0.11
**Croatia**	1970	10.06	8.20	-1.85	4.41	9.13	0.04	0.81	0.30	0.10
**Croatia**	2018	12.58	13.34	0.75	2.69	12.96	0.00	0.99	0.78	0.25
**Croatia**	2030	12.76	13.73	0.97	2.79	13.24	0.00	0.99	0.76	0.30
**Cuba**	1970	7.60	6.88	-0.73	5.66	7.24	0.19	0.67	0.23	0.08
**Cuba**	2018	12.28	13.05	0.77	3.57	12.67	0.01	0.99	0.72	0.23
**Cuba**	2030	12.78	13.83	1.05	3.28	13.30	0.00	0.99	0.80	0.27
**Cyprus**	1970	9.15	7.16	-1.99	5.42	8.16	0.10	0.70	0.31	0.11
**Cyprus**	2018	13.51	14.16	0.65	3.31	13.84	0.01	0.98	0.86	0.46
**Cyprus**	2030	13.81	14.96	1.15	3.20	14.38	0.01	0.99	0.87	0.56
**Czech Republic**	1970	11.78	11.29	-0.50	2.75	11.53	0.01	0.98	0.57	0.10
**Czech Republic**	2018	13.02	13.58	0.56	2.24	13.30	0.00	0.99	0.91	0.22
**Czech Republic**	2030	12.73	14.34	1.61	2.82	13.54	0.00	0.97	0.93	0.30
**Democratic Republic of the Congo**	1970	5.63	1.41	-4.22	4.38	3.52	0.44	0.28	0.06	0.02
**Democratic Republic of the Congo**	2018	11.58	9.62	-1.96	4.90	10.60	0.06	0.88	0.50	0.19
**Democratic Republic of the Congo**	2030	13.06	11.91	-1.15	3.89	12.48	0.01	0.97	0.69	0.28
**Denmark**	1970	12.85	12.26	-0.59	3.80	12.56	0.02	0.97	0.71	0.28
**Denmark**	2018	14.62	14.99	0.36	2.49	14.81	0.00	0.99	0.90	0.63
**Denmark**	2030	15.15	15.43	0.27	2.50	15.29	0.01	0.99	0.92	0.72
**Djibouti**	1970	1.01	0.32	-0.69	1.23	0.66	0.88	0.05	0.02	0.01
**Djibouti**	2018	8.89	5.51	-3.38	6.52	7.20	0.26	0.60	0.30	0.12
**Djibouti**	2030	11.16	8.81	-2.35	5.88	9.99	0.06	0.80	0.46	0.22
**Dominica**	1970	9.23	9.67	0.44	5.03	9.45	0.07	0.83	0.36	0.13
**Dominica**	2018	13.66	14.31	0.65	3.26	13.99	0.01	0.98	0.88	0.46
**Dominica**	2030	14.26	14.97	0.72	2.95	14.62	0.01	0.99	0.92	0.55
**Dominican Republic**	1970	4.42	3.82	-0.61	4.31	4.12	0.26	0.31	0.07	0.03
**Dominican Republic**	2018	9.57	10.33	0.76	4.95	9.95	0.04	0.84	0.44	0.16
**Dominican Republic**	2030	10.43	11.27	0.84	4.40	10.85	0.02	0.90	0.52	0.18
**Ecuador**	1970	6.07	5.37	-0.70	5.06	5.72	0.17	0.51	0.13	0.07
**Ecuador**	2018	11.72	11.75	0.02	3.65	11.73	0.02	0.96	0.61	0.19
**Ecuador**	2030	11.92	12.03	0.12	3.00	11.98	0.00	0.98	0.61	0.16
**Egypt**	1970	5.11	2.05	-3.06	5.07	3.58	0.58	0.30	0.14	0.06
**Egypt**	2018	12.74	11.06	-1.68	5.68	11.90	0.12	0.87	0.68	0.35
**Egypt**	2030	14.01	13.66	-0.35	4.21	13.83	0.04	0.95	0.81	0.46
**El Salvador**	1970	3.91	3.04	-0.87	4.13	3.47	0.37	0.30	0.06	0.03
**El Salvador**	2018	8.81	8.50	-0.31	5.35	8.65	0.08	0.77	0.31	0.13
**El Salvador**	2030	9.74	9.79	0.04	4.79	9.77	0.04	0.87	0.38	0.15

**Equatorial Guinea**	1970	5.74	1.80	-3.95	4.32	3.77	0.36	0.30	0.06	0.02
**Equatorial Guinea**	2018	13.66	12.80	-0.86	3.54	13.23	0.02	0.97	0.80	0.36
**Equatorial Guinea**	2030	14.57	13.92	-0.65	2.93	14.24	0.00	0.99	0.90	0.45
**Eritrea**	1970	1.25	0.36	-0.89	1.45	0.80	0.85	0.06	0.01	0.00
**Eritrea**	2018	8.11	4.64	-3.47	5.72	6.37	0.23	0.56	0.20	0.07
**Eritrea**	2030	10.43	7.06	-3.37	5.56	8.74	0.06	0.75	0.34	0.14
**Estonia**	1970	12.05	12.42	0.37	3.06	12.23	0.00	0.99	0.64	0.24
**Estonia**	2018	13.48	14.36	0.88	3.13	13.92	0.00	0.99	0.89	0.41
**Estonia**	2030	13.92	15.44	1.51	3.25	14.68	0.00	0.99	0.92	0.50
**Ethiopia**	1970	0.75	0.20	-0.55	0.90	0.47	0.92	0.03	0.01	0.00
**Ethiopia**	2018	5.89	3.09	-2.80	5.13	4.49	0.38	0.39	0.11	0.04
**Ethiopia**	2030	8.08	5.56	-2.53	5.44	6.82	0.15	0.60	0.18	0.08
**Federated States of Micronesia**	1970	7.76	4.64	-3.11	5.88	6.20	0.27	0.56	0.21	0.08
**Federated States of Micronesia**	2018	11.21	10.94	-0.27	4.44	11.07	0.03	0.92	0.54	0.19
**Federated States of Micronesia**	2030	11.95	11.99	0.04	3.88	11.97	0.01	0.96	0.63	0.22
**Fiji**	1970	6.79	5.86	-0.93	3.98	6.33	0.08	0.64	0.10	0.04
**Fiji**	2018	12.11	12.59	0.49	3.09	12.35	0.00	0.99	0.69	0.21
**Fiji**	2030	12.90	13.53	0.63	2.98	13.22	0.00	0.99	0.80	0.31
**Finland**	1970	10.65	10.69	0.04	4.35	10.67	0.02	0.92	0.51	0.17
**Finland**	2018	13.69	14.57	0.88	3.20	14.13	0.00	0.99	0.86	0.52
**Finland**	2030	14.34	15.16	0.82	3.19	14.75	0.00	0.99	0.88	0.61
**France**	1970	9.60	9.14	-0.46	4.94	9.37	0.04	0.67	0.39	0.12
**France**	2018	13.52	13.97	0.45	3.26	13.74	0.01	0.99	0.81	0.42
**France**	2030	14.15	14.86	0.71	3.19	14.50	0.00	0.99	0.87	0.54
**Gabon**	1970	4.54	1.67	-2.86	4.06	3.10	0.50	0.25	0.05	0.02
**Gabon**	2018	11.68	11.82	0.15	4.42	11.75	0.04	0.93	0.62	0.25
**Gabon**	2030	12.99	13.18	0.18	3.72	13.08	0.01	0.97	0.76	0.33
**Georgia**	1970	11.71	11.83	0.12	4.23	11.77	0.04	0.94	0.60	0.24
**Georgia**	2018	13.40	14.04	0.64	3.06	13.72	0.01	0.99	0.86	0.40
**Georgia**	2030	13.73	15.06	1.33	3.18	14.39	0.01	0.99	0.90	0.48
**Germany**	1970	13.48	12.68	-0.81	3.07	13.08	0.00	0.99	0.64	0.29
**Germany**	2018	14.56	14.69	0.13	2.98	14.63	0.01	0.99	0.91	0.56
**Germany**	2030	14.50	14.76	0.26	3.13	14.63	0.01	0.98	0.88	0.65
**Ghana**	1970	5.80	2.19	-3.61	5.40	4.00	0.56	0.36	0.12	0.06
**Ghana**	2018	11.10	9.44	-1.65	6.02	10.27	0.14	0.81	0.53	0.26
**Ghana**	2030	12.90	11.95	-0.95	4.45	12.42	0.04	0.94	0.67	0.37
**Greece**	1970	8.44	7.01	-1.43	4.20	7.72	0.03	0.81	0.23	0.08
**Greece**	2018	13.00	13.42	0.41	3.26	13.21	0.00	0.99	0.83	0.36
**Greece**	2030	13.42	14.13	0.71	3.09	13.78	0.00	0.99	0.86	0.43
**Greenland**	1970	7.98	7.89	-0.09	4.03	7.93	0.02	0.77	0.18	0.06
**Greenland**	2018	10.20	10.63	0.43	3.59	10.41	0.01	0.96	0.44	0.11
**Greenland**	2030	10.64	11.14	0.51	3.46	10.89	0.01	0.97	0.50	0.13
**Grenada**	1970	5.44	5.31	-0.13	3.36	5.38	0.03	0.51	0.07	0.02

**Grenada**	2018	10.52	11.02	0.50	3.59	10.77	0.01	0.95	0.43	0.12
**Grenada**	2030	11.26	11.67	0.40	3.12	11.47	0.00	0.98	0.51	0.14
**Guam**	1970	12.68	11.86	-0.82	3.68	12.27	0.02	0.96	0.71	0.23
**Guam**	2018	13.76	14.10	0.33	2.97	13.93	0.01	0.99	0.89	0.40
**Guam**	2030	14.12	14.64	0.52	2.86	14.38	0.00	0.99	0.92	0.49
**Guatemala**	1970	3.11	2.08	-1.03	3.45	2.60	0.47	0.21	0.04	0.02
**Guatemala**	2018	7.63	6.72	-0.92	5.39	7.18	0.12	0.66	0.21	0.09
**Guatemala**	2030	8.97	8.26	-0.71	5.00	8.61	0.05	0.80	0.28	0.13
**Guinea**	1970	1.29	0.22	-1.07	1.42	0.76	0.91	0.06	0.03	0.02
**Guinea**	2018	6.59	2.52	-4.06	5.78	4.56	0.49	0.39	0.17	0.07
**Guinea**	2030	9.21	4.87	-4.34	6.26	7.04	0.24	0.59	0.31	0.10
**Guinea-Bissau**	1970	1.10	0.27	-0.84	1.26	0.69	0.87	0.05	0.01	0.01
**Guinea-Bissau**	2018	6.59	3.52	-3.07	5.45	5.06	0.35	0.45	0.14	0.05
**Guinea-Bissau**	2030	8.42	6.05	-2.37	5.52	7.24	0.14	0.64	0.25	0.08
**Guyana**	1970	7.10	6.41	-0.68	4.36	6.76	0.06	0.64	0.12	0.05
**Guyana**	2018	10.73	11.29	0.56	3.84	11.01	0.01	0.94	0.51	0.14
**Guyana**	2030	11.87	12.41	0.54	3.32	12.14	0.01	0.97	0.72	0.20
**Haiti**	1970	1.98	1.06	-0.91	2.49	1.52	0.72	0.12	0.02	0.01
**Haiti**	2018	8.05	6.75	-1.30	5.43	7.40	0.12	0.66	0.22	0.09
**Haiti**	2030	9.53	8.38	-1.15	5.16	8.95	0.04	0.78	0.32	0.14
**Honduras**	1970	3.25	2.84	-0.42	3.60	3.05	0.36	0.24	0.04	0.02
**Honduras**	2018	7.05	7.23	0.18	5.13	7.14	0.09	0.67	0.20	0.09
**Honduras**	2030	7.96	8.35	0.39	4.94	8.15	0.05	0.76	0.24	0.12
**Hungary**	1970	10.34	9.52	-0.82	3.54	9.93	0.01	0.91	0.37	0.09
**Hungary**	2018	12.30	12.80	0.50	2.35	12.55	0.00	0.99	0.81	0.20
**Hungary**	2030	12.47	13.36	0.89	2.45	12.92	0.00	0.99	0.86	0.26
**Iceland**	1970	12.91	11.35	-1.56	3.81	12.13	0.01	0.98	0.61	0.29
**Iceland**	2018	14.30	14.91	0.61	3.08	14.60	0.01	0.99	0.86	0.60
**Iceland**	2030	14.13	14.55	0.42	3.43	14.34	0.01	0.99	0.82	0.58
**India**	1970	4.43	1.70	-2.73	4.29	3.06	0.58	0.24	0.06	0.03
**India**	2018	8.96	8.44	-0.52	6.22	8.70	0.18	0.70	0.35	0.20
**India**	2030	10.43	11.38	0.95	5.47	10.91	0.07	0.84	0.52	0.33
**Indonesia**	1970	5.10	2.97	-2.13	4.13	4.03	0.31	0.37	0.07	0.02
**Indonesia**	2018	10.52	10.73	0.20	4.17	10.63	0.01	0.94	0.56	0.15
**Indonesia**	2030	11.55	11.92	0.37	3.76	11.73	0.00	0.98	0.65	0.22
**Iran**	1970	2.99	1.13	-1.87	3.13	2.06	0.64	0.17	0.04	0.01
**Iran**	2018	11.84	10.57	-1.27	5.98	11.20	0.14	0.84	0.62	0.31
**Iran**	2030	13.62	13.67	0.05	4.16	13.64	0.04	0.96	0.75	0.44
**Iraq**	1970	4.78	1.17	-3.61	4.49	2.97	0.65	0.27	0.11	0.05
**Iraq**	2018	12.51	10.50	-2.01	5.97	11.50	0.14	0.84	0.65	0.33
**Iraq**	2030	13.90	13.20	-0.70	4.41	13.55	0.05	0.94	0.79	0.45
**Ireland**	1970	9.27	9.61	0.34	3.78	9.44	0.01	0.98	0.32	0.11
**Ireland**	2018	13.42	14.12	0.69	3.18	13.77	0.00	0.99	0.79	0.43

**Ireland**	2030	13.96	14.91	0.95	3.08	14.44	0.00	0.99	0.84	0.53
**Israel**	1970	11.01	11.27	0.26	5.33	11.14	0.09	0.89	0.53	0.25
**Israel**	2018	12.83	13.26	0.43	4.54	13.05	0.04	0.95	0.71	0.40
**Israel**	2030	12.86	13.68	0.82	4.62	13.27	0.05	0.94	0.72	0.43
**Italy**	1970	8.22	7.07	-1.15	4.34	7.64	0.06	0.62	0.21	0.07
**Italy**	2018	12.14	12.79	0.65	3.52	12.47	0.00	0.99	0.72	0.23
**Italy**	2030	12.63	13.74	1.10	3.54	13.19	0.00	0.99	0.79	0.32
**Jamaica**	1970	6.71	6.76	0.05	3.36	6.73	0.02	0.75	0.11	0.03
**Jamaica**	2018	12.01	12.89	0.88	3.04	12.45	0.00	0.99	0.69	0.21
**Jamaica**	2030	12.82	13.67	0.85	2.74	13.24	0.00	0.99	0.84	0.27
**Japan**	1970	11.71	10.80	-0.91	3.09	11.25	0.00	0.99	0.50	0.15
**Japan**	2018	14.42	14.58	0.16	2.97	14.50	0.01	0.99	0.89	0.56
**Japan**	2030	14.53	14.79	0.26	3.49	14.66	0.02	0.98	0.87	0.62
**Jordan**	1970	7.57	3.03	-4.54	5.75	5.30	0.36	0.49	0.17	0.07
**Jordan**	2018	13.41	12.91	-0.50	4.50	13.16	0.05	0.94	0.77	0.42
**Jordan**	2030	14.84	14.53	-0.31	3.88	14.68	0.04	0.96	0.90	0.60
**Kazakhstan**	1970	9.68	9.04	-0.64	4.16	9.36	0.02	0.85	0.28	0.11
**Kazakhstan**	2018	12.17	13.03	0.86	2.87	12.60	0.00	0.99	0.65	0.28
**Kazakhstan**	2030	12.42	13.50	1.08	2.95	12.96	0.00	0.99	0.67	0.31
**Kenya**	1970	4.96	2.02	-2.94	4.18	3.49	0.45	0.31	0.04	0.01
**Kenya**	2018	10.65	9.56	-1.08	5.18	10.10	0.08	0.86	0.47	0.17
**Kenya**	2030	12.29	12.05	-0.23	4.67	12.17	0.03	0.94	0.65	0.34
**Kiribati**	1970	5.70	5.32	-0.38	3.59	5.51	0.03	0.46	0.06	0.02
**Kiribati**	2018	10.09	10.75	0.67	3.19	10.42	0.00	0.96	0.36	0.09
**Kiribati**	2030	10.82	11.45	0.63	2.75	11.14	0.00	0.98	0.45	0.09
**Kuwait**	1970	6.21	3.46	-2.74	5.22	4.84	0.32	0.46	0.13	0.05
**Kuwait**	2018	13.19	12.95	-0.24	4.36	13.07	0.05	0.94	0.76	0.39
**Kuwait**	2030	14.43	14.33	-0.10	3.76	14.38	0.03	0.97	0.86	0.52
**Kyrgyzstan**	1970	10.12	8.99	-1.13	4.28	9.55	0.03	0.86	0.27	0.13
**Kyrgyzstan**	2018	12.40	13.18	0.78	2.93	12.79	0.00	0.99	0.69	0.28
**Kyrgyzstan**	2030	12.57	13.49	0.92	2.78	13.03	0.00	0.99	0.74	0.27
**Laos**	1970	2.80	0.76	-2.03	2.79	1.78	0.70	0.15	0.02	0.01
**Laos**	2018	7.09	5.80	-1.29	5.53	6.45	0.22	0.58	0.19	0.07
**Laos**	2030	8.39	7.72	-0.67	5.42	8.05	0.10	0.71	0.28	0.11
**Latvia**	1970	11.90	12.28	0.38	3.20	12.09	0.01	0.98	0.60	0.22
**Latvia**	2018	13.65	14.50	0.85	2.94	14.07	0.01	0.99	0.91	0.43
**Latvia**	2030	13.67	14.95	1.29	3.16	14.31	0.01	0.99	0.87	0.49
**Lebanon**	1970	7.16	6.11	-1.05	5.17	6.63	0.15	0.61	0.18	0.07
**Lebanon**	2018	13.24	12.83	-0.41	4.59	13.03	0.06	0.93	0.75	0.41
**Lebanon**	2030	14.55	14.50	-0.05	3.89	14.52	0.04	0.96	0.86	0.56
**Lesotho**	1970	2.94	4.04	1.09	3.46	3.49	0.25	0.26	0.03	0.01
**Lesotho**	2018	8.35	9.83	1.48	4.27	9.09	0.04	0.85	0.30	0.08
**Lesotho**	2030	9.58	10.64	1.06	3.67	10.11	0.01	0.93	0.36	0.10

**Liberia**	1970	3.49	0.64	-2.85	3.44	2.06	0.77	0.18	0.07	0.03
**Liberia**	2018	9.36	6.92	-2.44	6.74	8.14	0.26	0.67	0.40	0.16
**Liberia**	2030	11.11	9.66	-1.45	5.97	10.39	0.12	0.82	0.54	0.26
**Libya**	1970	3.47	0.67	-2.80	3.14	2.07	0.61	0.16	0.04	0.02
**Libya**	2018	12.87	11.55	-1.32	5.47	12.21	0.11	0.88	0.71	0.37
**Libya**	2030	13.77	13.00	-0.76	4.62	13.39	0.06	0.93	0.80	0.44
**Lithuania**	1970	11.94	11.95	0.01	3.82	11.95	0.02	0.91	0.68	0.20
**Lithuania**	2018	14.13	14.61	0.49	2.59	14.37	0.01	0.99	0.95	0.50
**Lithuania**	2030	14.62	15.22	0.60	2.69	14.92	0.01	0.98	0.95	0.64
**Luxembourg**	1970	12.30	11.24	-1.07	3.62	11.77	0.01	0.96	0.63	0.19
**Luxembourg**	2018	14.07	14.15	0.08	3.20	14.11	0.01	0.99	0.86	0.48
**Luxembourg**	2030	14.65	14.96	0.31	2.94	14.80	0.01	0.99	0.91	0.60
**Macedonia**	1970	10.31	7.56	-2.76	4.61	8.94	0.04	0.80	0.32	0.09
**Macedonia**	2018	12.54	12.66	0.12	2.93	12.60	0.01	0.99	0.75	0.22
**Macedonia**	2030	12.90	13.25	0.34	2.77	13.08	0.00	0.99	0.81	0.26
**Madagascar**	1970	3.56	2.21	-1.35	3.48	2.89	0.39	0.19	0.03	0.01
**Madagascar**	2018	9.15	8.37	-0.78	5.28	8.76	0.10	0.78	0.33	0.11
**Madagascar**	2030	10.85	10.10	-0.75	4.71	10.47	0.04	0.89	0.48	0.18
**Malawi**	1970	3.81	1.38	-2.43	3.35	2.60	0.46	0.18	0.02	0.01
**Malawi**	2018	9.77	7.77	-2.00	4.79	8.77	0.06	0.80	0.31	0.09
**Malawi**	2030	11.29	9.46	-1.83	4.25	10.38	0.01	0.92	0.43	0.15
**Malaysia**	1970	5.03	2.87	-2.16	4.22	3.95	0.33	0.35	0.06	0.02
**Malaysia**	2018	10.49	11.15	0.66	4.34	10.82	0.03	0.92	0.49	0.17
**Malaysia**	2030	11.49	12.33	0.84	3.81	11.91	0.01	0.97	0.61	0.23
**Maldives**	1970	1.42	1.10	-0.32	2.17	1.26	0.81	0.11	0.02	0.01
**Maldives**	2018	6.50	7.02	0.52	5.07	6.76	0.14	0.63	0.18	0.06
**Maldives**	2030	7.74	9.02	1.27	4.74	8.38	0.03	0.77	0.27	0.09
**Mali**	1970	0.75	0.23	-0.52	0.94	0.49	0.93	0.04	0.01	0.01
**Mali**	2018	3.83	1.95	-1.88	4.25	2.89	0.62	0.24	0.08	0.03
**Mali**	2030	6.51	3.33	-3.18	5.71	4.92	0.39	0.41	0.16	0.06
**Malta**	1970	8.50	7.20	-1.31	3.86	7.85	0.03	0.86	0.16	0.07
**Malta**	2018	12.63	12.80	0.16	2.85	12.71	0.00	0.99	0.54	0.26
**Malta**	2030	13.08	13.72	0.64	3.01	13.40	0.00	0.99	0.64	0.37
**Marshall Islands**	1970	6.91	7.42	0.51	4.83	7.17	0.10	0.67	0.18	0.06
**Marshall Islands**	2018	11.36	11.94	0.58	3.80	11.65	0.02	0.96	0.61	0.20
**Marshall Islands**	2030	11.96	12.61	0.65	3.37	12.29	0.01	0.98	0.68	0.22
**Mauritania**	1970	3.82	1.95	-1.87	3.95	2.88	0.54	0.24	0.05	0.02
**Mauritania**	2018	9.17	7.64	-1.53	5.76	8.40	0.14	0.73	0.33	0.13
**Mauritania**	2030	11.15	10.12	-1.03	4.96	10.63	0.04	0.88	0.51	0.22
**Mauritius**	1970	5.53	3.35	-2.19	3.96	4.44	0.20	0.35	0.06	0.02
**Mauritius**	2018	10.76	11.22	0.47	4.11	10.99	0.02	0.92	0.55	0.14
**Mauritius**	2030	11.59	12.06	0.47	3.62	11.83	0.01	0.97	0.61	0.18
**Mexico**	1970	4.61	3.78	-0.83	4.45	4.19	0.28	0.40	0.07	0.05

**Mexico**	2018	10.16	9.65	-0.51	4.46	9.90	0.02	0.92	0.36	0.18
**Mexico**	2030	10.90	10.47	-0.43	3.99	10.68	0.01	0.96	0.42	0.20
**Moldova**	1970	11.02	10.27	-0.75	4.11	10.64	0.02	0.91	0.41	0.15
**Moldova**	2018	13.95	14.41	0.47	3.08	14.18	0.01	0.99	0.88	0.47
**Moldova**	2030	14.62	15.16	0.54	3.01	14.89	0.00	0.99	0.93	0.58
**Mongolia**	1970	7.75	6.17	-1.58	4.31	6.96	0.04	0.63	0.15	0.06
**Mongolia**	2018	9.05	10.63	1.58	3.82	9.84	0.01	0.90	0.28	0.13
**Mongolia**	2030	9.66	11.09	1.43	3.71	10.37	0.01	0.93	0.33	0.15
**Montenegro**	1970	11.76	9.33	-2.42	4.22	10.55	0.04	0.90	0.47	0.13
**Montenegro**	2018	13.94	14.20	0.26	2.95	14.07	0.01	0.99	0.90	0.43
**Montenegro**	2030	14.56	14.92	0.36	2.90	14.74	0.00	0.99	0.93	0.53
**Morocco**	1970	1.74	0.54	-1.21	2.00	1.14	0.83	0.08	0.02	0.01
**Morocco**	2018	8.08	7.15	-0.92	6.72	7.62	0.29	0.63	0.37	0.14
**Morocco**	2030	10.93	11.62	0.69	5.39	11.27	0.10	0.87	0.66	0.28
**Mozambique**	1970	1.92	0.64	-1.28	1.98	1.28	0.66	0.05	0.01	0.00
**Mozambique**	2018	6.44	5.25	-1.19	4.79	5.85	0.16	0.53	0.12	0.04
**Mozambique**	2030	8.27	7.59	-0.68	4.64	7.93	0.04	0.75	0.21	0.08
**Myanmar**	1970	3.13	1.86	-1.27	3.44	2.50	0.55	0.20	0.03	0.01
**Myanmar**	2018	6.98	7.42	0.44	4.82	7.20	0.09	0.66	0.19	0.06
**Myanmar**	2030	8.29	9.17	0.88	4.80	8.73	0.03	0.78	0.30	0.11
**Namibia**	1970	4.42	3.61	-0.82	4.50	4.01	0.36	0.34	0.07	0.02
**Namibia**	2018	9.99	10.10	0.11	3.95	10.04	0.04	0.90	0.42	0.09
**Namibia**	2030	10.97	11.02	0.05	3.25	11.00	0.01	0.95	0.51	0.11
**Nepal**	1970	1.57	0.25	-1.32	1.64	0.91	0.85	0.07	0.01	0.00
**Nepal**	2018	7.51	4.89	-2.62	5.57	6.20	0.26	0.52	0.16	0.07
**Nepal**	2030	9.19	8.23	-0.96	5.39	8.71	0.07	0.74	0.31	0.16
**Netherlands**	1970	11.68	10.30	-1.38	4.14	10.99	0.01	0.94	0.51	0.17
**Netherlands**	2018	14.46	14.78	0.32	2.84	14.62	0.00	0.99	0.89	0.61
**Netherlands**	2030	14.77	15.30	0.52	2.49	15.03	0.00	0.99	0.92	0.72
**New Zealand**	1970	11.96	11.28	-0.69	4.52	11.62	0.05	0.92	0.62	0.24
**New Zealand**	2018	13.74	14.26	0.52	3.16	14.00	0.01	0.99	0.85	0.46
**New Zealand**	2030	13.76	14.59	0.83	3.34	14.17	0.01	0.98	0.85	0.50
**Nicaragua**	1970	3.52	2.83	-0.69	3.91	3.18	0.41	0.27	0.05	0.03
**Nicaragua**	2018	7.71	8.07	0.36	5.31	7.89	0.09	0.73	0.25	0.11
**Nicaragua**	2030	8.75	9.18	0.42	5.01	8.96	0.05	0.82	0.33	0.13
**Niger**	1970	0.30	0.21	-0.09	0.50	0.25	0.97	0.02	0.01	0.00
**Niger**	2018	2.62	1.21	-1.41	3.06	1.91	0.71	0.15	0.04	0.01
**Niger**	2030	3.87	1.85	-2.02	4.03	2.86	0.55	0.22	0.06	0.02
**Nigeria**	1970	3.45	1.44	-2.02	3.78	2.45	0.68	0.23	0.07	0.03
**Nigeria**	2018	9.81	7.85	-1.96	6.37	8.83	0.21	0.73	0.46	0.17
**Nigeria**	2030	11.40	10.29	-1.11	5.34	10.85	0.09	0.86	0.58	0.25
**North Korea**	1970	7.58	6.20	-1.37	3.97	6.89	0.05	0.68	0.12	0.04
**North Korea**	2018	11.88	12.97	1.09	3.34	12.43	0.00	0.98	0.69	0.24

**North Korea**	2030	12.29	13.82	1.53	3.21	13.06	0.00	0.98	0.76	0.30
**Northern Mariana Islands**	1970	10.31	8.66	-1.65	6.02	9.48	0.15	0.78	0.43	0.19
**Northern Mariana Islands**	2018	13.77	13.84	0.07	3.28	13.80	0.01	0.98	0.88	0.42
**Northern Mariana Islands**	2030	14.28	14.43	0.15	3.01	14.36	0.01	0.99	0.92	0.50
**Norway**	1970	13.04	12.26	-0.78	3.37	12.65	0.01	0.98	0.69	0.27
**Norway**	2018	14.58	14.99	0.42	2.59	14.79	0.01	0.99	0.94	0.60
**Norway**	2030	14.53	15.02	0.49	2.94	14.77	0.01	0.98	0.93	0.62
**Oman**	1970	2.28	0.62	-1.66	2.25	1.45	0.65	0.08	0.01	0.01
**Oman**	2018	12.22	10.62	-1.60	6.02	11.42	0.13	0.85	0.67	0.34
**Oman**	2030	13.94	13.28	-0.66	4.30	13.61	0.04	0.95	0.80	0.44
**Pakistan**	1970	3.45	0.80	-2.65	3.38	2.12	0.71	0.17	0.05	0.02
**Pakistan**	2018	8.33	6.21	-2.12	6.34	7.27	0.26	0.60	0.28	0.13
**Pakistan**	2030	10.11	9.69	-0.42	5.84	9.90	0.09	0.79	0.45	0.25
**Palestine**	1970	6.88	3.27	-3.61	5.12	5.08	0.28	0.45	0.12	0.05
**Palestine**	2018	13.24	13.21	-0.04	3.85	13.22	0.03	0.96	0.79	0.38
**Palestine**	2030	14.19	14.71	0.52	3.20	14.45	0.01	0.98	0.90	0.52
**Panama**	1970	6.84	6.69	-0.14	5.40	6.76	0.14	0.63	0.19	0.09
**Panama**	2018	11.82	12.31	0.49	3.88	12.07	0.02	0.95	0.67	0.24
**Panama**	2030	12.36	12.69	0.33	3.20	12.53	0.01	0.98	0.71	0.23
**Papua New Guinea**	1970	1.46	0.71	-0.76	1.86	1.08	0.79	0.09	0.01	0.00
**Papua New Guinea**	2018	4.85	3.83	-1.02	4.40	4.34	0.28	0.38	0.07	0.02
**Papua New Guinea**	2030	5.73	5.19	-0.54	4.60	5.46	0.16	0.49	0.10	0.03
**Paraguay**	1970	5.69	5.07	-0.62	4.36	5.38	0.11	0.42	0.10	0.05
**Paraguay**	2018	10.73	10.94	0.22	4.70	10.83	0.02	0.89	0.53	0.21
**Paraguay**	2030	11.68	12.14	0.46	3.88	11.91	0.01	0.95	0.65	0.24
**Peru**	1970	7.28	4.95	-2.33	5.59	6.12	0.18	0.47	0.16	0.09
**Peru**	2018	10.82	10.32	-0.50	4.35	10.57	0.02	0.89	0.38	0.15
**Peru**	2030	11.27	11.01	-0.26	4.03	11.14	0.01	0.93	0.44	0.16
**Philippines**	1970	7.56	6.93	-0.63	4.85	7.25	0.07	0.67	0.20	0.07
**Philippines**	2018	11.10	12.41	1.31	3.89	11.76	0.02	0.94	0.63	0.22
**Philippines**	2030	12.35	13.43	1.08	3.82	12.89	0.01	0.97	0.73	0.31
**Poland**	1970	9.98	9.63	-0.35	3.89	9.81	0.01	0.94	0.34	0.09
**Poland**	2018	12.82	13.82	1.01	2.70	13.32	0.00	0.99	0.84	0.31
**Poland**	2030	13.29	14.91	1.62	2.66	14.10	0.00	0.99	0.92	0.44
**Portugal**	1970	6.10	5.13	-0.97	4.08	5.61	0.09	0.48	0.10	0.04
**Portugal**	2018	11.17	12.03	0.85	4.27	11.60	0.01	0.97	0.56	0.24
**Portugal**	2030	12.08	13.04	0.96	3.92	12.56	0.01	0.99	0.68	0.30
**Puerto Rico**	1970	9.99	9.70	-0.29	4.95	9.84	0.04	0.82	0.46	0.14
**Puerto Rico**	2018	13.56	14.12	0.56	3.32	13.84	0.01	0.98	0.87	0.43
**Puerto Rico**	2030	14.45	14.54	0.08	3.20	14.50	0.01	0.98	0.90	0.54
**Qatar**	1970	5.47	2.30	-3.17	5.23	3.88	0.49	0.40	0.18	0.07
**Qatar**	2018	12.11	10.63	-1.48	6.13	11.37	0.13	0.86	0.67	0.34
**Qatar**	2030	13.59	11.40	-2.19	5.28	12.49	0.06	0.93	0.74	0.39

**Romania**	1970	10.01	8.49	-1.53	4.64	9.25	0.06	0.82	0.32	0.10
**Romania**	2018	12.50	13.07	0.57	3.53	12.79	0.01	0.97	0.69	0.30
**Romania**	2030	13.08	13.91	0.83	3.63	13.50	0.01	0.97	0.78	0.41
**Russia**	1970	11.56	11.80	0.24	3.04	11.68	0.00	0.96	0.55	0.12
**Russia**	2018	14.21	14.63	0.42	3.01	14.42	0.00	0.99	0.87	0.52
**Russia**	2030	15.28	15.46	0.17	2.85	15.37	0.00	0.99	0.93	0.69
**Rwanda**	1970	2.58	0.85	-1.73	2.51	1.72	0.60	0.11	0.01	0.00
**Rwanda**	2018	7.27	5.55	-1.72	4.87	6.41	0.14	0.59	0.15	0.05
**Rwanda**	2030	8.69	6.71	-1.97	4.71	7.70	0.06	0.72	0.21	0.07
**Saint Lucia**	1970	5.23	4.98	-0.25	4.13	5.11	0.11	0.43	0.08	0.03
**Saint Lucia**	2018	10.13	11.13	1.00	4.12	10.63	0.02	0.92	0.46	0.15
**Saint Lucia**	2030	11.05	11.85	0.80	3.64	11.45	0.01	0.96	0.56	0.17
**Saint Vincent and the Grenadines**	1970	5.13	4.99	-0.14	3.31	5.06	0.01	0.42	0.06	0.02
**Saint Vincent and the Grenadines**	2018	9.91	10.37	0.47	3.87	10.14	0.00	0.93	0.36	0.11
**Saint Vincent and the Grenadines**	2030	10.66	10.91	0.25	3.45	10.79	0.00	0.97	0.41	0.12
**Samoa**	1970	6.73	6.28	-0.46	4.56	6.51	0.10	0.61	0.14	0.05
**Samoa**	2018	11.43	12.05	0.62	3.37	11.74	0.01	0.97	0.60	0.18
**Samoa**	2030	12.14	12.76	0.62	3.10	12.45	0.00	0.99	0.69	0.22
**Sao Tome and Principe**	1970	3.57	1.66	-1.91	3.44	2.61	0.48	0.19	0.03	0.01
**Sao Tome and Principe**	2018	8.96	8.63	-0.33	4.25	8.80	0.02	0.82	0.27	0.08
**Sao Tome and Principe**	2030	10.21	10.19	-0.02	3.94	10.20	0.00	0.91	0.42	0.11
**Saudi Arabia**	1970	3.70	0.78	-2.92	3.37	2.24	0.62	0.19	0.04	0.02
**Saudi Arabia**	2018	12.43	10.65	-1.77	5.97	11.54	0.14	0.85	0.67	0.33
**Saudi Arabia**	2030	13.94	12.83	-1.11	4.55	13.38	0.06	0.93	0.78	0.43
**Senegal**	1970	1.26	0.38	-0.87	1.51	0.82	0.89	0.07	0.02	0.01
**Senegal**	2018	5.49	3.64	-1.85	5.50	4.57	0.45	0.40	0.14	0.05
**Senegal**	2030	7.77	6.42	-1.35	6.05	7.09	0.23	0.63	0.28	0.09
**Serbia**	1970	10.22	8.38	-1.84	4.29	9.30	0.04	0.85	0.30	0.09
**Serbia**	2018	13.78	14.37	0.59	2.96	14.07	0.01	0.99	0.90	0.43
**Serbia**	2030	14.47	15.11	0.64	2.85	14.79	0.00	0.99	0.94	0.54
**Seychelles**	1970	5.84	5.21	-0.63	5.95	5.53	0.38	0.50	0.19	0.07
**Seychelles**	2018	12.17	13.17	1.01	3.17	12.67	0.01	0.98	0.74	0.25
**Seychelles**	2030	12.64	14.06	1.42	2.84	13.35	0.00	0.99	0.80	0.30
**Sierra Leone**	1970	1.64	0.59	-1.05	2.02	1.11	0.86	0.09	0.03	0.02
**Sierra Leone**	2018	6.49	3.94	-2.54	6.09	5.22	0.44	0.46	0.20	0.07
**Sierra Leone**	2030	9.15	7.53	-1.62	6.05	8.34	0.17	0.71	0.40	0.12
**Singapore**	1970	6.49	4.08	-2.40	5.65	5.29	0.38	0.50	0.15	0.05
**Singapore**	2018	13.20	13.11	-0.09	4.37	13.15	0.05	0.95	0.75	0.40
**Singapore**	2030	13.59	13.69	0.11	4.09	13.64	0.04	0.95	0.80	0.45
**Slovakia**	1970	11.45	10.72	-0.73	2.42	11.09	0.00	0.99	0.42	0.07
**Slovakia**	2018	12.97	13.65	0.68	2.36	13.31	0.00	0.99	0.91	0.23
**Slovakia**	2030	13.10	14.69	1.59	2.82	13.89	0.00	0.98	0.94	0.34
**Slovenia**	1970	11.49	10.36	-1.12	3.81	10.92	0.02	0.94	0.52	0.12

**Slovenia**	2018	13.73	14.71	0.97	2.81	14.22	0.00	0.99	0.92	0.41
**Slovenia**	2030	14.22	15.53	1.31	2.80	14.88	0.00	0.99	0.95	0.53
**Solomon Islands**	1970	2.98	1.06	-1.92	3.19	2.02	0.69	0.18	0.04	0.01
**Solomon Islands**	2018	7.69	6.35	-1.33	5.80	7.02	0.22	0.64	0.23	0.09
**Solomon Islands**	2030	9.08	8.18	-0.91	5.56	8.63	0.11	0.76	0.33	0.13
**Somalia**	1970	2.28	0.49	-1.79	2.36	1.39	0.78	0.11	0.03	0.01
**Somalia**	2018	7.39	3.88	-3.50	6.18	5.63	0.38	0.48	0.21	0.08
**Somalia**	2030	9.38	6.85	-2.53	6.27	8.11	0.15	0.67	0.33	0.15
**South Africa**	1970	6.71	5.50	-1.21	5.58	6.11	0.29	0.56	0.19	0.04
**South Africa**	2018	11.55	11.63	0.08	2.84	11.59	0.01	0.98	0.59	0.13
**South Africa**	2030	12.75	12.80	0.05	3.04	12.77	0.00	0.99	0.73	0.24
**South Korea**	1970	10.02	6.50	-3.51	5.57	8.26	0.14	0.74	0.30	0.11
**South Korea**	2018	14.33	14.57	0.24	3.35	14.45	0.02	0.98	0.88	0.56
**South Korea**	2030	14.30	14.65	0.35	3.62	14.48	0.02	0.97	0.86	0.60
**South Sudan**	1970	1.02	0.22	-0.80	1.17	0.62	0.89	0.05	0.02	0.01
**South Sudan**	2018	6.55	1.69	-4.86	5.35	4.12	0.48	0.34	0.14	0.05
**South Sudan**	2030	9.22	3.59	-5.63	6.05	6.41	0.20	0.52	0.23	0.09
**Spain**	1970	6.09	5.00	-1.09	5.43	5.54	0.34	0.58	0.15	0.06
**Spain**	2018	11.05	12.11	1.06	4.68	11.58	0.04	0.93	0.58	0.26
**Spain**	2030	11.05	12.74	1.68	4.72	11.89	0.03	0.93	0.60	0.29
**Sri Lanka**	1970	6.05	5.06	-1.00	4.87	5.55	0.21	0.52	0.12	0.04
**Sri Lanka**	2018	10.82	12.09	1.27	4.22	11.45	0.03	0.93	0.60	0.21
**Sri Lanka**	2030	11.82	12.99	1.18	3.66	12.40	0.01	0.97	0.69	0.26
**Sudan**	1970	2.96	0.96	-2.00	3.04	1.96	0.67	0.15	0.03	0.01
**Sudan**	2018	11.26	9.81	-1.45	6.35	10.54	0.18	0.80	0.59	0.27
**Sudan**	2030	13.55	13.13	-0.42	4.42	13.34	0.05	0.94	0.78	0.42
**Suriname**	1970	5.84	4.59	-1.25	5.03	5.22	0.25	0.46	0.11	0.05
**Suriname**	2018	10.05	9.86	-0.18	4.94	9.95	0.06	0.86	0.42	0.16
**Suriname**	2030	11.10	11.00	-0.10	4.39	11.05	0.03	0.92	0.52	0.20
**Swaziland**	1970	4.16	3.10	-1.06	4.34	3.63	0.43	0.30	0.06	0.02
**Swaziland**	2018	9.96	9.63	-0.33	4.04	9.79	0.04	0.88	0.39	0.09
**Swaziland**	2030	10.88	10.43	-0.45	3.37	10.65	0.01	0.94	0.47	0.10
**Sweden**	1970	11.68	11.86	0.18	3.25	11.77	0.00	0.99	0.56	0.20
**Sweden**	2018	14.00	14.70	0.71	2.74	14.35	0.00	0.99	0.85	0.57
**Sweden**	2030	14.53	15.38	0.85	2.75	14.95	0.00	0.99	0.87	0.68
**Switzerland**	1970	14.18	13.10	-1.09	3.24	13.64	0.00	0.99	0.84	0.37
**Switzerland**	2018	14.68	14.92	0.24	2.91	14.80	0.01	0.99	0.91	0.64
**Switzerland**	2030	14.49	14.83	0.33	3.08	14.66	0.01	0.98	0.87	0.66
**Syria**	1970	4.69	1.63	-3.07	4.20	3.16	0.51	0.27	0.06	0.02
**Syria**	2018	12.74	10.93	-1.81	5.78	11.83	0.13	0.86	0.68	0.35
**Syria**	2030	14.08	12.85	-1.23	4.67	13.46	0.07	0.93	0.79	0.45
**Taiwan**	1970	8.17	5.37	-2.80	4.52	6.77	0.10	0.67	0.15	0.04
**Taiwan**	2018	14.70	14.91	0.21	2.96	14.80	0.01	0.99	0.93	0.58

**Taiwan**	2030	15.66	15.97	0.31	2.50	15.81	0.00	0.99	0.96	0.76
**Tajikistan**	1970	9.87	7.35	-2.53	5.01	8.61	0.08	0.77	0.26	0.10
**Tajikistan**	2018	11.85	11.12	-0.73	3.04	11.49	0.01	0.97	0.44	0.16
**Tajikistan**	2030	12.37	11.68	-0.69	3.05	12.03	0.01	0.98	0.53	0.19
**Tanzania**	1970	3.43	1.11	-2.31	3.12	2.27	0.56	0.16	0.02	0.01
**Tanzania**	2018	8.37	7.37	-1.00	5.31	7.87	0.13	0.73	0.26	0.08
**Tanzania**	2030	9.38	9.18	-0.20	4.28	9.28	0.04	0.89	0.30	0.10
**Thailand**	1970	5.27	4.03	-1.23	3.40	4.65	0.10	0.24	0.07	0.03
**Thailand**	2018	10.89	12.22	1.33	3.79	11.55	0.01	0.96	0.60	0.19
**Thailand**	2030	11.43	12.60	1.17	3.21	12.02	0.00	0.98	0.61	0.19
**The Bahamas**	1970	8.46	8.35	-0.11	5.24	8.40	0.08	0.75	0.28	0.11
**The Bahamas**	2018	12.97	13.58	0.61	3.03	13.28	0.01	0.99	0.83	0.32
**The Bahamas**	2030	13.64	14.33	0.69	2.72	13.98	0.00	0.99	0.92	0.40
**The Gambia**	1970	1.25	0.51	-0.74	1.64	0.88	0.90	0.07	0.03	0.01
**The Gambia**	2018	7.52	5.40	-2.12	6.56	6.46	0.36	0.55	0.29	0.10
**The Gambia**	2030	9.92	9.64	-0.28	5.71	9.78	0.10	0.81	0.51	0.18
**Timor-Leste**	1970	0.87	0.30	-0.57	1.08	0.58	0.89	0.04	0.01	0.00
**Timor-Leste**	2018	7.97	7.16	-0.80	5.70	7.56	0.17	0.68	0.27	0.10
**Timor-Leste**	2030	9.63	9.71	0.08	5.30	9.67	0.07	0.83	0.42	0.19
**Togo**	1970	2.54	0.67	-1.87	2.62	1.61	0.73	0.13	0.02	0.01
**Togo**	2018	9.29	5.53	-3.76	5.81	7.41	0.18	0.66	0.26	0.10
**Togo**	2030	11.38	8.16	-3.23	5.25	9.77	0.06	0.83	0.43	0.18
**Tonga**	1970	6.89	6.02	-0.88	4.25	6.46	0.07	0.62	0.13	0.04
**Tonga**	2018	11.74	12.17	0.42	3.33	11.95	0.01	0.98	0.64	0.19
**Tonga**	2030	12.42	12.92	0.50	3.03	12.67	0.00	0.99	0.71	0.25
**Trinidad and Tobago**	1970	8.11	7.57	-0.54	4.39	7.84	0.05	0.76	0.20	0.06
**Trinidad and Tobago**	2018	12.13	12.73	0.60	3.09	12.43	0.01	0.98	0.72	0.21
**Trinidad and Tobago**	2030	12.74	13.42	0.68	2.94	13.08	0.00	0.99	0.81	0.28
**Tunisia**	1970	3.56	1.05	-2.51	3.39	2.31	0.62	0.18	0.04	0.01
**Tunisia**	2018	11.50	10.59	-0.91	5.50	11.05	0.10	0.87	0.58	0.27
**Tunisia**	2030	12.78	13.39	0.61	4.16	13.08	0.03	0.96	0.71	0.38
**Turkey**	1970	5.91	3.06	-2.85	4.83	4.48	0.35	0.35	0.09	0.04
**Turkey**	2018	13.20	12.11	-1.10	4.63	12.66	0.06	0.93	0.72	0.36
**Turkey**	2030	14.70	14.12	-0.59	3.68	14.41	0.02	0.97	0.84	0.52
**Turkmenistan**	1970	9.48	7.72	-1.76	4.45	8.60	0.03	0.80	0.25	0.09
**Turkmenistan**	2018	11.02	11.28	0.26	3.35	11.15	0.00	0.96	0.41	0.16
**Turkmenistan**	2030	11.22	11.65	0.43	3.17	11.44	0.00	0.97	0.44	0.17
**Uganda**	1970	4.38	1.44	-2.94	3.74	2.91	0.47	0.23	0.03	0.01
**Uganda**	2018	10.01	8.22	-1.79	5.12	9.12	0.09	0.81	0.36	0.12
**Uganda**	2030	11.43	10.74	-0.68	4.15	11.09	0.02	0.93	0.56	0.17
**Ukraine**	1970	11.99	11.62	-0.38	3.81	11.81	0.00	0.97	0.55	0.22
**Ukraine**	2018	14.13	14.57	0.44	3.02	14.35	0.00	0.99	0.89	0.50
**Ukraine**	2030	14.43	14.92	0.49	2.92	14.68	0.00	0.99	0.94	0.53

**United Arab Emirates**	1970	5.83	2.32	-3.51	4.74	4.07	0.32	0.43	0.12	0.05
**United Arab Emirates**	2018	12.91	11.71	-1.20	5.42	12.31	0.09	0.90	0.72	0.39
**United Arab Emirates**	2030	13.75	12.02	-1.73	5.11	12.89	0.08	0.92	0.79	0.44
**United Kingdom**	1970	11.51	10.62	-0.88	4.64	11.06	0.04	0.92	0.53	0.21
**United Kingdom**	2018	13.68	13.96	0.28	3.64	13.82	0.01	0.98	0.76	0.43
**United Kingdom**	2030	14.09	14.43	0.34	3.42	14.26	0.00	0.99	0.81	0.48
**United States**	1970	12.99	12.58	-0.42	3.03	12.79	0.01	0.98	0.81	0.25
**United States**	2018	13.44	13.65	0.20	2.84	13.54	0.00	0.99	0.89	0.34
**United States**	2030	13.61	13.87	0.26	2.80	13.74	0.00	0.99	0.90	0.37
**Uruguay**	1970	7.34	7.49	0.15	4.46	7.42	0.03	0.72	0.20	0.07
**Uruguay**	2018	10.46	10.90	0.44	4.30	10.68	0.01	0.95	0.44	0.18
**Uruguay**	2030	11.15	11.71	0.56	4.27	11.43	0.00	0.97	0.52	0.24
**Uzbekistan**	1970	10.56	9.08	-1.47	4.03	9.82	0.02	0.89	0.29	0.13
**Uzbekistan**	2018	12.90	13.74	0.84	2.97	13.32	0.00	0.99	0.80	0.33
**Uzbekistan**	2030	13.13	14.38	1.25	2.93	13.76	0.00	0.99	0.86	0.37
**Vanuatu**	1970	4.30	2.65	-1.65	4.15	3.48	0.41	0.30	0.05	0.02
**Vanuatu**	2018	8.66	8.51	-0.15	5.09	8.59	0.08	0.79	0.30	0.11
**Vanuatu**	2030	9.86	9.99	0.13	4.62	9.92	0.04	0.89	0.40	0.15
**Venezuela**	1970	5.52	4.99	-0.52	5.04	5.25	0.24	0.49	0.11	0.05
**Venezuela**	2018	10.26	10.33	0.08	4.71	10.30	0.04	0.89	0.45	0.17
**Venezuela**	2030	11.22	11.48	0.26	4.10	11.35	0.02	0.94	0.56	0.19
**Vietnam**	1970	7.33	4.40	-2.94	4.58	5.87	0.12	0.49	0.13	0.03
**Vietnam**	2018	10.72	11.17	0.45	4.04	10.95	0.02	0.93	0.52	0.16
**Vietnam**	2030	11.77	12.11	0.34	3.55	11.94	0.01	0.97	0.61	0.21
**Virgin Islands, U.S.**	1970	11.59	11.88	0.29	3.85	11.74	0.02	0.94	0.64	0.20
**Virgin Islands, U.S.**	2018	13.97	14.63	0.66	3.07	14.30	0.01	0.99	0.90	0.51
**Virgin Islands, U.S.**	2030	14.61	15.21	0.59	2.83	14.91	0.01	0.99	0.93	0.61
**Yemen**	1970	1.13	0.23	-0.90	1.26	0.68	0.88	0.05	0.01	0.00
**Yemen**	2018	9.57	6.24	-3.34	7.03	7.90	0.31	0.64	0.42	0.17
**Yemen**	2030	12.23	11.76	-0.47	4.98	12.00	0.08	0.90	0.72	0.31
**Zambia**	1970	5.31	2.03	-3.28	4.18	3.67	0.36	0.30	0.05	0.02
**Zambia**	2018	11.18	9.72	-1.45	4.70	10.45	0.05	0.88	0.48	0.16
**Zambia**	2030	12.44	11.49	-0.95	4.12	11.96	0.02	0.96	0.60	0.26
**Zimbabwe**	1970	5.24	3.17	-2.07	4.15	4.21	0.27	0.36	0.04	0.02
**Zimbabwe**	2018	10.75	10.22	-0.53	3.47	10.49	0.01	0.95	0.33	0.10
**Zimbabwe**	2030	11.66	11.15	-0.52	3.15	11.40	0.00	0.98	0.44	0.13

Supplement Table 1. Country Specific Indicators for 1970,2018, and 2030

A number of characteristics of the distribution of educational attainment are shown for 1970, 2018, and 2030, at the national level. All values represent individuals age 25-29.

Country	Data Sources
**Afghanistan**	CENSUS_1979; DHS_2010; DHS_2016;
**Albania**	ALBOR_FORCE_SURVEY_2007; ALBOR_FORCE_SURVEY_2008; ALBOR_FORCE_SURVEY_2009; ALBOR_FORCE_SURVEY_2012; CENSUS_2011; DHS_2009; DHS_2018; EUROBAROMETER_2014; EUROBAROMETER_2015; MICS_2000; MICS_2005; RHS_2002; WB_LSMS_2002; WB_LSMS_2005; WB_LSMS_2008;
**Algeria**	AFROBAROMETER_2013; ARAB_BAROMETER_2014; ARAB_LEAGUE_PAPCHILD_1992; MICS_2013;
**American Samoa**	CENSUS_2000; CENSUS_2010;
**Angola**	DHS_2016; MACRO_MIS_2007; MICS_2001;
**Antigua and Barbuda**	CENSUS_1990; CENSUS_2001; DYB_2001;
**Argentina**	ARGMANENT_HH_SURVEY_EPH_2003; ARGMANENT_HH_SURVEY_EPH_2004; ARGMANENT_HH_SURVEY_EPH_2005; ARGMANENT_HH_SURVEY_EPH_2006; ARGMANENT_HH_SURVEY_EPH_2007; ARGMANENT_HH_SURVEY_EPH_2008; ARGMANENT_HH_SURVEY_EPH_2009; ARGMANENT_HH_SURVEY_EPH_2010; ARGMANENT_HH_SURVEY_EPH_2011; ARGMANENT_HH_SURVEY_EPH_2012; ARGMANENT_HH_SURVEY_EPH_2013; ARGMANENT_HH_SURVEY_EPH_2014; ARGMANENT_HH_SURVEY_EPH_2015; IPUMS_1980; IPUMS_1991; IPUMS_2002; IPUMS_2010; ISSP_2008; ISSP_2010; ISSP_2013; LATINOBAROMETER_1998; LATINOBAROMETER_2000; LATINOBAROMETER_2001; LATINOBAROMETER_2002; LATINOBAROMETER_2003; LATINOBAROMETER_2004; LATINOBAROMETER_2005; LATINOBAROMETER_2006; LATINOBAROMETER_2007; LATINOBAROMETER_2008; LATINOBAROMETER_2009; PERMANENT_HH_SURVEY_EPH_2016;
**Armenia**	ARMEGRATED_LIVING_CONDITIONS_SURVEY_2015; ARMOR_FORCE_SURVEY_2014; ARMOR_FORCE_SURVEY_2015; DHS_2000; DHS_2010; DHS_2016; HEALTH_IN_TIMES_OF_TRANSITION_HITT_CIS_2010; INTEGRATED_LIVING_CONDITIONS_SURVEY_2014; IPUMS_2001; IPUMS_2011; WB_STEP_HH_SURVEY_2013;
**Australia**	CENSUS_2011; ISSP_1989; ISSP_1993; ISSP_1994; ISSP_1996; ISSP_1997; ISSP_2000; ISSP_2002; ISSP_2003; ISSP_2005; ISSP_2007; ISSP_2010; ISSP_2012; ISSP_2013; ISSP_2015; ISSP_2016; WHO_WHS_2003;
**Austria**	EUROBAROMETER_1995; EUROBAROMETER_1996; EUROBAROMETER_1997; EUROBAROMETER_1998; EUROBAROMETER_1999; EUROBAROMETER_2000; EUROBAROMETER_2001; EUROBAROMETER_2002; EUROBAROMETER_2003; EUROBAROMETER_2004; EUROBAROMETER_2005; EUROBAROMETER_2006; EUROBAROMETER_2007; EUROBAROMETER_2008; EUROBAROMETER_2009; EUROBAROMETER_2010; EUROBAROMETER_2011; EUROBAROMETER_2012; EUROBAROMETER_2013; EUROBAROMETER_2014; EUROBAROMETER_2015; EUROSTAT_1998; EUROSTAT_1999; EUROSTAT_2000; EUROSTAT_2001; EUROSTAT_2002; EUROSTAT_2003; EUROSTAT_2004; EUROSTAT_2005; EUROSTAT_2006; EUROSTAT_2007; EUROSTAT_2008; EUROSTAT_2010; EUROSTAT_2011; EUROSTAT_2012; EUROSTAT_2013; EUROSTAT_2014; EUROSTAT_2015; EUROSTAT_2016; IPUMS_1971; IPUMS_1981; IPUMS_1991; IPUMS_2001; IPUMS_2011; ISSP_1989; ISSP_1993; ISSP_1996; ISSP_1999; ISSP_2000; ISSP_2002; ISSP_2003; ISSP_2004; ISSP_2008; ISSP_2010; ISSP_2013; ISSP_2016;
**Azerbaijan**	CENSUS_1999; CENSUS_2006; CENSUS_2011; DHS_2006; DYB_1999; DYB_2009; HEALTH_IN_TIMES_OF_TRANSITION_HITT_CIS_2010; MICS_2000; WB_LSMS_1995;
**Bahrain**	CENSUS_1971; CENSUS_1981; DYB_1991; DYB_2001; DYB_2010;
**Bangladesh**	BGDNDPUR_COMILLA_VERBAL_AUTOPSY_STUDY_2014; DHS_1994; DHS_1997; DHS_2000; DHS_2001; DHS_2004; DHS_2007; DHS_2012; DHS_2014; IPUMS_1991; IPUMS_2001; IPUMS_2011; ISSP_1998; MICS_2006; MICS_2013; SURVEY_2013; WFS_1975; WHO_WHS_2003;
**Barbados**	CENSUS_1980; CENSUS_1990; CENSUS_2000; MICS_2012; PAHO_SABE_2000;
**Belarus**	DYB_1989; DYB_1999; DYB_2001; DYB_2009; HEALTH_IN_TIMES_OF_TRANSITION_HITT_CIS_2010; IPUMS_1999; IPUMS_2009; MICS_2005; MICS_2012;
**Belgium**	EUROBAROMETER_1990; EUROBAROMETER_1992; EUROBAROMETER_1993; EUROBAROMETER_1994; EUROBAROMETER_1995; EUROBAROMETER_1996; EUROBAROMETER_1997; EUROBAROMETER_1998; EUROBAROMETER_1999; EUROBAROMETER_2000; EUROBAROMETER_2001; EUROBAROMETER_2002; EUROBAROMETER_2003; EUROBAROMETER_2004; EUROBAROMETER_2005; EUROBAROMETER_2006; EUROBAROMETER_2007; EUROBAROMETER_2008; EUROBAROMETER_2009; EUROBAROMETER_2010; EUROBAROMETER_2011; EUROBAROMETER_2012; EUROBAROMETER_2013; EUROBAROMETER_2014; EUROBAROMETER_2015; EUROSTAT_1998; EUROSTAT_2007; EUROSTAT_2008; EUROSTAT_2009; EUROSTAT_2010; EUROSTAT_2011; EUROSTAT_2012; EUROSTAT_2013; EUROSTAT_2014; EUROSTAT_2015; EUROSTAT_2016; ISSP_2002; ISSP_2005; ISSP_2007; ISSP_2008; ISSP_2009; ISSP_2010; ISSP_2012; ISSP_2014; ISSP_2016; MCSS_2001;

**Belize**	CDC_RHS_1991; CENSUS_1980; CENSUS_1990; CENSUS_1991; CENSUS_2000; GENDER_ALCOHOL_CULTURE_INTERTIOL_STUDY_GECIS_2005; MICS_2006; MICS_2011; MICS_2016;
**Benin**	AFROBAROMETER SURVEY_2014; AFROBAROMETER_2005; AFROBAROMETER_2008; AFROBAROMETER_2011; AFROBAROMETER_2017; DHS_1996; DHS_2001; DHS_2006; DHS_2012; IPUMS_1979; IPUMS_1992; IPUMS_2002; IPUMS_2013; WFS_1981;
**Bermuda**	DYB_1991; DYB_2000; DYB_2010;
**Bhutan**	BTNING_STANDARDS_2012; BTNING_STANDARDS_2017; MICS_2010;
**Bolivia**	BOL_STEP_SKILLS_MEASUREMENT_HOUSEHOLD_SURVEY_2012_WAVE_1_HH_ROSTER_Y2017M05D16.DTA_2012; DHS_1989; DHS_1994; DHS_1998; DHS_2004; DHS_2008; EDSA_2016; IPUMS_1976; IPUMS_1992; IPUMS_2001; LATINOBAROMETER_1998; LATINOBAROMETER_2000; LATINOBAROMETER_2002; LATINOBAROMETER_2003; LATINOBAROMETER_2004; LATINOBAROMETER_2005; LATINOBAROMETER_2006; LATINOBAROMETER_2007; LATINOBAROMETER_2008; LATINOBAROMETER_2009; LATINOBAROMETER_2010; LATINOBAROMETER_2011; LATINOBAROMETER_2013; LATINOBAROMETER_2015; LATINOBAROMETER_2016; LATINOBAROMETER_2017; MICS_2000;
**Bosnia and Herzegovina**	MICS_2000; MICS_2006; WHO_WHS_2003;
**Botswana**	AFROBAROMETER SURVEY_2014; AFROBAROMETER_1999; AFROBAROMETER_2008; AFROBAROMETER_2012; AFROBAROMETER_2017; AIDS_IMPACT_SURVEY_2013; BWAS_IMPACT_SURVEY_2001; BWAS_IMPACT_SURVEY_2004; BWAS_IMPACT_SURVEY_2008; CENSUS_2004; DYB_1991; IPUMS_1991; IPUMS_2001; IPUMS_2011; LABOUR FORCE SURVEY_2006; MICS_2000; STEPS_2007;
**Brazil**	BRAIOL_DEMOGRAPHIC_HEALTH_SURVEY_OF_CHILDREN_AND_WOMEN_2007; BRAIOL_HEALTH_SURVEY_PNS_2013; BRASUMER_EXPENDITURE_SURVEY_POF_2003; BRASUMER_EXPENDITURE_SURVEY_POF_2009; CENSUS_2000; DHS_1986; DHS_1996; GLOBAL_ADULT_TOBACCO_SURVEY_2008; IPUMS_1960; IPUMS_1970; IPUMS_1980; IPUMS_1991; IPUMS_2000; IPUMS_2010; LATINOBAROMETER_1998; LATINOBAROMETER_2000; LATINOBAROMETER_2001; LATINOBAROMETER_2002; LATINOBAROMETER_2003; LATINOBAROMETER_2004; LATINOBAROMETER_2005; LATINOBAROMETER_2006; LATINOBAROMETER_2008; LATINOBAROMETER_2009; LATINOBAROMETER_2010; LATINOBAROMETER_2011; LATINOBAROMETER_2013; LATINOBAROMETER_2015; LATINOBAROMETER_2016; LATINOBAROMETER_2017; SURVEY_1968; SURVEY_1999; SURVEY_2009; WHO_WHS_2003;
**Brunei**	CENSUS_1981; CENSUS_2001;
**Bulgaria**	EUROBAROMETER_2004; EUROBAROMETER_2005; EUROBAROMETER_2006; EUROBAROMETER_2007; EUROBAROMETER_2008; EUROBAROMETER_2009; EUROBAROMETER_2010; EUROBAROMETER_2011; EUROBAROMETER_2012; EUROBAROMETER_2013; EUROBAROMETER_2014; EUROBAROMETER_2015; ISSP_1993; ISSP_1994; ISSP_1996; ISSP_1997; ISSP_1999; ISSP_2002; ISSP_2003; ISSP_2004; ISSP_2005; ISSP_2009; ISSP_2011; WB_LSMS_1995; WB_LSMS_1997; WB_LSMS_2001; WB_LSMS_2003; WB_LSMS_2007;
**Burkina Faso**	AFROBAROMETER SURVEY_2014; AFROBAROMETER_2017; DHS_1993; DHS_1999; DHS_2003; DHS_2011; IPUMS_1985; IPUMS_1996; IPUMS_2006; MACRO_MIS_2014; MICS_2006; WHO_WHS_2003;
**Burundi**	AFROBAROMETER SURVEY_2014; DHS_2011; DHS_2017; DYB_1990; MACRO_MIS_2013; MICS_2000; MICS_2005;
**Cambodia**	CDC_VACS_2013; DHS_2000; DHS_2006; DHS_2011; DHS_2014; IPUMS_1998; IPUMS_2008; KHMERCENSAL_POPULATION_SURVEY_2004; KHMERCENSAL_POPULATION_SURVEY_2013; KHMIO_ECONOMIC_SURVEY_1994; KHMIO_ECONOMIC_SURVEY_2005; KHMOR_FORCE_SURVEY_2000; KHMOR_FORCE_SURVEY_2001;
**Cameroon**	AFROBAROMETER_2017; DHS_1991; DHS_1998; DHS_2004; DHS_2011; IPUMS_1976; IPUMS_1987; IPUMS_2005; MICS_2000; MICS_2006; MICS_2014; WFS_1978;
**Canada**	IPUMS_1971; IPUMS_1981; IPUMS_1991; IPUMS_2001; IPUMS_2011; ISSP_1994; ISSP_1996; ISSP_1999; ISSP_2000; ISSP_2002; ISSP_2004; ISSP_2006; ISSP_2011; ISSP_2014; MCSS_2001;
**Cape Verde**	AFROBAROMETER SURVEY_2014; AFROBAROMETER_2002; AFROBAROMETER_2005; AFROBAROMETER_2008; AFROBAROMETER_2011; AFROBAROMETER_2017; CDC_RHS_1998; DHS_2005; RHS_1998;
**Central African Republic**	DHS_1995; MICS_2000; MICS_2006; MICS_2011;
**Chad**	DHS_1997; DHS_2004; DHS_2015; MICS_2000; MICS_2010; WHS_2003;
**Chile**	IPUMS_1960; IPUMS_1970; IPUMS_1992; IPUMS_2002; ISSP_1998; ISSP_2000; ISSP_2002; ISSP_2003; ISSP_2005; ISSP_2006; ISSP_2007; ISSP_2008; ISSP_2009; ISSP_2010; ISSP_2011; ISSP_2012; ISSP_2014; ISSP_2015; LATINOBAROMETER_1998; LATINOBAROMETER_2000; LATINOBAROMETER_2001; LATINOBAROMETER_2002; LATINOBAROMETER_2003; LATINOBAROMETER_2004; LATINOBAROMETER_2005; LATINOBAROMETER_2006; LATINOBAROMETER_2007; LATINOBAROMETER_2008; LATINOBAROMETER_2009; SURVEY_ADULT_SKILLS_2015;

**China**	CENSUS_2010; CGSS_2015; CHN_CHRONIC_DISEASE_RISK_FACTOR_SURVEILLANCE_2010; GLOBAL_ADULT_TOBACCO_SURVEY_2010; IPUMS_1982; IPUMS_1990; IPUMS_2000; ISSP_2008; ISSP_2011; ISSP_2012; ISSP_2015; MCSS_2001; WB_LSMS_1997; WB_STEP_HH_SURVEY_2012; WHO_SAGE_2010; WHO_WHS_2002;
**Colombia**	COLIOL_HEALTH_SURVEY_ENS_2008; DHS_1995; DHS_2000; DHS_2005; DHS_2010; IPUMS_1964; IPUMS_1973; IPUMS_1986; IPUMS_1994; IPUMS_2006; LATINOBAROMETER_1998; LATINOBAROMETER_2000; LATINOBAROMETER_2001; LATINOBAROMETER_2002; LATINOBAROMETER_2003; LATINOBAROMETER_2004; LATINOBAROMETER_2006; LATINOBAROMETER_2007; LATINOBAROMETER_2008; LATINOBAROMETER_2009; LATINOBAROMETER_2011; LATINOBAROMETER_2013; LATINOBAROMETER_2015; LATINOBAROMETER_2016; LATINOBAROMETER_2017; MCSS_2001; WFS_1976;
**Comoros**	DHS_1996; DHS_2013; MICS_2000; WHO_WHS_2003;
**Congo**	DHS_2005; DHS_2012; MACRO_AIS_2009; MICS_2015; WHO_WHS_2003;
**Costa Rica**	CDC_RHS_1993; CRIIOL_HOUSEHOLD_SURVEY_EHO_2015; IPUMS_1963; IPUMS_1973; IPUMS_1984; IPUMS_2000; IPUMS_2011; LATINOBAROMETER_1998; LATINOBAROMETER_2000; LATINOBAROMETER_2001; LATINOBAROMETER_2002; LATINOBAROMETER_2003; LATINOBAROMETER_2004; LATINOBAROMETER_2005; LATINOBAROMETER_2006; LATINOBAROMETER_2007; LATINOBAROMETER_2008; LATINOBAROMETER_2009; LATINOBAROMETER_2010; LATINOBAROMETER_2011; LATINOBAROMETER_2013; LATINOBAROMETER_2015; LATINOBAROMETER_2016; LATINOBAROMETER_2017; MCSS_2001; MICS_2011; RHS_1991; WFS_1976;
**Cote d'Ivoire**	DHS_1994; DHS_1999; DHS_2012; MACRO_AIS_2005; MICS_2000; MICS_2006; MICS_2016; WFS_1980; WHO_WHS_2003;
**Croatia**	EUROBAROMETER_2004; EUROBAROMETER_2005; EUROBAROMETER_2006; EUROBAROMETER_2007; EUROBAROMETER_2008; EUROBAROMETER_2009; EUROBAROMETER_2010; EUROBAROMETER_2011; EUROBAROMETER_2012; EUROBAROMETER_2013; EUROBAROMETER_2014; EUROBAROMETER_2015; EUROSTAT_2002; EUROSTAT_2003; EUROSTAT_2004; EUROSTAT_2005; EUROSTAT_2006; EUROSTAT_2007; EUROSTAT_2008; EUROSTAT_2009; EUROSTAT_2010; EUROSTAT_2011; EUROSTAT_2012; EUROSTAT_2013; EUROSTAT_2014; EUROSTAT_2015; EUROSTAT_2016; ISSP_2006; ISSP_2009; ISSP_2011; ISSP_2013; ISSP_2014; ISSP_2015; ISSP_2016; MCSS_2001; WHO_WHS_2003;
**Cuba**	IPUMS_2002; MICS_2006; MICS_2011; MICS_2014; PAHO_SABE_2000;
**Cyprus**	EUROBAROMETER_2004; EUROBAROMETER_2005; EUROBAROMETER_2006; EUROBAROMETER_2007; EUROBAROMETER_2008; EUROBAROMETER_2009; EUROBAROMETER_2010; EUROBAROMETER_2011; EUROBAROMETER_2012; EUROBAROMETER_2013; EUROBAROMETER_2014; EUROBAROMETER_2015; EUROSTAT_1999; EUROSTAT_2008; EUROSTAT_2014; EUROSTAT_2015; EUROSTAT_2016; ISSP_1996; ISSP_1997; ISSP_1998; ISSP_1999; ISSP_2002; ISSP_2004; ISSP_2005; ISSP_2007; ISSP_2008; ISSP_2012; MCSS_2001;
**Czech Republic**	EUROBAROMETER_2002; EUROBAROMETER_2004; EUROBAROMETER_2005; EUROBAROMETER_2006; EUROBAROMETER_2007; EUROBAROMETER_2008; EUROBAROMETER_2009; EUROBAROMETER_2010; EUROBAROMETER_2011; EUROBAROMETER_2012; EUROBAROMETER_2013; EUROBAROMETER_2014; EUROBAROMETER_2015; EUROSTAT_1998; EUROSTAT_1999; EUROSTAT_2007; EUROSTAT_2008; EUROSTAT_2009; EUROSTAT_2010; EUROSTAT_2011; EUROSTAT_2012; EUROSTAT_2013; EUROSTAT_2014; EUROSTAT_2015; EUROSTAT_2016; ISSP_1993; ISSP_1994; ISSP_1996; ISSP_1997; ISSP_1999; ISSP_2002; ISSP_2003; ISSP_2004; ISSP_2005; ISSP_2006; ISSP_2007; ISSP_2008; ISSP_2010; ISSP_2012; ISSP_2014; ISSP_2015; MCSS_2001; RHS_1993; SURVEY_ADULT_SKILLS_2012; WHO_WHS_2003;
**Democratic Republic of the Congo**	DHS_2007; DHS_2014; MICS_2001; MICS_2010;
**Denmark**	EUROBAROMETER_1990; EUROBAROMETER_1992; EUROBAROMETER_1993; EUROBAROMETER_1994; EUROBAROMETER_1995; EUROBAROMETER_1996; EUROBAROMETER_1997; EUROBAROMETER_1998; EUROBAROMETER_1999; EUROBAROMETER_2000; EUROBAROMETER_2001; EUROBAROMETER_2002; EUROBAROMETER_2003; EUROBAROMETER_2004; EUROBAROMETER_2005; EUROBAROMETER_2006; EUROBAROMETER_2007; EUROBAROMETER_2008; EUROBAROMETER_2009; EUROBAROMETER_2010; EUROBAROMETER_2011; EUROBAROMETER_2012; EUROBAROMETER_2013; EUROBAROMETER_2014; EUROBAROMETER_2015; EUROSTAT_1998; EUROSTAT_1999; EUROSTAT_2008; EUROSTAT_2009; EUROSTAT_2010; EUROSTAT_2011; EUROSTAT_2012; EUROSTAT_2013; EUROSTAT_2014; EUROSTAT_2015; EUROSTAT_2016; ISSP_1997; ISSP_2000; ISSP_2002; ISSP_2003; ISSP_2004; ISSP_2005; ISSP_2006; ISSP_2008; ISSP_2009; ISSP_2010; ISSP_2011; ISSP_2013; ISSP_2014; ISSP_2015; ISSP_2016; MCSS_2001;
**Djibouti**	MICS_2006;
**Dominica**	CENSUS_1981;

**Dominican Republic**	DHS_1991; DHS_1996; DHS_1999; DHS_2002; DHS_2007; DHS_2013; IPUMS_1960; IPUMS_1970; IPUMS_1981; IPUMS_2002; IPUMS_2010; ISSP_2005; ISSP_2006; ISSP_2007; ISSP_2008; LATINOBAROMETER_2005; LATINOBAROMETER_2006; LATINOBAROMETER_2007; LATINOBAROMETER_2008; LATINOBAROMETER_2009; LATINOBAROMETER_2011; LATINOBAROMETER_2013; LATINOBAROMETER_2015; LATINOBAROMETER_2016; LATINOBAROMETER_2017; MICS_2000; MICS_2014; WFS_1975; WFS_1980; WHO_WHS_2003;
**Ecuador**	CDC_RHS_1994; CDC_RHS_1999; CDC_RHS_2004; IPUMS_1962; IPUMS_1974; IPUMS_1982; IPUMS_1990; IPUMS_2001; IPUMS_2010; LATINOBAROMETER_1998; LATINOBAROMETER_2000; LATINOBAROMETER_2001; LATINOBAROMETER_2002; LATINOBAROMETER_2003; LATINOBAROMETER_2004; LATINOBAROMETER_2005; LATINOBAROMETER_2006; LATINOBAROMETER_2007; LATINOBAROMETER_2008; LATINOBAROMETER_2009; LATINOBAROMETER_2010; LATINOBAROMETER_2011; LATINOBAROMETER_2013; LATINOBAROMETER_2015; LATINOBAROMETER_2016; LATINOBAROMETER_2017; WB_LSMS_1994; WFS_1979; WHO_WHS_2003;
**Egypt**	AFROBAROMETER_2013; ARAB_BAROMETER_2014; DHS_1993; DHS_1996; DHS_2000; DHS_2003; DHS_2005; DHS_2008; DHS_2014; IPUMS_1996; IPUMS_2006; MICS_2014; WFS_1980;
**El Salvador**	CDC_RHS_1993; CDC_RHS_1998; CDC_RHS_2003; CDC_RHS_2008; IPUMS_1992; IPUMS_2007; LATINOBAROMETER_2001; LATINOBAROMETER_2003; LATINOBAROMETER_2004; LATINOBAROMETER_2005; LATINOBAROMETER_2006; LATINOBAROMETER_2007; LATINOBAROMETER_2008; LATINOBAROMETER_2009; LATINOBAROMETER_2010; LATINOBAROMETER_2011; LATINOBAROMETER_2013; LATINOBAROMETER_2015; LATINOBAROMETER_2016; LATINOBAROMETER_2017; MICS_2014; SURVEY_2015;
**Equatorial Guinea**	CENSUS_2011; MICS_2000;
**Eritrea**	CENSUS_1995; CENSUS_2002; CENSUS_2010; DHS_1996; DHS_2002;
**Estonia**	EUROBAROMETER_2004; EUROBAROMETER_2005; EUROBAROMETER_2006; EUROBAROMETER_2007; EUROBAROMETER_2008; EUROBAROMETER_2009; EUROBAROMETER_2010; EUROBAROMETER_2011; EUROBAROMETER_2012; EUROBAROMETER_2013; EUROBAROMETER_2014; EUROBAROMETER_2015; EUROSTAT_1998; EUROSTAT_1999; EUROSTAT_2007; EUROSTAT_2008; EUROSTAT_2009; EUROSTAT_2010; EUROSTAT_2011; EUROSTAT_2012; EUROSTAT_2013; EUROSTAT_2014; EUROSTAT_2015; EUROSTAT_2016; ISSP_2010; ISSP_2013; ISSP_2015; WHO_WHS_2003;
**Ethiopia**	DHS_2000; DHS_2005; DHS_2011; DHS_2014; DHS_2016; ETHFARE_MONITORING_SURVEY_2004; ETHLTH_NUTRITION_SURVEY_1998; IPUMS_1994; IPUMS_2007;
**Federated States of Micronesia**	CENSUS_2000; CENSUS_2006;
**Fiji**	IPUMS_1976; IPUMS_1986; IPUMS_1996; IPUMS_2007; WFS_1974;
**Finland**	EUROBAROMETER_1995; EUROBAROMETER_1996; EUROBAROMETER_1997; EUROBAROMETER_1998; EUROBAROMETER_1999; EUROBAROMETER_2000; EUROBAROMETER_2001; EUROBAROMETER_2002; EUROBAROMETER_2003; EUROBAROMETER_2004; EUROBAROMETER_2005; EUROBAROMETER_2006; EUROBAROMETER_2007; EUROBAROMETER_2008; EUROBAROMETER_2009; EUROBAROMETER_2010; EUROBAROMETER_2011; EUROBAROMETER_2012; EUROBAROMETER_2013; EUROBAROMETER_2014; EUROBAROMETER_2015; EUROSTAT_1998; EUROSTAT_1999; EUROSTAT_2007; EUROSTAT_2008; EUROSTAT_2009; EUROSTAT_2010; EUROSTAT_2011; EUROSTAT_2012; EUROSTAT_2013; EUROSTAT_2014; EUROSTAT_2015; EUROSTAT_2016; ISSP_2002; ISSP_2003; ISSP_2004; ISSP_2005; ISSP_2006; ISSP_2007; ISSP_2009; ISSP_2011; ISSP_2012; ISSP_2013; ISSP_2014; ISSP_2015; WHS_2004;
**France**	EUROBAROMETER_1990; EUROBAROMETER_1992; EUROBAROMETER_1993; EUROBAROMETER_1994; EUROBAROMETER_1995; EUROBAROMETER_1996; EUROBAROMETER_1997; EUROBAROMETER_1998; EUROBAROMETER_1999; EUROBAROMETER_2000; EUROBAROMETER_2001; EUROBAROMETER_2002; EUROBAROMETER_2003; EUROBAROMETER_2004; EUROBAROMETER_2005; EUROBAROMETER_2006; EUROBAROMETER_2007; EUROBAROMETER_2008; EUROBAROMETER_2009; EUROBAROMETER_2010; EUROBAROMETER_2011; EUROBAROMETER_2012; EUROBAROMETER_2013; EUROBAROMETER_2014; EUROBAROMETER_2015; EUROSTAT_1998; EUROSTAT_1999; EUROSTAT_2007; EUROSTAT_2008; EUROSTAT_2009; EUROSTAT_2010; EUROSTAT_2011; EUROSTAT_2012; EUROSTAT_2013; EUROSTAT_2014; EUROSTAT_2015; EUROSTAT_2016; IPUMS_2006; IPUMS_2011; ISSP_1997; ISSP_1998; ISSP_1999; ISSP_2002; ISSP_2003; ISSP_2005; ISSP_2006; ISSP_2007; ISSP_2008; ISSP_2009; ISSP_2010; ISSP_2011; ISSP_2012; ISSP_2013; ISSP_2014; ISSP_2015; MCSS_2001; WHO_WHS_2003;
**Gabon**	AFROBAROMETER SURVEY_2015; AFROBAROMETER_2017; DHS_2001; DHS_2012;
**Georgia**	CDC_RHS_2000; CDC_RHS_2005; CDC_RHS_2011; GEO_2011; HEALTH_IN_TIMES_OF_TRANSITION_HITT_CIS_2010; ISSP_2013; ISSP_2016; RHS_1999; RHS_2005; RHS_2010; WHO_WHS_2003;
**Germany**	EUROBAROMETER_1990; EUROBAROMETER_1992; EUROBAROMETER_1993; EUROBAROMETER_1994; EUROBAROMETER_1995; EUROBAROMETER_1996; EUROBAROMETER_1997; EUROBAROMETER_1998;

	EUROBAROMETER_1999; EUROBAROMETER_2000; EUROBAROMETER_2001; EUROBAROMETER_2002; EUROBAROMETER_2003; EUROBAROMETER_2004; EUROBAROMETER_2005; EUROBAROMETER_2006; EUROBAROMETER_2007; EUROBAROMETER_2008; EUROBAROMETER_2009; EUROBAROMETER_2010; EUROBAROMETER_2011; EUROBAROMETER_2012; EUROBAROMETER_2013; EUROBAROMETER_2014; EUROBAROMETER_2015; EUROSTAT_1999; EUROSTAT_2000; EUROSTAT_2001; EUROSTAT_2002; EUROSTAT_2003; EUROSTAT_2004; EUROSTAT_2005; EUROSTAT_2006; EUROSTAT_2007; EUROSTAT_2008; EUROSTAT_2009; EUROSTAT_2010; EUROSTAT_2011; EUROSTAT_2012; EUROSTAT_2013; EUROSTAT_2014; EUROSTAT_2016; IPUMS_1981; ISSP_1989; ISSP_1992; ISSP_1994; ISSP_1996; ISSP_1998; ISSP_2000; ISSP_2002; ISSP_2004; ISSP_2006; ISSP_2008; ISSP_2010; ISSP_2012; ISSP_2014; ISSP_2016; MCSS_2001;
**Ghana**	AFROBAROMETER SURVEY_2014; AFROBAROMETER_2012; AFROBAROMETER_2017; DHS_1994; DHS_1999; DHS_2003; DHS_2008; DHS_2014; DHS_2017; IPUMS_1984; IPUMS_2000; IPUMS_2010; MACRO_MIS_2016; MICS_2006; WB_LSMS_2013; WFS_1979; WHO_WHS_2003;
**Greece**	EUROBAROMETER_1990; EUROBAROMETER_1992; EUROBAROMETER_1993; EUROBAROMETER_1994; EUROBAROMETER_1995; EUROBAROMETER_1996; EUROBAROMETER_1997; EUROBAROMETER_1998; EUROBAROMETER_1999; EUROBAROMETER_2000; EUROBAROMETER_2001; EUROBAROMETER_2002; EUROBAROMETER_2003; EUROBAROMETER_2004; EUROBAROMETER_2005; EUROBAROMETER_2006; EUROBAROMETER_2007; EUROBAROMETER_2008; EUROBAROMETER_2009; EUROBAROMETER_2010; EUROBAROMETER_2011; EUROBAROMETER_2012; EUROBAROMETER_2013; EUROBAROMETER_2014; EUROBAROMETER_2015; EUROSTAT_1998; EUROSTAT_1999; EUROSTAT_2007; EUROSTAT_2008; EUROSTAT_2009; EUROSTAT_2010; EUROSTAT_2011; EUROSTAT_2012; EUROSTAT_2013; EUROSTAT_2014; EUROSTAT_2015; EUROSTAT_2016; IPUMS_1971; IPUMS_1981; IPUMS_1991; IPUMS_2001; IPUMS_2011; SURVEY_ADULT_SKILLS_2015;
**Greenland**	CENSUS_2010;
**Grenada**	CENSUS_1981; CENSUS_1990; CENSUS_2011;
**Guam**	BRFSS_2001; BRFSS_2002; BRFSS_2003; BRFSS_2007; BRFSS_2008; BRFSS_2009; BRFSS_2010; BRFSS_2012; BRFSS_2013; BRFSS_2014; DYB_1990; USASS_2015;
**Guatemala**	CDC_RHS_2002; CENSUS_1995; DHS_1995; DHS_1999; DHS_2015; LATINOBAROMETER_2003; LATINOBAROMETER_2004; LATINOBAROMETER_2005; LATINOBAROMETER_2006; LATINOBAROMETER_2007; LATINOBAROMETER_2008; LATINOBAROMETER_2009; LATINOBAROMETER_2010; LATINOBAROMETER_2011; LATINOBAROMETER_2013; LATINOBAROMETER_2015; LATINOBAROMETER_2016; LATINOBAROMETER_2017;
**Guinea**	AFROBAROMETER_2013; DHS_1999; DHS_2005; DHS_2012; IPUMS_1983; IPUMS_1996; MICS_2016;
**Guinea-Bissau**	MICS_2000; MICS_2006; MICS_2010; MICS_2014;
**Guyana**	DHS_2009; MACRO_AIS_2005; MICS_2000; MICS_2007; MICS_2014; WFS_1975;
**Haiti**	DHS_1995; DHS_2000; DHS_2006; DHS_2012; DHS_2017; IPUMS_1982; IPUMS_2003; WFS_1977;
**Honduras**	CDC_RHS_1996; CDC_RHS_2001; DHS_2006; DHS_2012; IPUMS_1961; IPUMS_1974; IPUMS_1988; IPUMS_2001; LATINOBAROMETER_2002; LATINOBAROMETER_2003; LATINOBAROMETER_2004; LATINOBAROMETER_2005; LATINOBAROMETER_2006; LATINOBAROMETER_2007; LATINOBAROMETER_2008; LATINOBAROMETER_2009; LATINOBAROMETER_2010; LATINOBAROMETER_2011; LATINOBAROMETER_2013; LATINOBAROMETER_2015; LATINOBAROMETER_2016; LATINOBAROMETER_2017;
**Hungary**	EUROBAROMETER_2002; EUROBAROMETER_2004; EUROBAROMETER_2005; EUROBAROMETER_2006; EUROBAROMETER_2007; EUROBAROMETER_2008; EUROBAROMETER_2009; EUROBAROMETER_2010; EUROBAROMETER_2011; EUROBAROMETER_2012; EUROBAROMETER_2013; EUROBAROMETER_2014; EUROBAROMETER_2015; EUROSTAT_1998; EUROSTAT_1999; EUROSTAT_2007; EUROSTAT_2008; EUROSTAT_2009; EUROSTAT_2010; EUROSTAT_2011; EUROSTAT_2012; EUROSTAT_2013; EUROSTAT_2014; EUROSTAT_2015; EUROSTAT_2016; IPUMS_1970; IPUMS_1980; IPUMS_1990; IPUMS_2001; ISSP_1989; ISSP_1992; ISSP_1994; ISSP_1996; ISSP_1998; ISSP_2002; ISSP_2003; ISSP_2004; ISSP_2005; ISSP_2006; ISSP_2007; ISSP_2008; ISSP_2009; ISSP_2013; ISSP_2014; ISSP_2015; MCSS_2001; WHO_WHS_2003;
**Iceland**	EUROBAROMETER_2003; EUROBAROMETER_2005; EUROBAROMETER_2010; EUROBAROMETER_2011; EUROBAROMETER_2012; EUROBAROMETER_2013; EUROBAROMETER_2014; EUROSTAT_1999; EUROSTAT_2007; EUROSTAT_2008; EUROSTAT_2009; EUROSTAT_2010; EUROSTAT_2011; EUROSTAT_2012; EUROSTAT_2013; EUROSTAT_2014; EUROSTAT_2015; EUROSTAT_2016; ISSP_2010; ISSP_2014; ISSP_2015; ISSP_2016;
**India**	DHS_1993; DHS_2000; DHS_2006; DHS_2015; DHS_2016; INDAN_DEVELOPMENT_SURVEY_2005; INDIOL_SAMPLE_SURVEY_1994; INDIOL_SAMPLE_SURVEY_2010; INDIOL_SAMPLE_SURVEY_2012; INDTRICT_LEVEL_HOUSEHOLD_SURVEY_2008; INDTRICT_LEVEL_HOUSEHOLD_SURVEY_2014; IPUMS_1983; IPUMS_1988; IPUMS_1994; IPUMS_2000; IPUMS_2005; IPUMS_2010; ISSP_2014; ISSP_2015; ISSP_2016; ISSP_2017; MCSS_2001; NSSO_1987; NSSO_1991; NSSO_2007; NSSO_2014; WHO_SAGE_2007; WHO_WHS_2003;

**Indonesia**	DHS_1991; DHS_1994; DHS_1997; DHS_2003; DHS_2007; DHS_2012; IPUMS_1971; IPUMS_1976; IPUMS_1980; IPUMS_1995; IPUMS_2005; MCSS_2001; MICS_2000;
**Iran**	CENSUS_2016; IPUMS_2006; IPUMS_2011;
**Iraq**	HH_SOCIOECONOMIC_SURVEY_2007; IPUMS_1997; MICS_2000; MICS_2006; MICS_2011;
**Ireland**	EUROBAROMETER_1990; EUROBAROMETER_1992; EUROBAROMETER_1993; EUROBAROMETER_1994; EUROBAROMETER_1995; EUROBAROMETER_1996; EUROBAROMETER_1997; EUROBAROMETER_1998; EUROBAROMETER_1999; EUROBAROMETER_2000; EUROBAROMETER_2001; EUROBAROMETER_2002; EUROBAROMETER_2003; EUROBAROMETER_2004; EUROBAROMETER_2005; EUROBAROMETER_2006; EUROBAROMETER_2007; EUROBAROMETER_2008; EUROBAROMETER_2009; EUROBAROMETER_2010; EUROBAROMETER_2011; EUROBAROMETER_2012; EUROBAROMETER_2013; EUROBAROMETER_2014; EUROBAROMETER_2015; EUROSTAT_1999; EUROSTAT_2007; EUROSTAT_2008; EUROSTAT_2009; EUROSTAT_2010; EUROSTAT_2011; EUROSTAT_2012; EUROSTAT_2013; EUROSTAT_2014; EUROSTAT_2015; EUROSTAT_2016; IPUMS_1971; IPUMS_1981; IPUMS_1991; IPUMS_1996; IPUMS_2002; IPUMS_2006; IPUMS_2011; ISSP_1989; ISSP_1994; ISSP_1996; ISSP_1998; ISSP_2002; ISSP_2003; ISSP_2006; ISSP_2008; ISSP_2013; MCSS_2001; SURVEY_ADULT_SKILLS_2012; WHO_WHS_2003;
**Israel**	IPUMS_1972; IPUMS_1983; IPUMS_1995; ISSP_1989; ISSP_1994; ISSP_1996; ISSP_1999; ISSP_2000; ISSP_2002; ISSP_2004; ISSP_2005; ISSP_2007; ISSP_2010; ISSP_2011; ISSP_2012; ISSP_2014; ISSP_2016; SURVEY_ADULT_SKILLS_2015; WHO_WHS_2003;
**Italy**	EUROBAROMETER_1990; EUROBAROMETER_1992; EUROBAROMETER_1993; EUROBAROMETER_1994; EUROBAROMETER_1995; EUROBAROMETER_1996; EUROBAROMETER_1997; EUROBAROMETER_1998; EUROBAROMETER_1999; EUROBAROMETER_2000; EUROBAROMETER_2001; EUROBAROMETER_2002; EUROBAROMETER_2003; EUROBAROMETER_2004; EUROBAROMETER_2005; EUROBAROMETER_2006; EUROBAROMETER_2007; EUROBAROMETER_2008; EUROBAROMETER_2009; EUROBAROMETER_2010; EUROBAROMETER_2011; EUROBAROMETER_2012; EUROBAROMETER_2013; EUROBAROMETER_2014; EUROBAROMETER_2015; EUROSTAT_2007; EUROSTAT_2008; EUROSTAT_2009; EUROSTAT_2010; EUROSTAT_2011; EUROSTAT_2012; EUROSTAT_2013; EUROSTAT_2014; EUROSTAT_2015; EUROSTAT_2016; IPUMS_2001; IPUMS_2011; ISSP_1989; ISSP_1994; ISSP_1996; ISSP_1997; ISSP_2000; ISSP_2002; ISSP_2010; ISSP_2012; ISSP_2013; ITAD_IALS_2003; ITAECTS_OF_DAILY_LIFE_2014; ITAVEY_OF_HH_INCOME_AND_WEALTH_1978; ITAVEY_OF_HH_INCOME_AND_WEALTH_1990; ITAVEY_OF_HH_INCOME_AND_WEALTH_1992; ITAVEY_OF_HH_INCOME_AND_WEALTH_1994; ITAVEY_OF_HH_INCOME_AND_WEALTH_1996; ITAVEY_OF_HH_INCOME_AND_WEALTH_1999; ITAVEY_OF_HH_INCOME_AND_WEALTH_2001; ITAVEY_OF_HH_INCOME_AND_WEALTH_2003; ITAVEY_OF_HH_INCOME_AND_WEALTH_2005; ITAVEY_OF_HH_INCOME_AND_WEALTH_2007; ITAVEY_OF_HH_INCOME_AND_WEALTH_2009; MCSS_2001; SURVEY_ADULT_SKILLS_2012;
**Jamaica**	CDC_RHS_2003; IPUMS_1982; IPUMS_1991; WB_LSMS_1990; WB_LSMS_1992; WB_LSMS_1993; WB_LSMS_1994; WB_LSMS_1995; WB_LSMS_1996; WB_LSMS_1997; WFS_1975;
**Japan**	CENSUS_2012; ISSP_1994; ISSP_1996; ISSP_1997; ISSP_1998; ISSP_1999; ISSP_2002; ISSP_2003; ISSP_2010; SURVEY_ADULT_SKILLS_2012;
**Jordan**	ARAB_BAROMETER_2014; DHS_1990; DHS_1997; DHS_2007; DHS_2009; DHS_2012; IPUMS_2004;
**Kazakhstan**	DHS_1995; DHS_1999; MICS_2006; MICS_2011; MICS_2015; WHO_WHS_2003;
**Kenya**	AFROBAROMETER SURVEY_2014; AFROBAROMETER_2005; AFROBAROMETER_2016; CENSUS_1989; CENSUS_1999; DHS_1993; DHS_1998; DHS_2003; DHS_2009; DHS_2014; IPUMS_1969; IPUMS_1979; IPUMS_1989; IPUMS_2009; KENBS_2006; KENS_INDICATOR_SURVEY_2007; KENS_INDICATOR_SURVEY_2013; MICS_2000; MICS_2008; WFS_1977; WHS_2004;
**Kiribati**	CENSUS_2004; CENSUS_2015; DYB_2010;
**Kuwait**	ARAB_BAROMETER_2014; DYB_1985; DYB_1995; OTHER_SURVEY_1996; OTHER_SURVEY_2006;
**Kyrgyzstan**	DHS_1997; DHS_2012; HEALTH_IN_TIMES_OF_TRANSITION_HITT_CIS_2011; IPUMS_1999; IPUMS_2009; MICS_2006; MICS_2014; WB_LSMS_1997;
**Laos**	CENSUS_1995; CENSUS_2005; MICS_2000; MICS_2006; MICS_2012; MICS_2017; WHO_WHS_2003;
**Latvia**	CENSUS_2016; EUROBAROMETER_2004; EUROBAROMETER_2005; EUROBAROMETER_2006; EUROBAROMETER_2007; EUROBAROMETER_2008; EUROBAROMETER_2009; EUROBAROMETER_2010; EUROBAROMETER_2011; EUROBAROMETER_2012; EUROBAROMETER_2013; EUROBAROMETER_2014; EUROBAROMETER_2015; EUROSTAT_1999; EUROSTAT_2007; EUROSTAT_2008; EUROSTAT_2009; EUROSTAT_2010; EUROSTAT_2011; EUROSTAT_2012; EUROSTAT_2013; EUROSTAT_2014; EUROSTAT_2015; EUROSTAT_2016; ISSP_1996; ISSP_1998;

	ISSP_1999; ISSP_2002; ISSP_2003; ISSP_2006; ISSP_2007; ISSP_2009; ISSP_2011; ISSP_2013; ISSP_2016; MCSS_2001; WHO_WHS_2003;
**Lebanon**	ARAB_BAROMETER_2014; ARAB_LEAGUE_PAPCHILD_1996; CENSUS_2009; MCSS_2001;
**Lesotho**	AFROBAROMETER SURVEY_2014; AFROBAROMETER_2000; AFROBAROMETER_2005; AFROBAROMETER_2008; AFROBAROMETER_2012; AFROBAROMETER_2017; CENSUS_2001; CENSUS_2004; CENSUS_2009; CENSUS_2014; DHS_2005; DHS_2010; DHS_2014; DYB_2006; IPUMS_1996; IPUMS_2006; MICS_2000; WFS_1977;
**Liberia**	AFROBAROMETER SURVEY_2015; AFROBAROMETER_2008; AFROBAROMETER_2012; DHS_2007; DHS_2013; IPUMS_1974; IPUMS_2008; MACRO_MIS_2011; MACRO_MIS_2016;
**Libya**	ARAB_BAROMETER_2014; ARAB_LEAGUE_PAPFAM_2007;
**Lithuania**	EUROBAROMETER_2004; EUROBAROMETER_2005; EUROBAROMETER_2006; EUROBAROMETER_2007; EUROBAROMETER_2008; EUROBAROMETER_2009; EUROBAROMETER_2010; EUROBAROMETER_2011; EUROBAROMETER_2012; EUROBAROMETER_2013; EUROBAROMETER_2014; EUROBAROMETER_2015; EUROSTAT_1998; EUROSTAT_1999; EUROSTAT_2007; EUROSTAT_2008; EUROSTAT_2009; EUROSTAT_2010; EUROSTAT_2011; EUROSTAT_2012; EUROSTAT_2013; EUROSTAT_2014; EUROSTAT_2015; EUROSTAT_2016; ISSP_2011; ISSP_2013; ISSP_2015; OECD_PIAAC_2015;
**Luxembourg**	EUROBAROMETER_1992; EUROBAROMETER_1993; EUROBAROMETER_1994; EUROBAROMETER_1995; EUROBAROMETER_1996; EUROBAROMETER_1997; EUROBAROMETER_1998; EUROBAROMETER_1999; EUROBAROMETER_2000; EUROBAROMETER_2001; EUROBAROMETER_2002; EUROBAROMETER_2003; EUROBAROMETER_2004; EUROBAROMETER_2005; EUROBAROMETER_2006; EUROBAROMETER_2007; EUROBAROMETER_2008; EUROBAROMETER_2009; EUROBAROMETER_2010; EUROBAROMETER_2011; EUROBAROMETER_2012; EUROBAROMETER_2013; EUROBAROMETER_2014; EUROBAROMETER_2015; MCSS_2001; WHO_WHS_2003;
**Macedonia**	EUROBAROMETER_2007; EUROBAROMETER_2008; EUROBAROMETER_2009; EUROBAROMETER_2010; EUROBAROMETER_2011; EUROBAROMETER_2012; EUROBAROMETER_2013; EUROBAROMETER_2014; EUROBAROMETER_2015; MICS_2011;
**Madagascar**	AFROBAROMETER SURVEY_2015; AFROBAROMETER_2005; AFROBAROMETER_2013; AFROBAROMETER_2018; DHS_1992; DHS_1997; DHS_2004; DHS_2009; MACRO_MIS_2011; MACRO_MIS_2016;
**Malawi**	AFROBAROMETER SURVEY_2014; AFROBAROMETER_2012; AFROBAROMETER_2017; DHS_1992; DHS_2000; DHS_2005; DHS_2010; DHS_2016; IPUMS_1987; IPUMS_1998; IPUMS_2008; MACRO_MIS_2012; MACRO_MIS_2017; MICS_2014; SURVEY_2002; WHO_WHS_2003;
**Malaysia**	IPUMS_1970; IPUMS_1980; IPUMS_1991; IPUMS_2000; MYSILY_LIFE_SURVEYS_1977; WHS_2003;
**Maldives**	CENSUS_1990; CENSUS_2004; CENSUS_2011; DHS_2009; DYB_1985; DYB_1990; DYB_2000; DYB_2006;
**Mali**	AFROBAROMETER SURVEY_2014; AFROBAROMETER_2002; AFROBAROMETER_2005; AFROBAROMETER_2013; AFROBAROMETER_2017; DHS_1996; DHS_2001; DHS_2006; DHS_2013; IPUMS_1987; IPUMS_1998; IPUMS_2009; MACRO_MIS_2015;
**Malta**	EUROBAROMETER_2004; EUROBAROMETER_2005; EUROBAROMETER_2006; EUROBAROMETER_2007; EUROBAROMETER_2008; EUROBAROMETER_2009; EUROBAROMETER_2010; EUROBAROMETER_2011; EUROBAROMETER_2012; EUROBAROMETER_2013; EUROBAROMETER_2014; EUROBAROMETER_2015; EUROSTAT_2009; EUROSTAT_2010; EUROSTAT_2011; EUROSTAT_2012; EUROSTAT_2013; EUROSTAT_2014; EUROSTAT_2015; EUROSTAT_2016;
**Marshall Islands**	CENSUS_2007;
**Mauritania**	MICS_2007; MICS_2015; WFS_1981; WHO_WHS_2003;
**Mauritius**	AFROBAROMETER SURVEY_2014; AFROBAROMETER_2012; AFROBAROMETER_2017; CENSUS_2011; DYB_1990; DYB_2000; DYB_2011; WHO_WHS_2003;
**Mexico**	CENSUS_2000; DHS_1987; IPUMS_1960; IPUMS_1970; IPUMS_1990; IPUMS_1995; IPUMS_2005; IPUMS_2015; ISSP_2002; ISSP_2003; ISSP_2006; ISSP_2007; ISSP_2009; ISSP_2011; ISSP_2013; ISSP_2015; ISSP_2017; LATINOBAROMETER_2001; LATINOBAROMETER_2002; LATINOBAROMETER_2003; LATINOBAROMETER_2004; LATINOBAROMETER_2005; LATINOBAROMETER_2006; LATINOBAROMETER_2007; LATINOBAROMETER_2008; LATINOBAROMETER_2009; LATINOBAROMETER_2010; LATINOBAROMETER_2011; LATINOBAROMETER_2013; LATINOBAROMETER_2015; LATINOBAROMETER_2016; LATINOBAROMETER_2017; MEX_FAMILY_LIFE_SURVEY_2002; MEX_FAMILY_LIFE_SURVEY_2006; MEX_FAMILY_LIFE_SURVEY_2013; MEXIOL_ADDICTION_SURVEY_E_2002; MEXIOL_ADDICTION_SURVEY_E_2008; MEXIOL_ADDICTION_SURVEY_E_2011; MEXIOL_PERFORMANCE_EVALUATION_SURVEY_ENED_2003; MEXIOL_REPRODUCTIVE_HEALTH_SURVEY_ENSAR_2003; MEXVEY_HEALTH_AND_NUTRITION_ENSANUT_2006; MEXVEY_HEALTH_AND_NUTRITION_ENSANUT_2012; MICS_2006; MICS_2009; MICS_2014; MICS_2015; MICS_2016; PAHO_SABE_2000; WFS_1976; WHO_WHS_2003;

**Moldova**	CDC_RHS_1997; CENSUS_2005; DHS_2005; DYB_2004; HEALTH_IN_TIMES_OF_TRANSITION_HITT_CIS_2010; MICS_2000; MICS_2012;
**Mongolia**	IPUMS_1989; IPUMS_2000; MICS_2005; MICS_2010; MICS_2012; MICS_2013; MNGOR_FORCE_SURVEY_2014; RHS_2008;
**Montenegro**	EUROBAROMETER_2011; EUROBAROMETER_2012; EUROBAROMETER_2013; EUROBAROMETER_2014; EUROBAROMETER_2015; MICS_2013;
**Morocco**	AFROBAROMETER SURVEY_2015; AFROBAROMETER_2013; ARAB_BAROMETER_2014; DHS_1992; DHS_2004; IPUMS_1982; IPUMS_1994; IPUMS_2004; WFS_1980;
**Mozambique**	AFROBAROMETER SURVEY_2015; AFROBAROMETER_2002; AFROBAROMETER_2012; DHS_1997; DHS_2004; DHS_2011; IPUMS_1997; IPUMS_2007; MACRO_AIS_2009;
**Myanmar**	CENSUS_1991; CENSUS_1997; CENSUS_2001; CENSUS_2007; DHS_2016; MICS_2000; MICS_2010; WHO_WHS_2003;
**Namibia**	AFROBAROMETER SURVEY_2014; AFROBAROMETER_1999; AFROBAROMETER_2008; AFROBAROMETER_2012; AFROBAROMETER_2017; DHS_1992; DHS_2000; DHS_2007; DHS_2013; WHS_2003;
**Nepal**	DHS_1996; DHS_2001; DHS_2006; DHS_2011; DHS_2017; IPUMS_2001; MICS_2010; MICS_2014; WFS_1976; WHS_2003;
**Netherlands**	EUROBAROMETER_1990; EUROBAROMETER_1992; EUROBAROMETER_1993; EUROBAROMETER_1994; EUROBAROMETER_1995; EUROBAROMETER_1996; EUROBAROMETER_1997; EUROBAROMETER_1998; EUROBAROMETER_1999; EUROBAROMETER_2000; EUROBAROMETER_2001; EUROBAROMETER_2002; EUROBAROMETER_2003; EUROBAROMETER_2004; EUROBAROMETER_2005; EUROBAROMETER_2006; EUROBAROMETER_2007; EUROBAROMETER_2008; EUROBAROMETER_2009; EUROBAROMETER_2010; EUROBAROMETER_2011; EUROBAROMETER_2012; EUROBAROMETER_2013; EUROBAROMETER_2014; EUROBAROMETER_2015; EUROSTAT_1998; EUROSTAT_1999; EUROSTAT_2007; EUROSTAT_2008; EUROSTAT_2009; EUROSTAT_2010; EUROSTAT_2011; EUROSTAT_2012; EUROSTAT_2013; EUROSTAT_2014; EUROSTAT_2015; EUROSTAT_2016; IPUMS_1971; IPUMS_2001; IPUMS_2011; ISSP_1990; ISSP_1994; ISSP_1995; ISSP_1996; ISSP_1998; ISSP_1999; ISSP_2002; ISSP_2003; ISSP_2005; ISSP_2006; ISSP_2008; ISSP_2011; ISSP_2014; MCSS_2001;
**New Zealand**	CENSUS_1986; CENSUS_2006; CENSUS_2013; ISSP_1992; ISSP_1994; ISSP_1996; ISSP_1997; ISSP_1998; ISSP_2002; ISSP_2004; ISSP_2005; ISSP_2006; ISSP_2007; ISSP_2008; ISSP_2010; ISSP_2015; NZLS_ALL_2007;
**Nicaragua**	CDC_RHS_1993; CDC_RHS_2007; DHS_1998; DHS_2001; IPUMS_1971; IPUMS_1995; IPUMS_2005; LATINOBAROMETER_2003; LATINOBAROMETER_2004; LATINOBAROMETER_2005; LATINOBAROMETER_2006; LATINOBAROMETER_2007; LATINOBAROMETER_2008; LATINOBAROMETER_2009; LATINOBAROMETER_2010; LATINOBAROMETER_2011; LATINOBAROMETER_2013; LATINOBAROMETER_2015; LATINOBAROMETER_2016; LATINOBAROMETER_2017; NIC_ENDESA_2012;
**Niger**	AFROBAROMETER SURVEY_2015; AFROBAROMETER_2013; AFROBAROMETER_2018; DHS_1992; DHS_1998; DHS_2006; DHS_2012; MICS_2000; WB_LSMS_2012;
**Nigeria**	AFROBAROMETER SURVEY_2015; AFROBAROMETER_2000; AFROBAROMETER_2001; AFROBAROMETER_2005; AFROBAROMETER_2008; AFROBAROMETER_2012; AFROBAROMETER_2017; DHS_2003; DHS_2008; DHS_2013; IPUMS_2007; IPUMS_2008; IPUMS_2009; IPUMS_2010; MACRO_MIS_2010; MICS_2007; MICS_2011; MICS_2017;
**North Korea**	CENSUS_2008;
**Northern Mariana Islands**	CENSUS_1990; CENSUS_1995; CENSUS_2000; CENSUS_2003; CENSUS_2005;
**Norway**	EUROBAROMETER_1990; EUROBAROMETER_1991; EUROBAROMETER_1992; EUROBAROMETER_1993; EUROBAROMETER_1994; EUROBAROMETER_1995; EUROBAROMETER_1996; EUROBAROMETER_1999; EUROBAROMETER_2000; EUROBAROMETER_2001; EUROBAROMETER_2003; EUROBAROMETER_2005; EUROBAROMETER_2010; EUROBAROMETER_2011; EUROSTAT_1998; EUROSTAT_1999; EUROSTAT_2007; EUROSTAT_2008; EUROSTAT_2009; EUROSTAT_2010; EUROSTAT_2011; EUROSTAT_2012; EUROSTAT_2013; EUROSTAT_2014; EUROSTAT_2015; EUROSTAT_2016; ISSP_1989; ISSP_1992; ISSP_1994; ISSP_1996; ISSP_1997; ISSP_1999; ISSP_2002; ISSP_2003; ISSP_2004; ISSP_2005; ISSP_2006; ISSP_2007; ISSP_2008; ISSP_2011; ISSP_2012; ISSP_2014; ISSP_2015; NORD_IALS_2003; WHS_2003;
**Oman**	CENSUS_1995; CENSUS_2000; DYB_2003;
**Pakistan**	DHS_1991; DHS_2007; DHS_2013; IPUMS_1973; IPUMS_1981; IPUMS_1998; PAKIAL_AND_LIVING_MEASUREMENT_SURVEY_2002; WB_CWIQ_2005; WFS_1975; WHO_WHS_2004;
**Palestine**	ARAB_BAROMETER_2014; DYB_1997; DYB_2007; IPUMS_2007; MICS_2010; MICS_2014;
**Panama**	IPUMS_1960; IPUMS_1970; IPUMS_1980; IPUMS_1990; IPUMS_2000; IPUMS_2010; LATINOBAROMETER_2001; LATINOBAROMETER_2002; LATINOBAROMETER_2003; LATINOBAROMETER_2004; LATINOBAROMETER_2005; LATINOBAROMETER_2006; LATINOBAROMETER_2007; LATINOBAROMETER_2008; LATINOBAROMETER_2009; LATINOBAROMETER_2010; LATINOBAROMETER_2011; LATINOBAROMETER_2013; LATINOBAROMETER_2015; LATINOBAROMETER_2016; LATINOBAROMETER_2017; MICS_2013; WFS_1975;

**Papua New Guinea**	CENSUS_1996; CENSUS_2000; CENSUS_2006; DHS_2007; IPUMS_1980; IPUMS_1990; IPUMS_2000; SURVEY_1997;
**Paraguay**	CDC_RHS_1996; CDC_RHS_1998; CDC_RHS_2004; DHS_1990; IPUMS_1962; IPUMS_1972; IPUMS_1982; IPUMS_1992; IPUMS_2002; LATINOBAROMETER_1998; LATINOBAROMETER_2000; LATINOBAROMETER_2001; LATINOBAROMETER_2002; LATINOBAROMETER_2003; LATINOBAROMETER_2004; LATINOBAROMETER_2005; LATINOBAROMETER_2006; LATINOBAROMETER_2007; LATINOBAROMETER_2008; LATINOBAROMETER_2009; LATINOBAROMETER_2010; LATINOBAROMETER_2011; LATINOBAROMETER_2013; LATINOBAROMETER_2015; LATINOBAROMETER_2016; LATINOBAROMETER_2017; MICS_2016; PRYMANENT_HH_SURVEY_EPH_2015; WFS_1979; WHS_2003;
**Peru**	DHS_1992; DHS_1996; DHS_2000; DHS_2008; DHS_2009; DHS_2010; DHS_2011; DHS_2012; DHS_2013; DHS_2014; IPUMS_1993; IPUMS_2007; LATINOBAROMETER_1998; LATINOBAROMETER_2000; LATINOBAROMETER_2001; LATINOBAROMETER_2002; LATINOBAROMETER_2003; LATINOBAROMETER_2004; LATINOBAROMETER_2005; LATINOBAROMETER_2006; LATINOBAROMETER_2007; LATINOBAROMETER_2009; LATINOBAROMETER_2010; LATINOBAROMETER_2011; LATINOBAROMETER_2013; LATINOBAROMETER_2015; LATINOBAROMETER_2016; LATINOBAROMETER_2017; WFS_1977;
**Philippines**	DHS_1998; DHS_2008; DHS_2013; DHS_2017; GLOBAL_ADULT_TOBACCO_SURVEY_2009; IPUMS_1990; IPUMS_1995; IPUMS_2000; IPUMS_2010; ISSP_1992; ISSP_1994; ISSP_1996; ISSP_1997; ISSP_1998; ISSP_1999; ISSP_2002; ISSP_2003; ISSP_2004; ISSP_2005; ISSP_2006; ISSP_2008; ISSP_2009; ISSP_2010; ISSP_2011; ISSP_2012; ISSP_2014; ISSP_2016; MICS_1999; PHLUAL_POVERTY_SURVEY_2004; WFS_1978; WHS_2003;
**Poland**	EUROBAROMETER_2002; EUROBAROMETER_2004; EUROBAROMETER_2005; EUROBAROMETER_2006; EUROBAROMETER_2007; EUROBAROMETER_2008; EUROBAROMETER_2009; EUROBAROMETER_2010; EUROBAROMETER_2011; EUROBAROMETER_2012; EUROBAROMETER_2013; EUROBAROMETER_2014; EUROBAROMETER_2015; EUROSTAT_1998; EUROSTAT_1999; EUROSTAT_2007; EUROSTAT_2008; EUROSTAT_2009; EUROSTAT_2010; EUROSTAT_2011; EUROSTAT_2012; EUROSTAT_2013; EUROSTAT_2014; EUROSTAT_2015; EUROSTAT_2016; GLOBAL_ADULT_TOBACCO_SURVEY_2010; IPUMS_1978; IPUMS_1988; IPUMS_2002; IPUMS_2011; ISSP_1992; ISSP_1994; ISSP_1996; ISSP_1997; ISSP_1999; ISSP_2002; ISSP_2003; ISSP_2005; ISSP_2008; ISSP_2010; ISSP_2013; ISSP_2015; MCSS_2001; POL_OECD_IALS_1998; SURVEY_ADULT_SKILLS_2012;
**Portugal**	EUROBAROMETER_1990; EUROBAROMETER_1992; EUROBAROMETER_1993; EUROBAROMETER_1994; EUROBAROMETER_1995; EUROBAROMETER_1996; EUROBAROMETER_1997; EUROBAROMETER_1998; EUROBAROMETER_1999; EUROBAROMETER_2000; EUROBAROMETER_2001; EUROBAROMETER_2002; EUROBAROMETER_2003; EUROBAROMETER_2004; EUROBAROMETER_2005; EUROBAROMETER_2006; EUROBAROMETER_2007; EUROBAROMETER_2008; EUROBAROMETER_2009; EUROBAROMETER_2010; EUROBAROMETER_2011; EUROBAROMETER_2012; EUROBAROMETER_2013; EUROBAROMETER_2014; EUROBAROMETER_2015; EUROSTAT_1998; EUROSTAT_2007; EUROSTAT_2008; EUROSTAT_2009; EUROSTAT_2010; EUROSTAT_2011; EUROSTAT_2012; EUROSTAT_2013; EUROSTAT_2014; EUROSTAT_2015; EUROSTAT_2016; IPUMS_1981; IPUMS_1991; IPUMS_2001; IPUMS_2011; ISSP_1997; ISSP_1999; ISSP_2002; ISSP_2003; ISSP_2004; ISSP_2007; ISSP_2009; ISSP_2013; ISSP_2015; WFS_1979; WHS_2003;
**Puerto Rico**	BRFSS_1996; BRFSS_1997; BRFSS_1998; BRFSS_1999; BRFSS_2000; BRFSS_2001; BRFSS_2002; BRFSS_2003; BRFSS_2004; BRFSS_2005; BRFSS_2006; BRFSS_2007; BRFSS_2008; BRFSS_2009; BRFSS_2010; BRFSS_2012; BRFSS_2013; BRFSS_2014; IPUMS_1970; IPUMS_1980; IPUMS_1990; IPUMS_2000; IPUMS_2005; IPUMS_2010; USASS_2015;
**Qatar**	CENSUS_1998; CENSUS_2007; CENSUS_2011; DYB_2004; DYB_2010;
**Romania**	EUROBAROMETER_2004; EUROBAROMETER_2005; EUROBAROMETER_2006; EUROBAROMETER_2007; EUROBAROMETER_2008; EUROBAROMETER_2009; EUROBAROMETER_2010; EUROBAROMETER_2011; EUROBAROMETER_2012; EUROBAROMETER_2013; EUROBAROMETER_2014; EUROBAROMETER_2015; EUROSTAT_1998; EUROSTAT_1999; EUROSTAT_2007; EUROSTAT_2008; EUROSTAT_2009; EUROSTAT_2010; EUROSTAT_2011; EUROSTAT_2012; EUROSTAT_2013; EUROSTAT_2014; EUROSTAT_2015; EUROSTAT_2016; IPUMS_1992; IPUMS_2002; IPUMS_2011; WB_LSMS_1995;
**Russia**	CENSUS_1959; CENSUS_2002; CENSUS_2010; ISSP_1992; ISSP_1994; ISSP_1996; ISSP_1997; ISSP_1998; ISSP_1999; ISSP_2002; ISSP_2003; ISSP_2005; ISSP_2006; ISSP_2007; ISSP_2009; ISSP_2010; ISSP_2011; ISSP_2012; ISSP_2014; ISSP_2015; MCSS_2001; SURVEY_ADULT_SKILLS_2012; WHS_2003;
**Rwanda**	DHS_1992; DHS_2000; DHS_2005; DHS_2011; DHS_2015; IPUMS_2002; IPUMS_2012; MACRO_MIS_2013; MACRO_MIS_2017; MICS_2000; WFS_1983;
**Saint Lucia**	CENSUS_1990; DYB_1991; DYB_2001; IPUMS_1980; IPUMS_1991;
**Saint Vincent and the Grenadines**	CENSUS_1980; CENSUS_1990;
**Sao Tome and Principe**	AFROBAROMETER SURVEY_2015; DHS_2009; MICS_2000; MICS_2014;

**Saudi Arabia**	CENSUS_1996; CENSUS_2001; CENSUS_2005; CENSUS_2007; DYB_2004;
**Senegal**	AFROBAROMETER SURVEY_2014; AFROBAROMETER_2008; AFROBAROMETER_2013; AFROBAROMETER_2017; DHS_1993; DHS_1999; DHS_2005; DHS_2011; DHS_2013; DHS_2014; DHS_2015; DHS_2016; DHS_2017; IPUMS_1988; IPUMS_2002; MACRO_MIS_2006; MACRO_MIS_2009; MICS_2000; WFS_1978;
**Serbia**	EUROBAROMETER_2012; EUROBAROMETER_2013; EUROBAROMETER_2014; EUROBAROMETER_2015; MICS_2010; MICS_2014; WB_LSMS_2003; WB_LSMS_2007;
**Seychelles**	CENSUS_2006; CENSUS_2010; DYB_1997; DYB_2010;
**Sierra Leone**	DHS_2008; DHS_2013; IPUMS_2004; MACRO_MIS_2016; MICS_2000; MICS_2005; MICS_2010; MICS_2017;
**Singapore**	CENSUS_2005; CENSUS_2010; DYB_2000; MCSS_2001;
**Slovakia**	EUROBAROMETER_2004; EUROBAROMETER_2005; EUROBAROMETER_2006; EUROBAROMETER_2007; EUROBAROMETER_2008; EUROBAROMETER_2009; EUROBAROMETER_2010; EUROBAROMETER_2011; EUROBAROMETER_2012; EUROBAROMETER_2013; EUROBAROMETER_2014; EUROBAROMETER_2015; EUROSTAT_1998; EUROSTAT_1999; EUROSTAT_2007; EUROSTAT_2008; EUROSTAT_2009; EUROSTAT_2010; EUROSTAT_2011; EUROSTAT_2012; EUROSTAT_2013; EUROSTAT_2014; EUROSTAT_2015; EUROSTAT_2016; ISSP_1996; ISSP_2000; ISSP_2001; ISSP_2002; ISSP_2004; ISSP_2005; ISSP_2008; ISSP_2009; ISSP_2012; ISSP_2013; ISSP_2014; ISSP_2016; SURVEY_ADULT_SKILLS_2012; WHS_2003;
**Slovenia**	EUROBAROMETER_2004; EUROBAROMETER_2005; EUROBAROMETER_2006; EUROBAROMETER_2007; EUROBAROMETER_2008; EUROBAROMETER_2009; EUROBAROMETER_2010; EUROBAROMETER_2011; EUROBAROMETER_2012; EUROBAROMETER_2013; EUROBAROMETER_2014; EUROBAROMETER_2015; EUROSTAT_1998; EUROSTAT_1999; EUROSTAT_2007; EUROSTAT_2008; EUROSTAT_2009; EUROSTAT_2010; EUROSTAT_2011; EUROSTAT_2012; EUROSTAT_2013; EUROSTAT_2014; EUROSTAT_2015; EUROSTAT_2016; IPUMS_2002; ISSP_1993; ISSP_1994; ISSP_1995; ISSP_1996; ISSP_1997; ISSP_1998; ISSP_2002; ISSP_2003; ISSP_2004; ISSP_2006; ISSP_2009; ISSP_2011; ISSP_2012; ISSP_2013; ISSP_2016;
**Solomon Islands**	CENSUS_2007; CENSUS_2009; CENSUS_2015;
**Somalia**	MICS_2006; MICS_2011;
**South Korea**	ISSP_2003; ISSP_2004; ISSP_2005; ISSP_2006; ISSP_2007; ISSP_2008; ISSP_2009; ISSP_2010; ISSP_2011; ISSP_2012; ISSP_2013; ISSP_2014; KOREAN_LONGITUDIL_STUDY_OF_AGEING_2006; KOREAN_LONGITUDIL_STUDY_OF_AGEING_2010; MCSS_2001; WFS_1974;
**South Sudan**	MICS_2010;
**Spain**	EUROBAROMETER_1990; EUROBAROMETER_1992; EUROBAROMETER_1993; EUROBAROMETER_1994; EUROBAROMETER_1995; EUROBAROMETER_1996; EUROBAROMETER_1997; EUROBAROMETER_1998; EUROBAROMETER_1999; EUROBAROMETER_2000; EUROBAROMETER_2001; EUROBAROMETER_2002; EUROBAROMETER_2003; EUROBAROMETER_2004; EUROBAROMETER_2005; EUROBAROMETER_2006; EUROBAROMETER_2007; EUROBAROMETER_2008; EUROBAROMETER_2009; EUROBAROMETER_2010; EUROBAROMETER_2011; EUROBAROMETER_2012; EUROBAROMETER_2013; EUROBAROMETER_2014; EUROBAROMETER_2015; EUROSTAT_1998; EUROSTAT_1999; EUROSTAT_2007; EUROSTAT_2008; EUROSTAT_2009; EUROSTAT_2010; EUROSTAT_2011; EUROSTAT_2012; EUROSTAT_2013; EUROSTAT_2014; EUROSTAT_2015; EUROSTAT_2016; HH_SURVEY_ALCOHOL_DRUGS_EDADES_2006; HH_SURVEY_ALCOHOL_DRUGS_EDADES_2008; IPUMS_1991; IPUMS_2001; IPUMS_2012; ISSP_1994; ISSP_1996; ISSP_1997; ISSP_1998; ISSP_1999; ISSP_2002; ISSP_2003; ISSP_2004; ISSP_2005; ISSP_2007; ISSP_2008; ISSP_2009; ISSP_2010; ISSP_2012; ISSP_2014; ISSP_2016; MCSS_2001; WHO_WHS_2003;
**Sri Lanka**	CENSUS_2006; DYB_2001; WB_STEP_HH_SURVEY_2012; WFS_1975; WHO_WHS_2003;
**Sudan**	ARAB_LEAGUE_PAPCHILD_1993; DHS_1990; MICS_2000; MICS_2010; MICS_2014; WFS_1978;
**Suriname**	MICS_2000; MICS_2010;
**Swaziland**	AFROBAROMETER SURVEY_2015; AFROBAROMETER_2013; AFROBAROMETER_2018; CENSUS_2007; DHS_2007; MICS_2000; MICS_2010; MICS_2014; WHS_2003;
**Sweden**	CENSUS_1985; CENSUS_1986; CENSUS_1987; CENSUS_1988; CENSUS_1989; CENSUS_1990; CENSUS_1991; CENSUS_1992; CENSUS_1993; CENSUS_1994; CENSUS_1995; CENSUS_1996; CENSUS_1997; CENSUS_1998; CENSUS_1999; CENSUS_2000; CENSUS_2001; CENSUS_2002; CENSUS_2003; CENSUS_2004; CENSUS_2005; CENSUS_2006; CENSUS_2007; CENSUS_2008; CENSUS_2009; CENSUS_2010; CENSUS_2011; CENSUS_2012; CENSUS_2013; CENSUS_2014; CENSUS_2015; EUROBAROMETER_1995; EUROBAROMETER_1996; EUROBAROMETER_1997; EUROBAROMETER_1998; EUROBAROMETER_1999; EUROBAROMETER_2000; EUROBAROMETER_2001; EUROBAROMETER_2002; EUROBAROMETER_2003; EUROBAROMETER_2004; EUROBAROMETER_2005; EUROBAROMETER_2006; EUROBAROMETER_2007; EUROBAROMETER_2008;

	EUROBAROMETER_2009; EUROBAROMETER_2010; EUROBAROMETER_2011; EUROBAROMETER_2012; EUROBAROMETER_2013; EUROBAROMETER_2014; EUROBAROMETER_2015; EUROSTAT_1998; EUROSTAT_1999; EUROSTAT_2007; EUROSTAT_2008; EUROSTAT_2009; EUROSTAT_2010; EUROSTAT_2011; EUROSTAT_2012; EUROSTAT_2013; EUROSTAT_2014; EUROSTAT_2015; EUROSTAT_2016; ISSP_1994; ISSP_1996; ISSP_1997; ISSP_1998; ISSP_1999; ISSP_2002; ISSP_2003; ISSP_2004; ISSP_2006; ISSP_2007; ISSP_2008; ISSP_2009; ISSP_2010; ISSP_2011; ISSP_2012; ISSP_2013; ISSP_2014; ISSP_2017; MCSS_2001; SWEITOR_SURVEY_2010; SWEITOR_SURVEY_2012; WHS_2003;
**Switzerland**	CHED_IALS_2003; EUROBAROMETER_2005; EUROBAROMETER_2010; EUROSTAT_1998; EUROSTAT_1999; EUROSTAT_2007; EUROSTAT_2008; EUROSTAT_2009; EUROSTAT_2010; EUROSTAT_2011; EUROSTAT_2012; EUROSTAT_2013; EUROSTAT_2014; EUROSTAT_2015; EUROSTAT_2016; IPUMS_1970; IPUMS_1980; IPUMS_1990; IPUMS_2000; ISSP_1998; ISSP_1999; ISSP_2002; ISSP_2003; ISSP_2005; ISSP_2007; ISSP_2009; ISSP_2011; ISSP_2013; ISSP_2015; MCSS_2001;
**Syria**	ARAB_LEAGUE_PAPCHILD_1993; MICS_2006; WFS_1978;
**Taiwan**	ISSP_2002; ISSP_2003; ISSP_2004; ISSP_2005; ISSP_2006; ISSP_2007; ISSP_2009; ISSP_2010; ISSP_2012; ISSP_2013; ISSP_2014; ISSP_2015; TWNOR_FORCE_SURVEY_1978;
**Tajikistan**	DHS_2012; DHS_2017; DYB_1989; DYB_2000; DYB_2010; MICS_2000; MICS_2005; WB_LSMS_1999; WB_LSMS_2003; WB_LSMS_2007; WB_LSMS_2009;
**Tanzania**	AFROBAROMETER SURVEY_2014; AFROBAROMETER_2005; AFROBAROMETER_2012; AFROBAROMETER_2017; DHS_1996; DHS_1999; DHS_2005; DHS_2010; DHS_2016; IPUMS_1988; IPUMS_2002; IPUMS_2012; MACRO_AIS_2004; MACRO_AIS_2008; MACRO_AIS_2012; MACRO_KAP_1994;
**Thailand**	DHS_1987; IPUMS_1970; IPUMS_1980; IPUMS_1990; IPUMS_2000; MICS_2006; MICS_2012; MICS_2016; WFS_1975;
**The Bahamas**	CENSUS_1990; CENSUS_2000; DYB_1990; DYB_2010;
**The Gambia**	CENSUS_2013; DHS_2013; DYB_1993; MICS_2000; MICS_2010;
**Timor-Leste**	CENSUS_2003; CENSUS_2009; DHS_2003; DHS_2010; DHS_2016; WB_LSMS_2008;
**Togo**	AFROBAROMETER_2012; DHS_1998; DHS_2014; MACRO_MIS_2017; MICS_2000; MICS_2006; MICS_2010;
**Tonga**	CENSUS_1976; DYB_1986; DYB_1996; DYB_2006;
**Trinidad and Tobago**	CENSUS_1990; DYB_1990; DYB_2011; IPUMS_2000; IPUMS_2011; MICS_2000; MICS_2006; MICS_2011; TIOL_SURVEY_PRIMARY_SCHOOL_CHILDREN_2004; SURVEY_2011; TTO_TIOL_SURVEY_FOR_DIABETES_AND_HEALTH_2001; WFS_1977;
**Tunisia**	AFROBAROMETER SURVEY_2015; AFROBAROMETER_2013; AFROBAROMETER_2018; ARAB_BAROMETER_2014; CENSUS_2000; DYB_1994; MICS_2012; WFS_1978; WHS_2003;
**Turkey**	DHS_1993; DHS_1998; DHS_2004; EUROBAROMETER_2004; EUROBAROMETER_2005; EUROBAROMETER_2006; EUROBAROMETER_2007; EUROBAROMETER_2008; EUROBAROMETER_2009; EUROBAROMETER_2010; EUROBAROMETER_2011; EUROBAROMETER_2012; EUROBAROMETER_2013; EUROBAROMETER_2014; EUROBAROMETER_2015; IPUMS_2000; ISSP_2009; ISSP_2010; ISSP_2012; ISSP_2013; ISSP_2014; ISSP_2015; TUROGRAPHIC_HEALTH_SURVEY_2008; WFS_1978; WHS_2003;
**Turkmenistan**	CENSUS_2000; MICS_2006; MICS_2016;
**Uganda**	AFROBAROMETER SURVEY_2015; AFROBAROMETER_2012; AFROBAROMETER_2017; DHS_1995; DHS_2001; DHS_2006; DHS_2011; DHS_2016; IPUMS_1991; IPUMS_2002; MACRO_AIS_2005; MACRO_AIS_2011; MACRO_MIS_2015; WB_LSMS_2010;
**Ukraine**	CENSUS_2001; DHS_2007; GLOBAL_ADULT_TOBACCO_SURVEY_2010; HEALTH_IN_TIMES_OF_TRANSITION_HITT_CIS_2010; IPUMS_2001; ISSP_2008; ISSP_2009; MCSS_2001; MICS_2005; MICS_2012; UKRAINIAN_LONGITUDIL_MONITORING_SURVEY_2004; WB_STEP_HH_SURVEY_2013; WHS_2003;
**United Arab Emirates**	CENSUS_1995; CENSUS_2005; WHO_WHS_2003;
**United Kingdom**	CENSUS_2011; EUROBAROMETER_1990; EUROBAROMETER_1992; EUROBAROMETER_1993; EUROBAROMETER_1994; EUROBAROMETER_1995; EUROBAROMETER_1996; EUROBAROMETER_1997; EUROBAROMETER_1998; EUROBAROMETER_1999; EUROBAROMETER_2000; EUROBAROMETER_2001; EUROBAROMETER_2002; EUROBAROMETER_2003; EUROBAROMETER_2004; EUROBAROMETER_2005; EUROBAROMETER_2006; EUROBAROMETER_2007; EUROBAROMETER_2008; EUROBAROMETER_2009; EUROBAROMETER_2010; EUROBAROMETER_2011; EUROBAROMETER_2012; EUROBAROMETER_2013; EUROBAROMETER_2014; EUROBAROMETER_2015; EUROSTAT_1999; EUROSTAT_2007; EUROSTAT_2008; EUROSTAT_2009; EUROSTAT_2010; EUROSTAT_2011; EUROSTAT_2012; EUROSTAT_2013; EUROSTAT_2014; EUROSTAT_2015; EUROSTAT_2016; MCSS_2001; SURVEY_ADULT_SKILLS_2012; UKGHS_1972; UKGHS_1973; UKGHS_1974; UKGHS_1975; UKGHS_1976; UKGHS_1977; UKGHS_1978; UKGHS_1980; UKGHS_1981; UKGHS_1982; UKGHS_1983; UKGHS_1984; UKGHS_1985; UKGHS_1986;

**United States**	BRFSS_1984; BRFSS_1985; BRFSS_1986; BRFSS_1987; BRFSS_1988; BRFSS_1989; BRFSS_1990; BRFSS_1991; BRFSS_1992; BRFSS_1993; BRFSS_1994; BRFSS_1995; BRFSS_1996; BRFSS_1997; BRFSS_1998; BRFSS_1999; BRFSS_2000; BRFSS_2001; BRFSS_2002; BRFSS_2003; BRFSS_2004; BRFSS_2005; BRFSS_2006; BRFSS_2007; BRFSS_2008; BRFSS_2009; BRFSS_2010; BRFSS_2012; BRFSS_2013; BRFSS_2014; IPUMS_1960; IPUMS_1970; IPUMS_1980; IPUMS_1990; IPUMS_2000; IPUMS_2005; IPUMS_2010; IPUMS_2015; ISSP_1989; ISSP_1994; ISSP_1996; ISSP_1998; ISSP_2000; ISSP_2002; ISSP_2004; ISSP_2005; ISSP_2006; ISSP_2008; ISSP_2010; ISSP_2012; ISSP_2014; ISSP_2016; MCSS_2001; USAIOL_COMORBIDITY_SURVEY_1992; USALABORATIVE_PSYCHIATRIC_EPIDEMIOLOGY_SURVEYS_2003; USASS_2015;
**Uruguay**	CONTINUOUS_HOUSEHOLD_SURVEY_2015; IPUMS_1963; IPUMS_1975; IPUMS_1985; IPUMS_1996; IPUMS_2006; IPUMS_2011; ISSP_2004; ISSP_2006; ISSP_2008; LATINOBAROMETER_1998; LATINOBAROMETER_2000; LATINOBAROMETER_2001; LATINOBAROMETER_2002; LATINOBAROMETER_2003; LATINOBAROMETER_2004; LATINOBAROMETER_2005; LATINOBAROMETER_2006; LATINOBAROMETER_2007; LATINOBAROMETER_2008; LATINOBAROMETER_2009; LATINOBAROMETER_2011; LATINOBAROMETER_2013; LATINOBAROMETER_2015; LATINOBAROMETER_2016; LATINOBAROMETER_2017; MICS_2013; WHS_2003;
**Uzbekistan**	DHS_1996; DHS_2002; MICS_2000; MICS_2006;
**Venezuela**	IPUMS_1971; IPUMS_1981; IPUMS_1990; IPUMS_2001; ISSP_2003; ISSP_2008; ISSP_2010; ISSP_2013; ISSP_2015; LATINOBAROMETER_1998; LATINOBAROMETER_2000; LATINOBAROMETER_2001; LATINOBAROMETER_2002; LATINOBAROMETER_2003; LATINOBAROMETER_2004; LATINOBAROMETER_2005; LATINOBAROMETER_2006; LATINOBAROMETER_2007; LATINOBAROMETER_2008; LATINOBAROMETER_2009; LATINOBAROMETER_2011; LATINOBAROMETER_2013; LATINOBAROMETER_2015; LATINOBAROMETER_2016; LATINOBAROMETER_2017; MCSS_2001; MICS_2000; WFS_1977;
**Vietnam**	DHS_1997; DHS_2002; IPUMS_1989; IPUMS_1999; MACRO_AIS_2005; MICS_2000; MICS_2006; MICS_2011; MICS_2014; WB_LSMS_2002; WHS_2003;
**Virgin Islands, U.S.**	BRFSS_2001; BRFSS_2002; BRFSS_2003; BRFSS_2004; BRFSS_2005; BRFSS_2006; BRFSS_2007; BRFSS_2008; BRFSS_2009; BRFSS_2010;

Supplement Table 2. Country Specific Data Sources by Year and Data Provider

All country-years of data are listed by data provider.

Location Name	Region Name	Location Gold Standard	Region Gold Standard
**Afghanistan**	North Africa and Middle East	DHS	DHS
**Albania**	Central Europe	DHS	IPUMS
**Algeria**	North Africa and Middle East	MICS	DHS
**American Samoa**	Oceania	NONE	NONE
**Andorra**	Western Europe	NONE	NONE
**Angola**	Central Sub-Saharan Africa	DHS	DHS
**Antigua and Barbuda**	Caribbean	NONE	IPUMS
**Argentina**	Southern Latin America	IPUMS	IPUMS
**Armenia**	Central Asia	DHS	DHS
**Australia**	Australasia	CENSUS	NONE
**Austria**	Western Europe	IPUMS	IPUMS
**Azerbaijan**	Central Asia	DHS	DHS
**Bahrain**	North Africa and Middle East	CENSUS	DHS
**Bangladesh**	South Asia	DHS	DHS
**Barbados**	Caribbean	NONE	IPUMS
**Belarus**	Eastern Europe	IPUMS	IPUMS
**Belgium**	Western Europe	NONE	IPUMS
**Belize**	Caribbean	NONE	IPUMS

**Benin**	Western Sub-Saharan Africa	IPUMS	DHS
**Bermuda**	Caribbean	NONE	IPUMS
**Bhutan**	South Asia	MICS	DHS
**Bolivia**	Andean Latin America	DHS	DHS
**Bosnia and Herzegovina**	Central Europe	MICS	IPUMS
**Botswana**	Southern Sub-Saharan Africa	IPUMS	DHS
**Brazil**	Tropical Latin America	IPUMS	IPUMS
**Brunei**	High-income Asia Pacific	CENSUS	NONE
**Bulgaria**	Central Europe	NONE	IPUMS
**Burkina Faso**	Western Sub-Saharan Africa	IPUMS	DHS
**Burundi**	Eastern Sub-Saharan Africa	DHS	DHS
**Cambodia**	Southeast Asia	DHS	DHS
**Cameroon**	Western Sub-Saharan Africa	DHS	DHS
**Canada**	High-income North America	IPUMS	IPUMS
**Cape Verde**	Western Sub-Saharan Africa	DHS	DHS
**Central African Republic**	Central Sub-Saharan Africa	DHS	DHS
**Chad**	Western Sub-Saharan Africa	DHS	DHS
**Chile**	Southern Latin America	IPUMS	IPUMS
**China**	East Asia	IPUMS	IPUMS
**Colombia**	Central Latin America	IPUMS	IPUMS
**Comoros**	Eastern Sub-Saharan Africa	DHS	DHS
**Congo**	Central Sub-Saharan Africa	DHS	DHS
**Costa Rica**	Central Latin America	IPUMS	IPUMS
**Cote d'Ivoire**	Western Sub-Saharan Africa	DHS	DHS
**Croatia**	Central Europe	NONE	IPUMS
**Cuba**	Caribbean	IPUMS	IPUMS
**Cyprus**	Western Europe	NONE	IPUMS
**Czech Republic**	Central Europe	NONE	IPUMS
**Democratic Republic of the Congo**	Central Sub-Saharan Africa	DHS	DHS
**Denmark**	Western Europe	NONE	IPUMS
**Djibouti**	Eastern Sub-Saharan Africa	MICS	DHS
**Dominica**	Caribbean	NONE	IPUMS
**Dominican Republic**	Caribbean	IPUMS	IPUMS
**Ecuador**	Andean Latin America	IPUMS	DHS
**Egypt**	North Africa and Middle East	DHS	DHS
**El Salvador**	Central Latin America	IPUMS	IPUMS
**Equatorial Guinea**	Central Sub-Saharan Africa	MICS	DHS
**Eritrea**	Eastern Sub-Saharan Africa	DHS	DHS
**Estonia**	Eastern Europe	NONE	IPUMS
**Ethiopia**	Eastern Sub-Saharan Africa	DHS	DHS
**Federated States of Micronesia**	Oceania	CENSUS	IPUMS
**Fiji**	Oceania	IPUMS	IPUMS

**Finland**	Western Europe	NONE	IPUMS
**France**	Western Europe	IPUMS	IPUMS
**Gabon**	Central Sub-Saharan Africa	DHS	DHS
**Georgia**	Central Asia	NONE	DHS
**Germany**	Western Europe	NONE	IPUMS
**Ghana**	Western Sub-Saharan Africa	DHS	DHS
**Greece**	Western Europe	IPUMS	IPUMS
**Greenland**	High-income North America	NONE	IPUMS
**Grenada**	Caribbean	NONE	IPUMS
**Guam**	Oceania	NONE	NONE
**Guatemala**	Central Latin America	DHS	IPUMS
**Guinea**	Western Sub-Saharan Africa	DHS	DHS
**Guinea-Bissau**	Western Sub-Saharan Africa	MICS	DHS
**Guyana**	Caribbean	DHS	IPUMS
**Haiti**	Caribbean	DHS	IPUMS
**Honduras**	Central Latin America	IPUMS	IPUMS
**Hungary**	Central Europe	IPUMS	IPUMS
**Iceland**	Western Europe	NONE	IPUMS
**India**	South Asia	DHS	DHS
**Indonesia**	Southeast Asia	DHS	DHS
**Iran**	North Africa and Middle East	IPUMS	DHS
**Iraq**	North Africa and Middle East	IPUMS	DHS
**Ireland**	Western Europe	IPUMS	IPUMS
**Israel**	Western Europe	IPUMS	IPUMS
**Italy**	Western Europe	IPUMS	IPUMS
**Jamaica**	Caribbean	IPUMS	IPUMS
**Japan**	High-income Asia Pacific	NONE	NONE
**Jordan**	North Africa and Middle East	DHS	DHS
**Kazakhstan**	Central Asia	DHS	DHS
**Kenya**	Eastern Sub-Saharan Africa	DHS	DHS
**Kiribati**	Oceania	CENSUS	IPUMS
**Kuwait**	North Africa and Middle East	NONE	DHS
**Kyrgyzstan**	Central Asia	DHS	DHS
**Laos**	Southeast Asia	CENSUS	DHS
**Latvia**	Eastern Europe	CENSUS	IPUMS
**Lebanon**	North Africa and Middle East	NONE	DHS
**Lesotho**	Southern Sub-Saharan Africa	DHS	DHS
**Liberia**	Western Sub-Saharan Africa	IPUMS	DHS
**Libya**	North Africa and Middle East	NONE	DHS
**Lithuania**	Eastern Europe	NONE	IPUMS
**Luxembourg**	Western Europe	NONE	IPUMS
**Macedonia**	Central Europe	NONE	IPUMS

**Madagascar**	Eastern Sub-Saharan Africa	DHS	DHS
**Malawi**	Eastern Sub-Saharan Africa	DHS	DHS
**Malaysia**	Southeast Asia	IPUMS	DHS
**Maldives**	Southeast Asia	DHS	DHS
**Mali**	Western Sub-Saharan Africa	DHS	DHS
**Malta**	Western Europe	NONE	IPUMS
**Marshall Islands**	Oceania	NONE	NONE
**Mauritania**	Western Sub-Saharan Africa	MICS	DHS
**Mauritius**	Southeast Asia	CENSUS	DHS
**Mauritius**	Southeast Asia	NONE	DHS
**Mexico**	Central Latin America	IPUMS	IPUMS
**Moldova**	Eastern Europe	DHS	IPUMS
**Mongolia**	Central Asia	IPUMS	DHS
**Montenegro**	Central Europe	NONE	IPUMS
**Morocco**	North Africa and Middle East	DHS	DHS
**Mozambique**	Eastern Sub-Saharan Africa	DHS	DHS
**Myanmar**	Southeast Asia	DHS	DHS
**Namibia**	Southern Sub-Saharan Africa	DHS	DHS
**Nepal**	South Asia	DHS	DHS
**Netherlands**	Western Europe	IPUMS	IPUMS
**New Zealand**	Australasia	CENSUS	NONE
**Nicaragua**	Central Latin America	IPUMS	IPUMS
**Niger**	Western Sub-Saharan Africa	DHS	DHS
**Nigeria**	Western Sub-Saharan Africa	DHS	DHS
**North Korea**	East Asia	NONE	IPUMS
**Northern Mariana Islands**	Oceania	NONE	NONE
**Norway**	Western Europe	NONE	IPUMS
**Oman**	North Africa and Middle East	CENSUS	DHS
**Pakistan**	South Asia	DHS	DHS
**Palestine**	North Africa and Middle East	IPUMS	DHS
**Panama**	Central Latin America	IPUMS	IPUMS
**Papua New Guinea**	Oceania	IPUMS	IPUMS
**Paraguay**	Tropical Latin America	IPUMS	IPUMS
**Peru**	Andean Latin America	DHS	DHS
**Philippines**	Southeast Asia	DHS	DHS
**Poland**	Central Europe	IPUMS	IPUMS
**Portugal**	Western Europe	IPUMS	IPUMS
**Puerto Rico**	Caribbean	IPUMS	IPUMS
**Qatar**	North Africa and Middle East	NONE	DHS
**Romania**	Central Europe	IPUMS	IPUMS
**Russia**	Eastern Europe	CENSUS	IPUMS
**Rwanda**	Eastern Sub-Saharan Africa	DHS	DHS

**Saint Lucia**	Caribbean	IPUMS	IPUMS
**Saint Vincent and the Grenadines**	Caribbean	NONE	IPUMS
**Samoa**	Oceania	NONE	NONE
**Sao Tome and Principe**	Western Sub-Saharan Africa	DHS	DHS
**Saudi Arabia**	North Africa and Middle East	NONE	DHS
**Senegal**	Western Sub-Saharan Africa	DHS	DHS
**Serbia**	Central Europe	NONE	IPUMS
**Seychelles**	Southeast Asia	CENSUS	DHS
**Sierra Leone**	Western Sub-Saharan Africa	DHS	DHS
**Singapore**	High-income Asia Pacific	NONE	NONE
**Slovakia**	Central Europe	NONE	IPUMS
**Slovenia**	Central Europe	IPUMS	IPUMS
**Solomon Islands**	Oceania	NONE	IPUMS
**Somalia**	Eastern Sub-Saharan Africa	MICS	DHS
**South Africa**	Southern Sub-Saharan Africa	DHS	DHS
**South Korea**	High-income Asia Pacific	NONE	NONE
**South Sudan**	Eastern Sub-Saharan Africa	NONE	DHS
**Spain**	Western Europe	IPUMS	IPUMS
**Sri Lanka**	Southeast Asia	CENSUS	DHS
**Sudan**	North Africa and Middle East	NONE	DHS
**Suriname**	Caribbean	MICS	IPUMS
**Swaziland**	Southern Sub-Saharan Africa	DHS	DHS
**Sweden**	Western Europe	CENSUS	IPUMS
**Switzerland**	Western Europe	IPUMS	IPUMS
**Syria**	North Africa and Middle East	MICS	DHS
**Taiwan**	East Asia	NONE	IPUMS
**Tajikistan**	Central Asia	DHS	DHS
**Tanzania**	Eastern Sub-Saharan Africa	DHS	DHS
**Thailand**	Southeast Asia	DHS	DHS
**The Bahamas**	Caribbean	CENSUS	IPUMS
**The Gambia**	Western Sub-Saharan Africa	DHS	DHS
**Timor-Leste**	Southeast Asia	DHS	DHS
**Togo**	Western Sub-Saharan Africa	DHS	DHS
**Tonga**	Oceania	NONE	IPUMS
**Trinidad and Tobago**	Caribbean	IPUMS	IPUMS
**Tunisia**	North Africa and Middle East	CENSUS	DHS
**Turkey**	North Africa and Middle East	DHS	DHS
**Turkmenistan**	Central Asia	CENSUS	DHS
**Uganda**	Eastern Sub-Saharan Africa	DHS	DHS
**Ukraine**	Eastern Europe	DHS	IPUMS
**United Arab Emirates**	North Africa and Middle East	NONE	DHS
**United Kingdom**	Western Europe	CENSUS	IPUMS

**United States**	High-income North America	IPUMS	IPUMS
**Uruguay**	Southern Latin America	CENSUS	IPUMS
**Uzbekistan**	Central Asia	DHS	DHS
**Vanuatu**	Oceania	NONE	IPUMS
**Venezuela**	Central Latin America	IPUMS	IPUMS
**Vietnam**	Southeast Asia	DHS	DHS
**Virgin Islands, U.S.**	Caribbean	NONE	IPUMS
**Yemen**	North Africa and Middle East	DHS	DHS
**Zambia**	Eastern Sub-Saharan Africa	DHS	DHS
**Zimbabwe**	Southern Sub-Saharan Africa	DHS	DHS

Supplement Table 3. Country- and Region-Specific Gold Standard Data Providers

The data providers considered to be the “gold standard” for each country and region are shown for all countries included in the study.

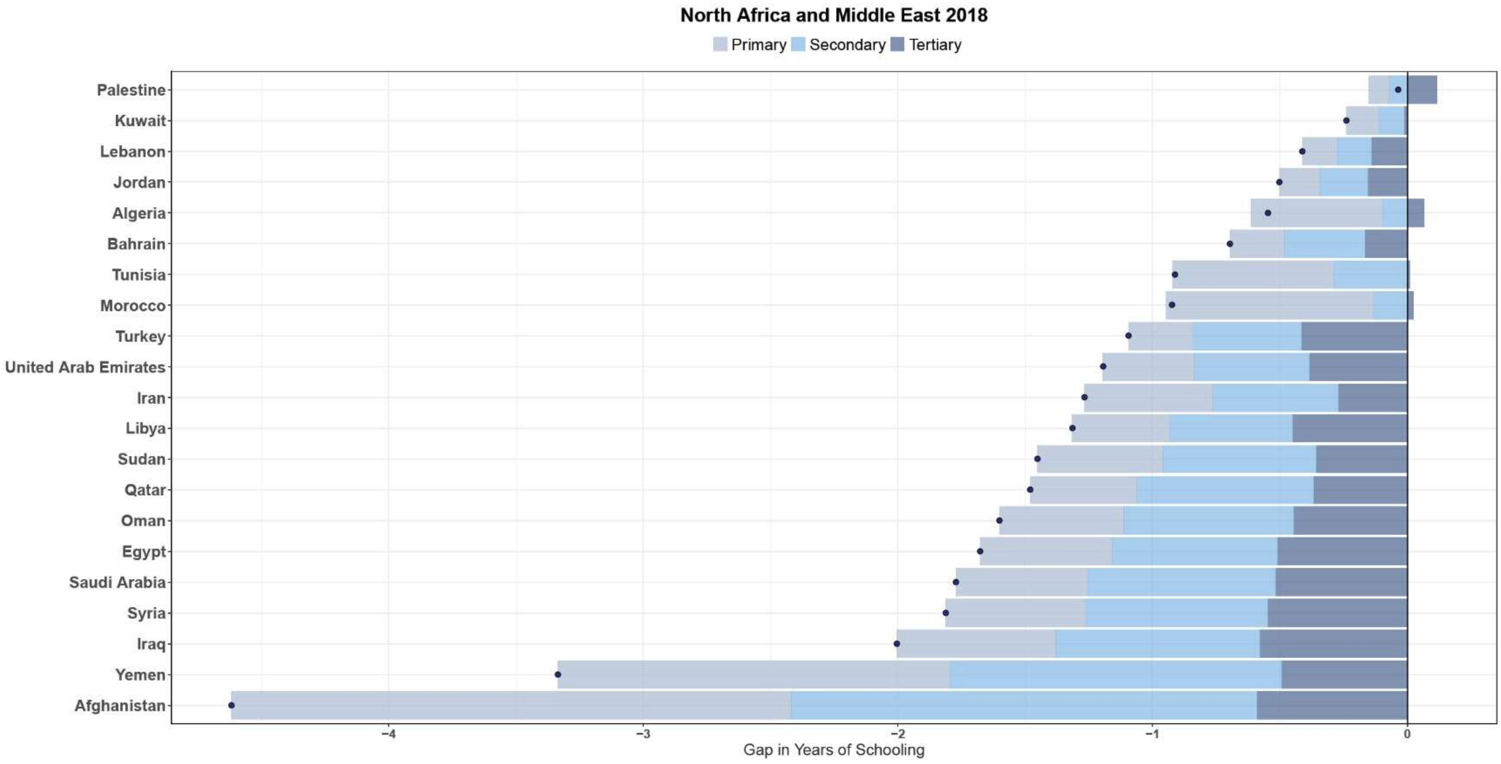
North Africa and Middle East 2018
Primary
Secondary
Tertiary
Palestine
Kuwait
Lebanon
Jordan
Algeria
Bahrain
Tunisia
Morocco
Turkey
United Arab Emirates
Iran
Libya
Sudan
Qatar
Oman
Egypt
Saudi Arabia
Syria
Iraq
Yemen
Afghanistan
−4
−3
−2
−1
0
Gap in Years of Schooling

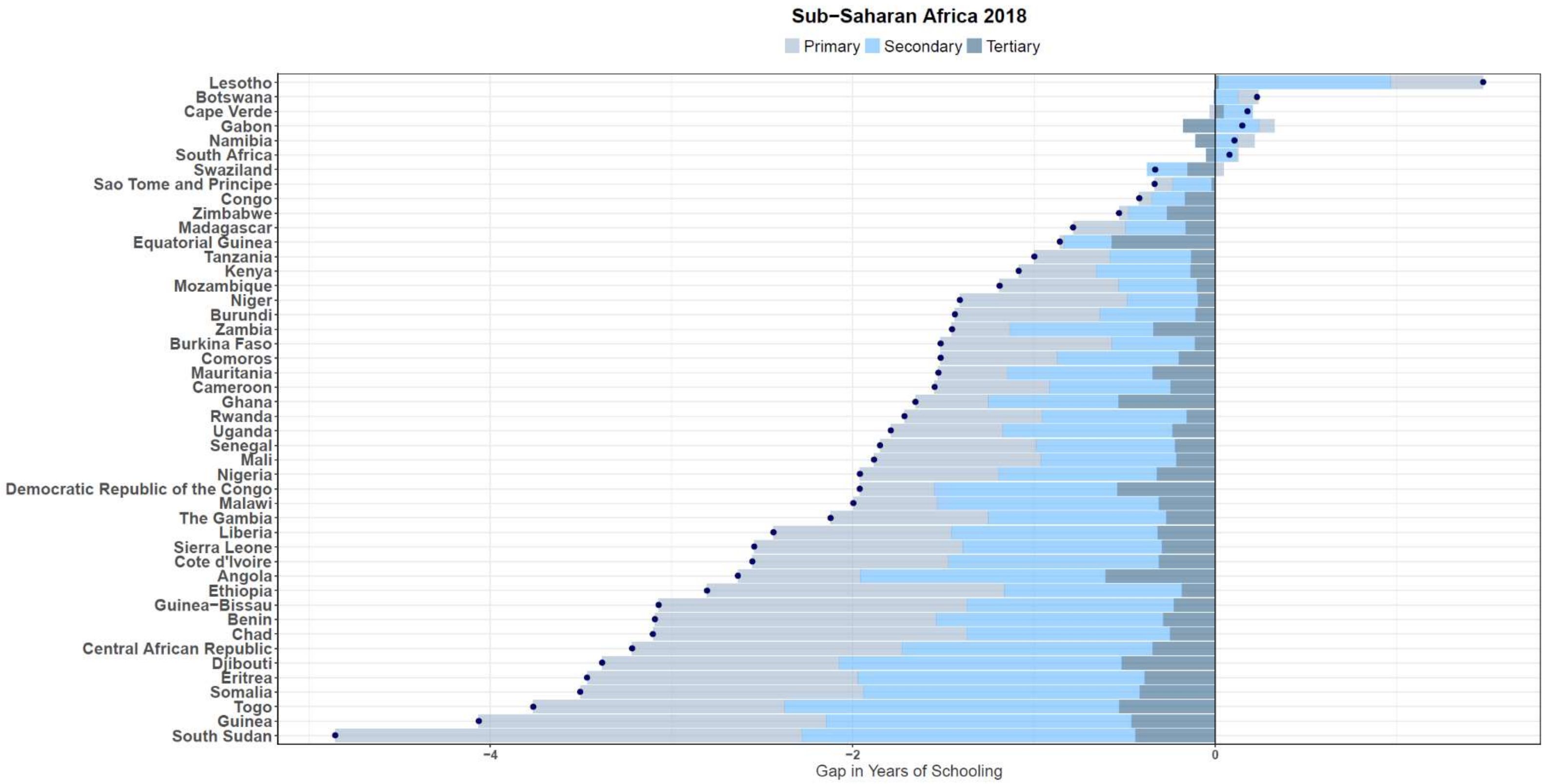
Sub−Saharan Africa 2018
Primary
Secondary
Tertiary
Lesotho
Botswana
Cape Verde
Gabon
Namibia
South Africa
Swaziland
Sao Tome and Principe
Congo
Zimbabwe
Madagascar
Equatorial Guinea
Tanzania
Kenya
Mozambique
Niger
Burundi
Zambia
Burkina Faso
Comoros
Mauritania
Cameroon
Ghana
Rwanda
Uganda
Senegal
Mali
Nigeria
Democratic Republic of the Congo
Malawi
The Gambia
Liberia
Sierra Leone
Cote d'Ivoire
Angola
Ethiopia
Guinea−Bissau
Benin
Chad
Central African Republic
Djibouti
Eritrea
Somalia
Togo
Guinea
South Sudan
−4
−2
0
Gap in Years of Schooling

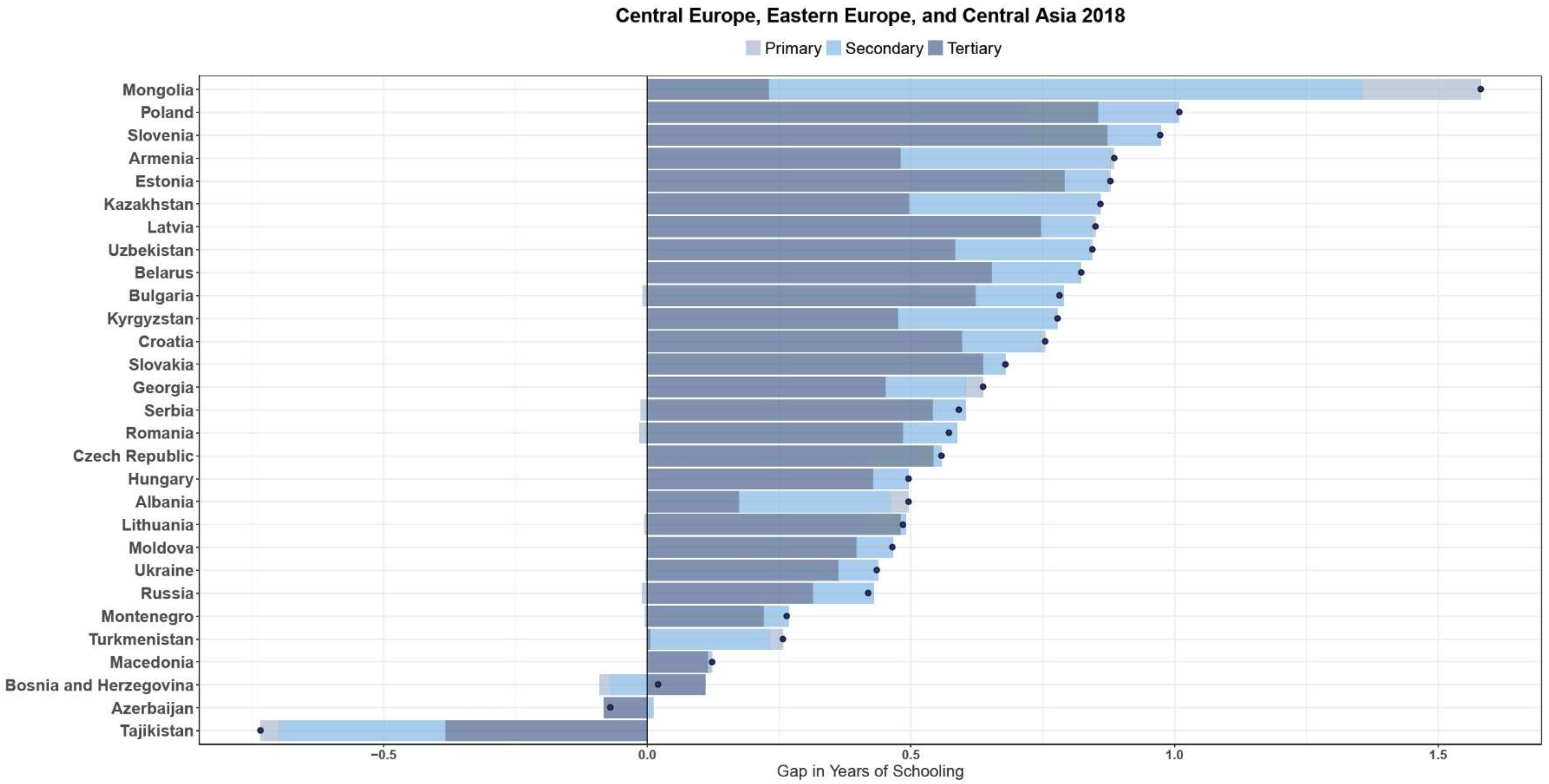
Central Europe, Eastern Europe, and Central Asia 2018
Primary
Secondary
Tertiary
Mongolia
Poland
Slovenia
Armenia
Estonia
Kazakhstan
Latvia
Uzbekistan
Belarus
Bulgaria
Kyrgyzstan
Croatia
Slovakia
Georgia
Serbia
Romania
Czech Republic
Hungary
Albania
Lithuania
Moldova
Ukraine
Russia
Montenegro
Turkmenistan
Macedonia
Bosnia and Herzegovina
Azerbaijan
Tajikistan
−0.5
0.0
0.5
1.0
1.5
Gap in Years of Schooling

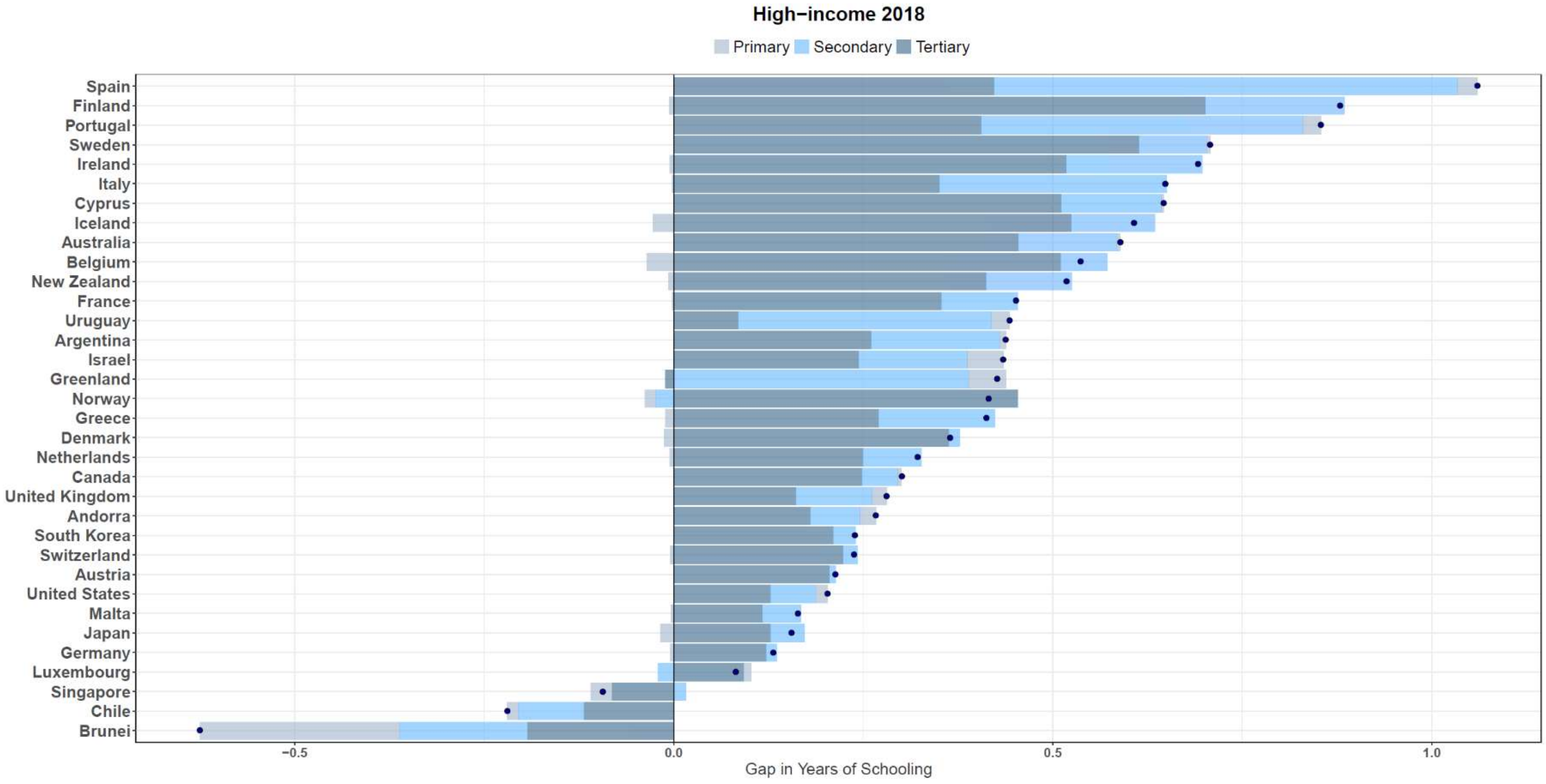
High−income 2018
Primary
Secondary
Tertiary
Spain
Finland
Portugal
Sweden
Ireland
Italy
Cyprus
Iceland
Australia
Belgium
New Zealand
France
Uruguay
Argentina
Israel
Greenland
Norway
Greece
Denmark
Netherlands
Canada
United Kingdom
Andorra
South Korea
Switzerland
Austria
United States
Malta
Japan
Germany
Luxembourg
Singapore
Chile
Brunei
−0.5
0.0
0.5
1.0
Gap in Years of Schooling

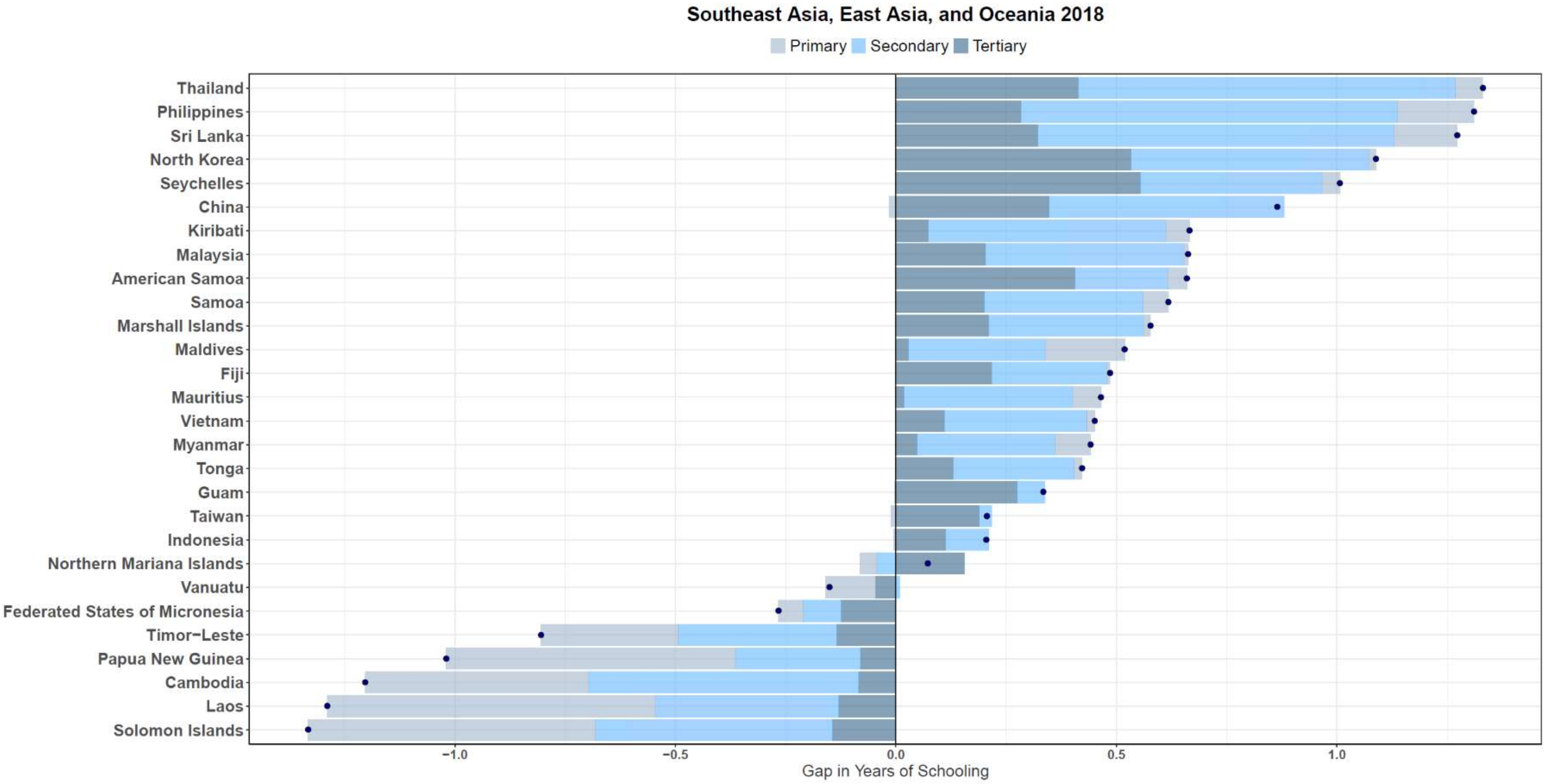
Southeast Asia, East Asia, and Oceania 2018
Primary
Secondary
Tertiary
Thailand
Philippines
Sri Lanka
North Korea
Seychelles
China
Kiribati
Malaysia
American Samoa
Samoa
Marshall Islands
Maldives
Fiji
Mauritius
Vietnam
Myanmar
Tonga
Guam
Taiwan
Indonesia
Northern Mariana Islands
Vanuatu
Federated States of Micronesia
Timor−Leste
Papua New Guinea
Cambodia
Laos
Solomon Islands
−1.0
−0.5
0.0
0.5
1.0
Gap in Years of Schooling

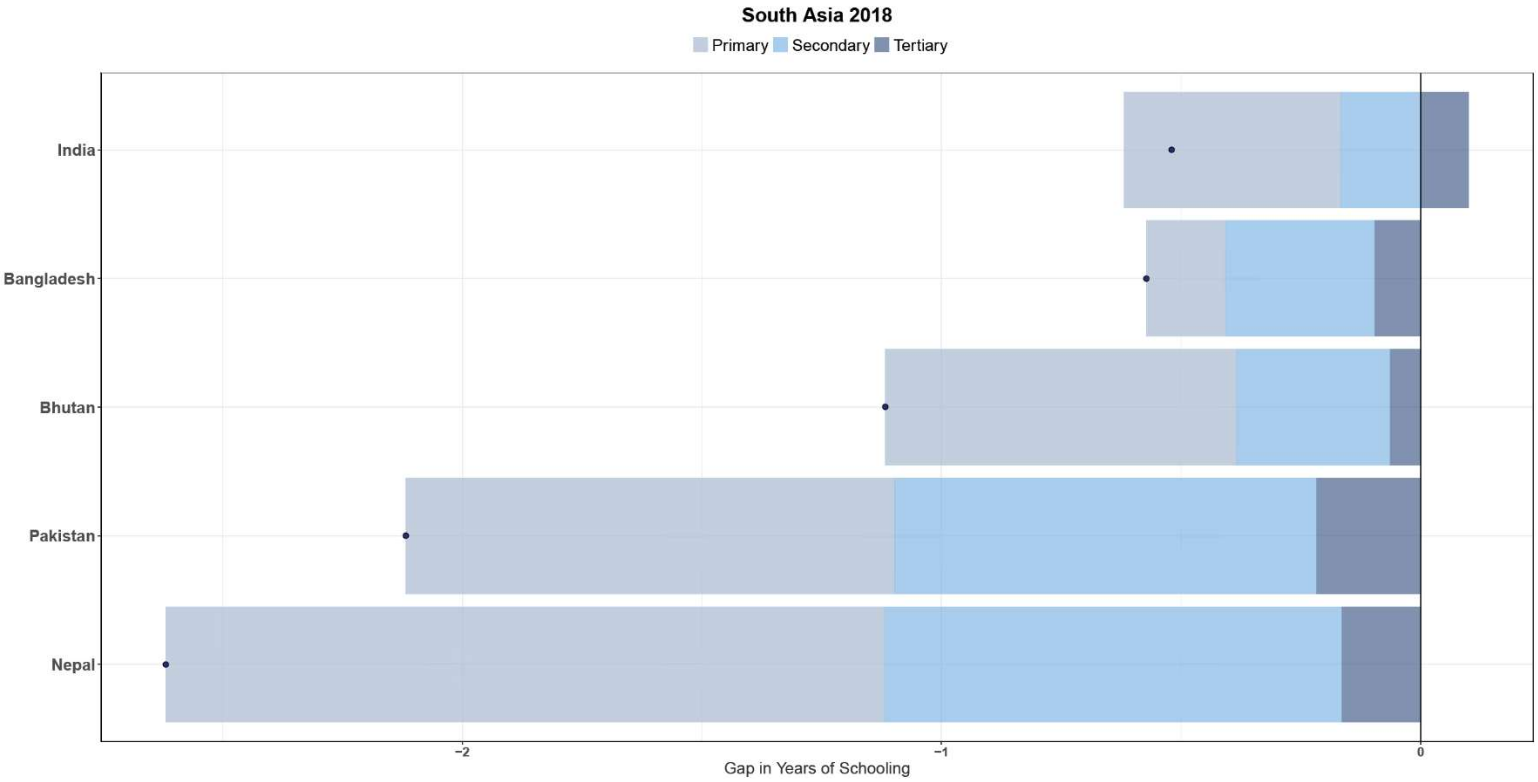
South Asia 2018
Primary
Secondary
Tertiary
India
Bangladesh
Bhutan
Pakistan
Nepal
−2
−1
0
Gap in Years of Schooling

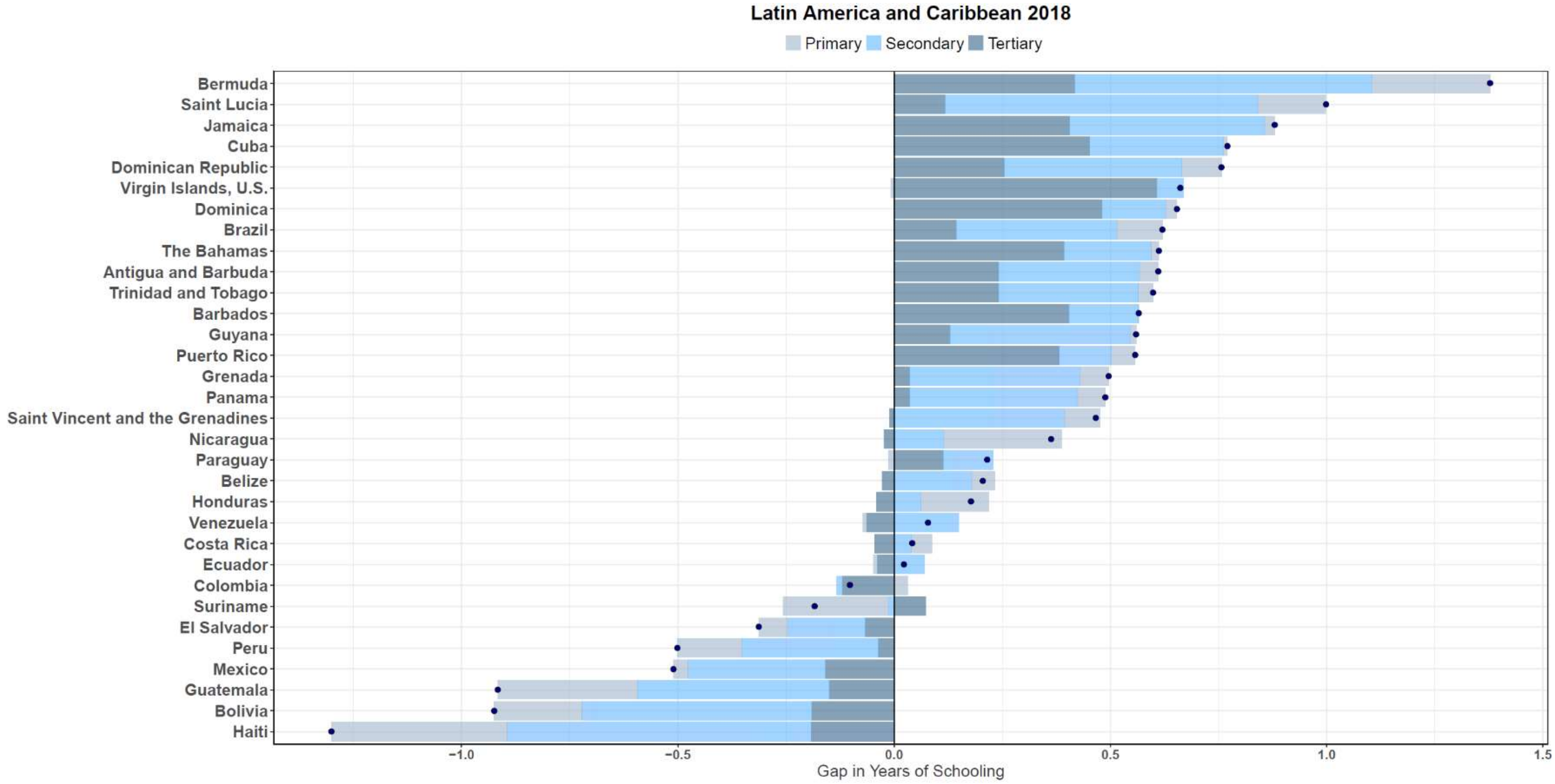

Supplemental Figure 14. Country Specific Gender Gaps in Years of Schooling

The gender gap in years of schooling is represented with a black dot, for individuals age 25-29 in 2018 at the national level, separate by regional group. A positive number indicates additional schooling for women relative to their male counterparts. Grey, light blue, and dark blue bars represent the contributions of primary, secondary, and tertiary schooling respectively to the total gender gap.

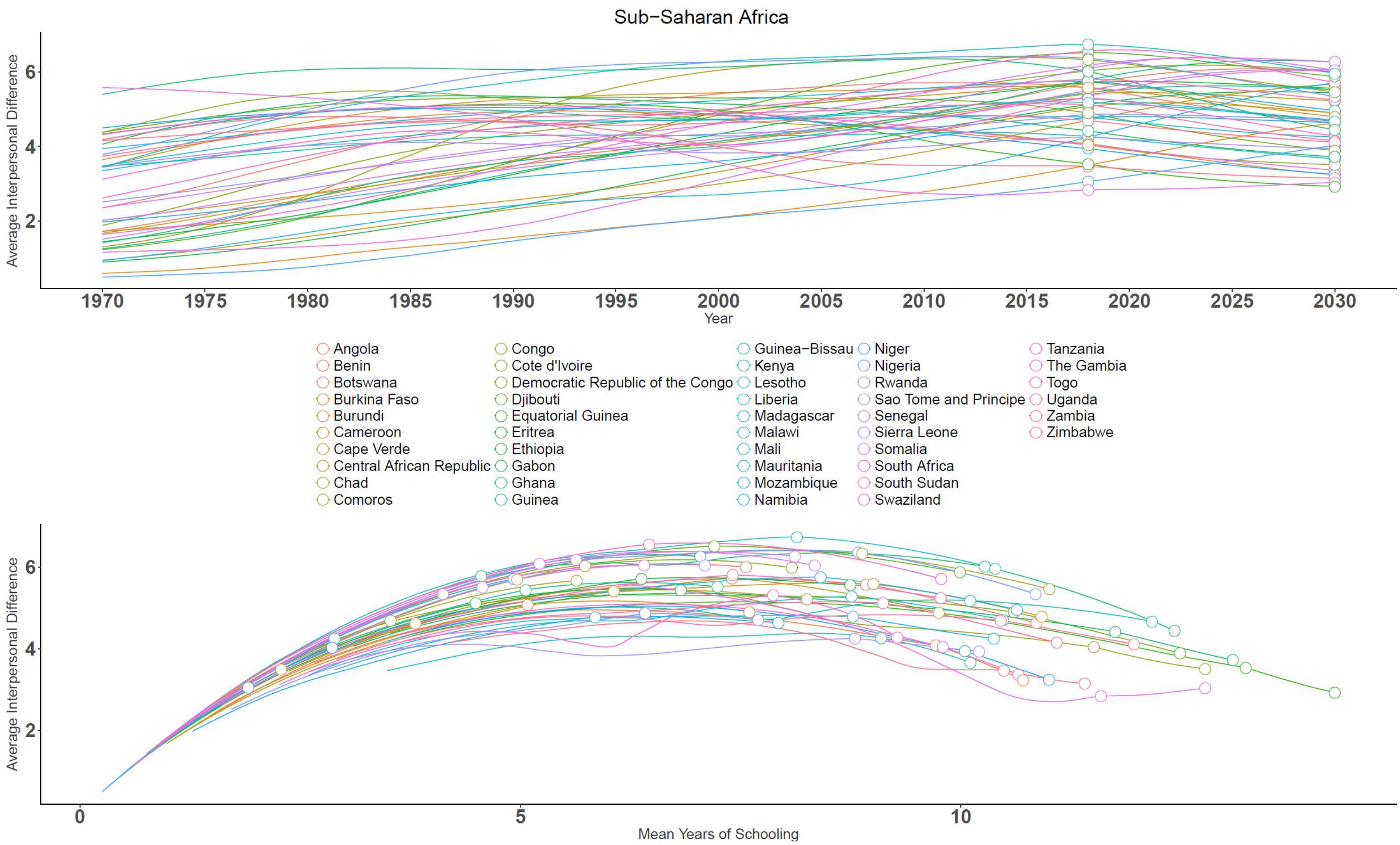
Sub−Saharan Africa
Average Interpersonal Difference
Year
Angola
Benin
Botswana
Burkina Faso
Burundi
Cameroon
Cape Verde
Central African Republic
Chad
Comoros
Congo
Cote d'Ivoire
Democratic Republic of the Congo
Djibouti
Equatorial Guinea
Eritrea
Ethiopia
Gabon
Ghana
Guinea
Guinea−Bissau
Kenya
Lesotho
Liberia
Madagascar
Malawi
Mali
Mauritania
Mozambique
Namibia
Niger
Nigeria
Rwanda
Sao Tome and Principe
Senegal
Sierra Leone
Somalia
South Africa
South Sudan
Swaziland
Tanzania
The Gambia
Togo
Uganda
Zambia
Zimbabwe
Average Interpersonal Difference
Mean Years of Schooling

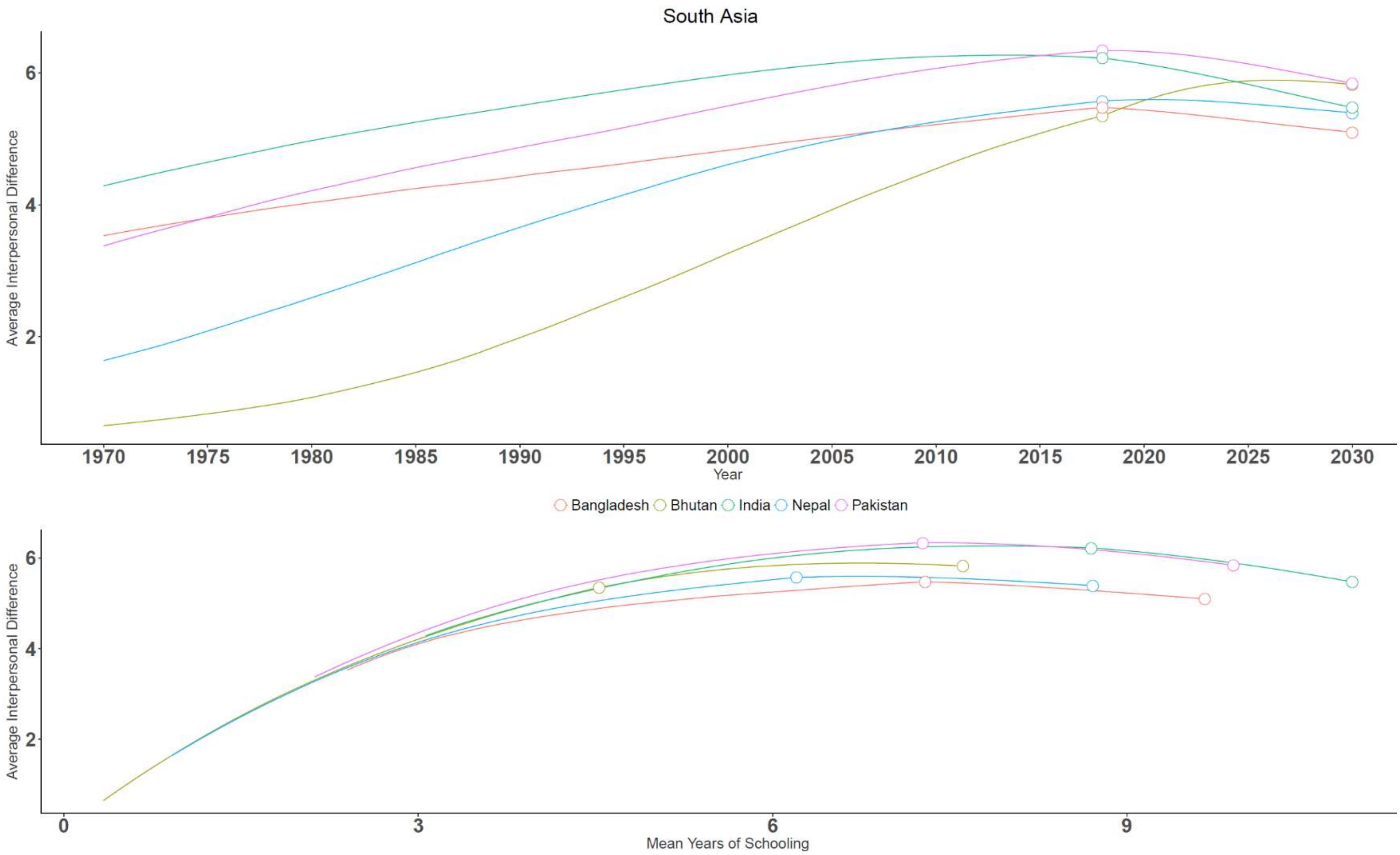
South Asia
Average Interpersonal Difference
Year
Bangladesh
Bhutan
India
Nepal
Pakistan
Average Interpersonal Difference
Mean Years of Schooling

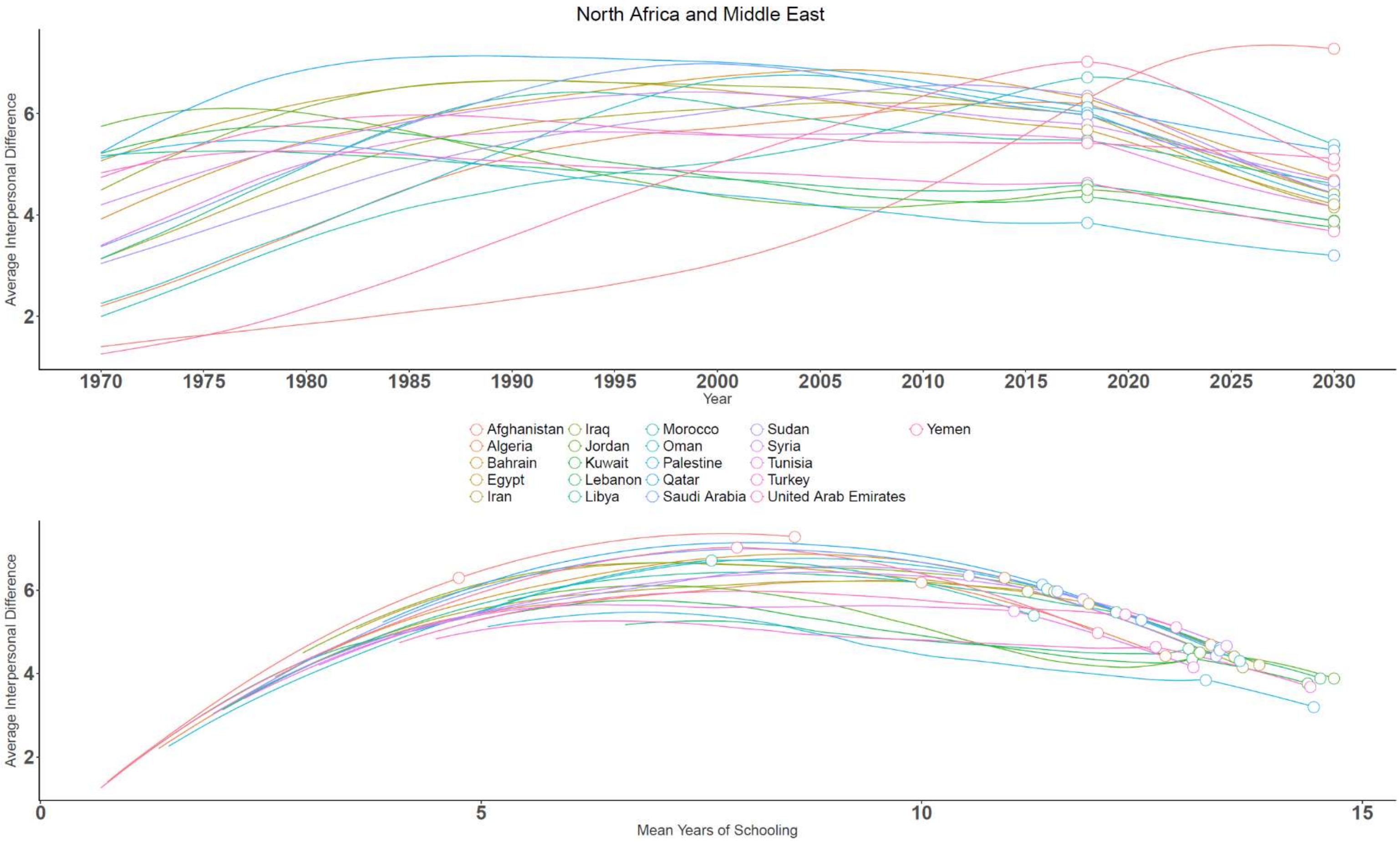
North Africa and Middle East
Average Interpersonal Difference
Year
1970
1975
1980
1985
1990
1995
2000
2005
2010
2015
2020
2025
2030
Afghanistan
Algeria
Bahrain
Egypt
Iran
Iraq
Jordan
Kuwait
Lebanon
Libya
Morocco
Oman
Palestine
Qatar
Saudi Arabia
Sudan
Syria
Tunisia
Turkey
United Arab Emirates
Yemen
Mean Years of Schooling

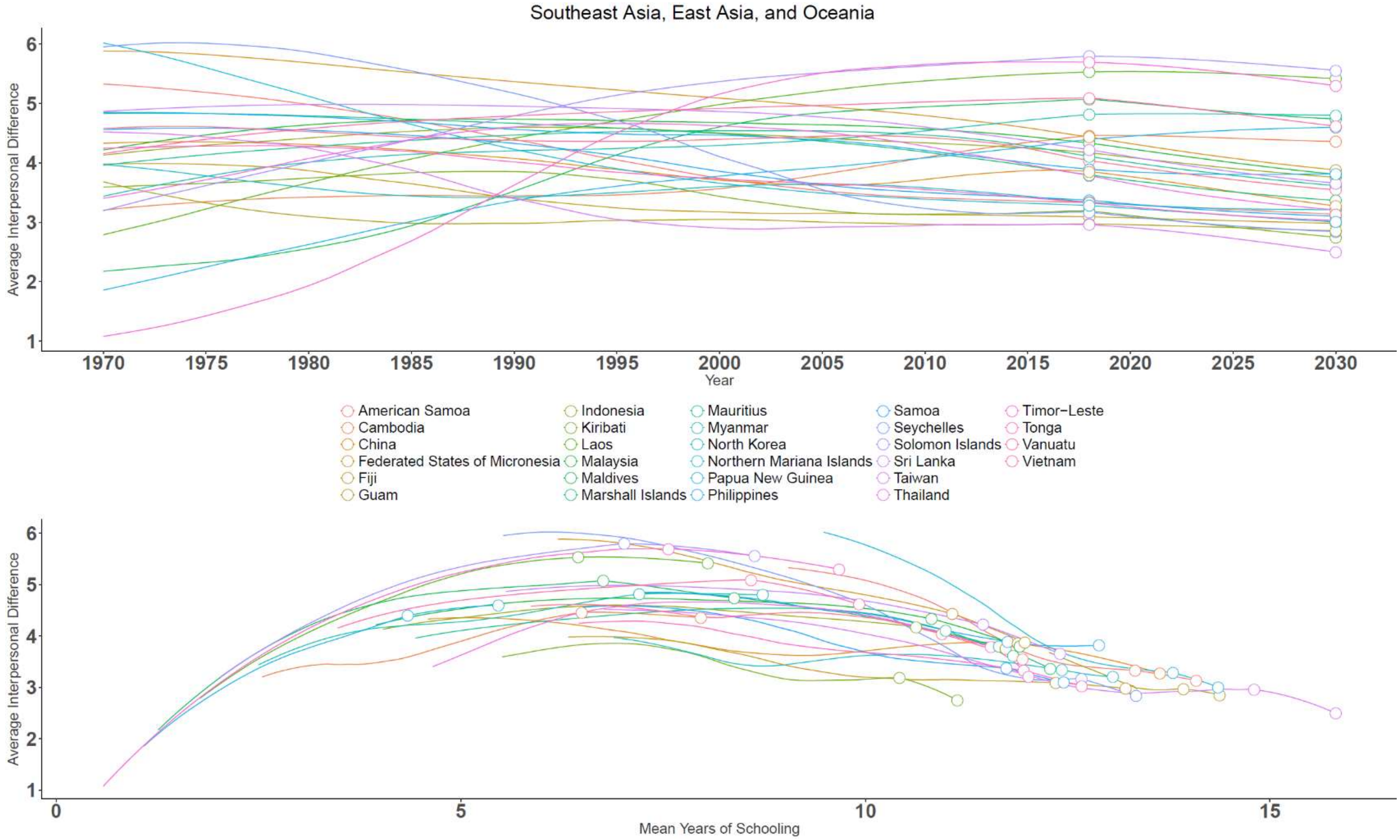
Southeast Asia, East Asia, and Oceania
Average Interpersonal Difference
Year
American Samoa
Cambodia
China
Federated States of Micronesia
Fiji
Guam
Indonesia
Kiribati
Laos
Malaysia
Maldives
Marshall Islands
Mauritius
Myanmar
North Korea
Northern Mariana Islands
Papua New Guinea
Philippines
Samoa
Seychelles
Solomon Islands
Sri Lanka
Taiwan
Thailand
Timor−Leste
Tonga
Vanuatu
Vietnam
Mean Years of Schooling

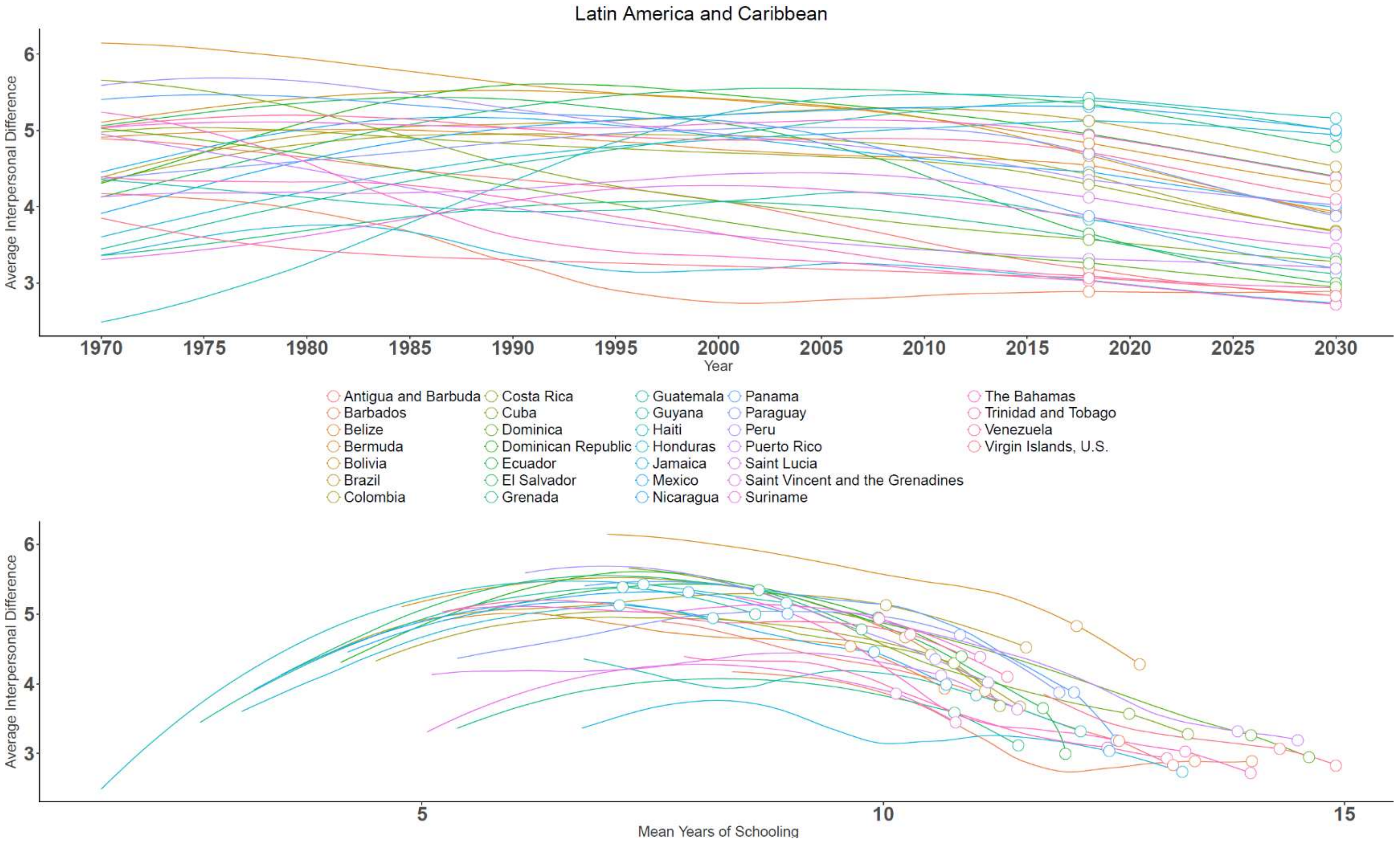
Latin America and Caribbean
Average Interpersonal Difference
Year
Mean Years of Schooling
Antigua and Barbuda
Barbados
Belize
Bermuda
Bolivia
Brazil
Colombia
Costa Rica
Cuba
Dominica
Dominican Republic
Ecuador
El Salvador
Grenada
Guatemala
Guyana
Haiti
Honduras
Jamaica
Mexico
Nicaragua
Panama
Paraguay
Peru
Puerto Rico
Saint Lucia
Saint Vincent and the Grenadines
Suriname
The Bahamas
Trinidad and Tobago
Venezuela
Virgin Islands, U.S.

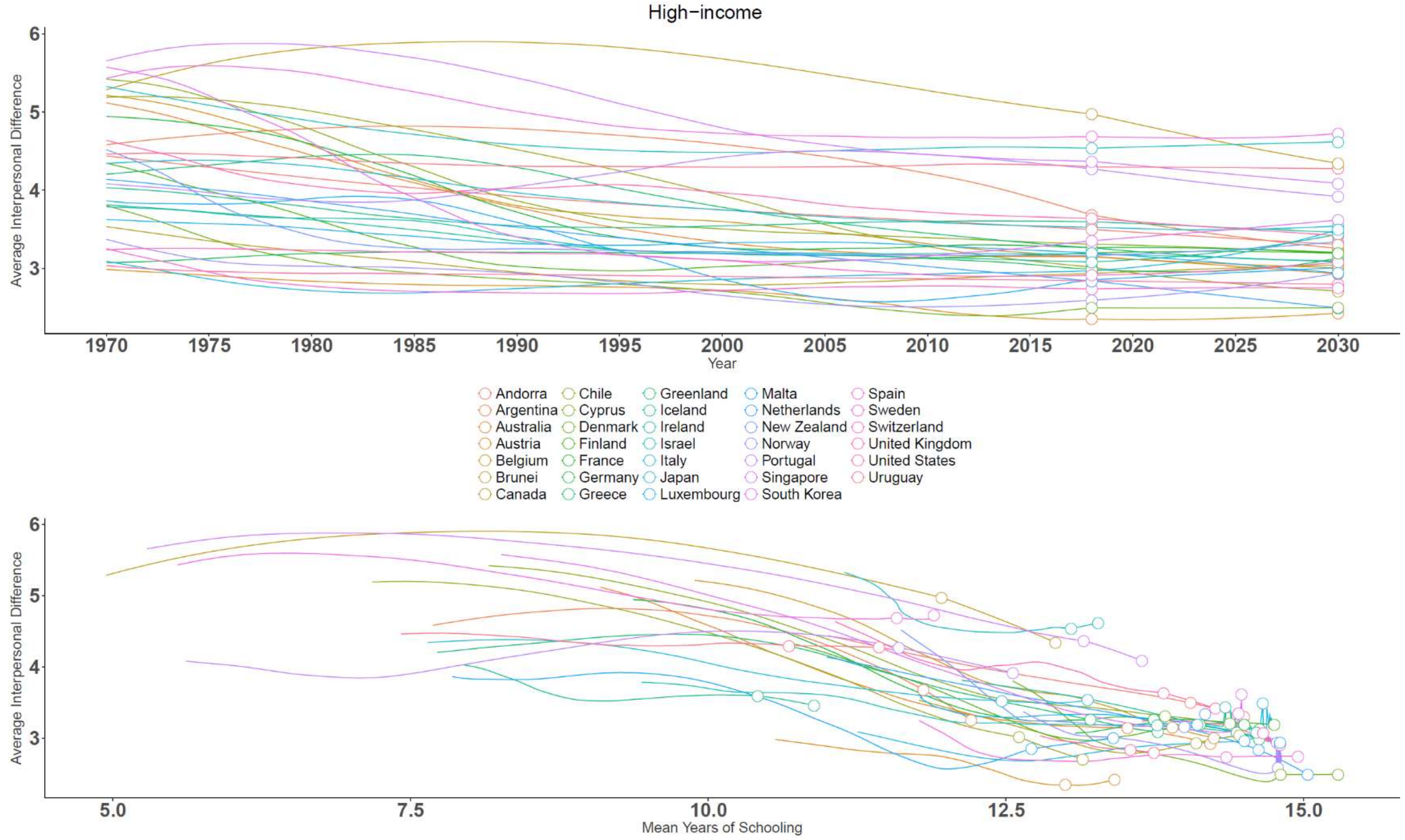
High-income
Average Interpersonal Difference
Year
Mean Years of Schooling
Andorra
Argentina
Australia
Austria
Belgium
Brunei
Canada
Chile
Cyprus
Denmark
Finland
France
Germany
Greece
Greenland
Iceland
Ireland
Israel
Italy
Japan
Luxembourg
Malta
Netherlands
New Zealand
Norway
Portugal
Singapore
South Korea
Spain
Sweden
Switzerland
United Kingdom
United States
Uruguay

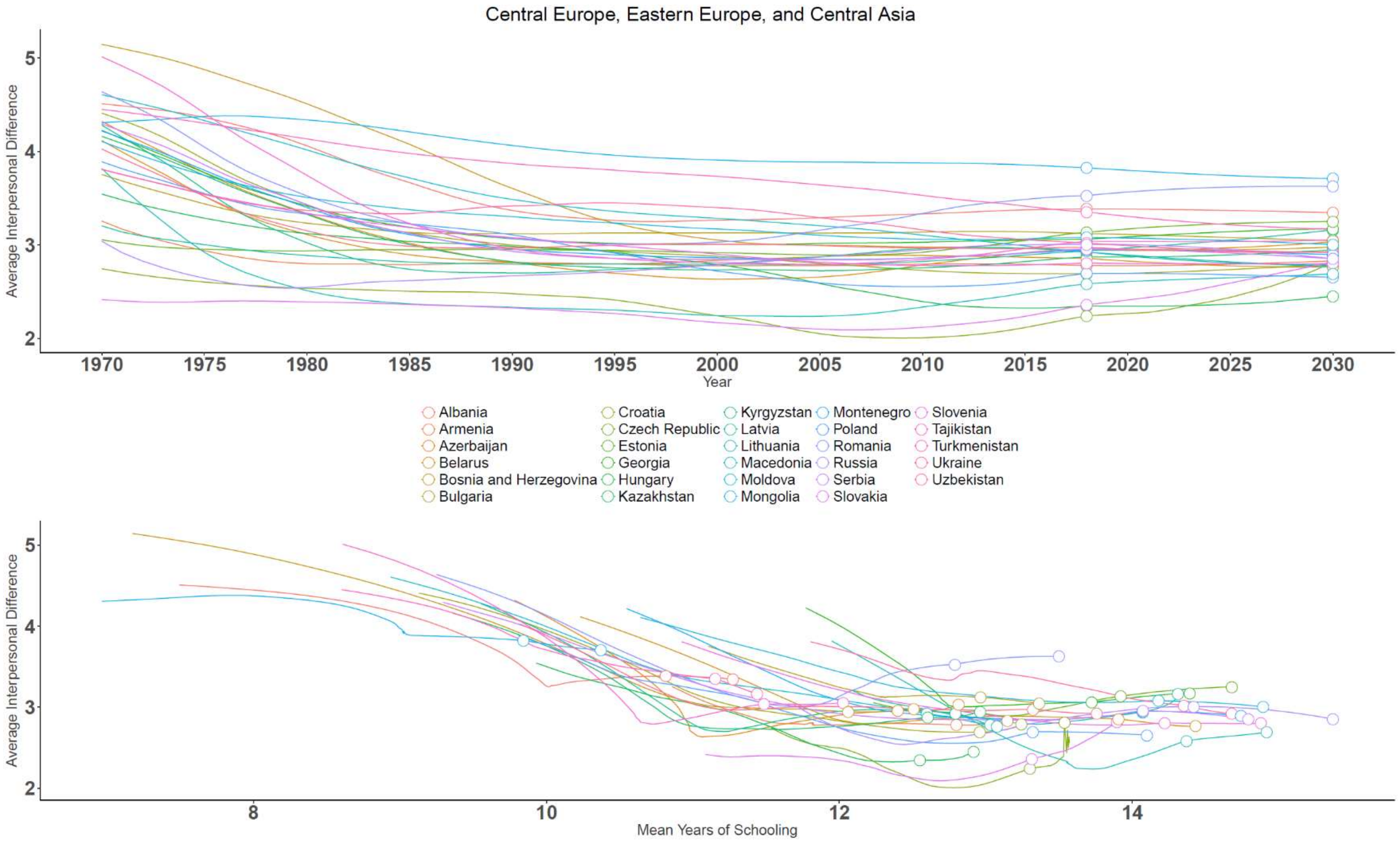

Supplemental Figure 15. Country Specific Kuznets Curves

Educational inequality, represented by the AID, is represented on the y axis against time (top) and the mean years of schooling (bottom) by country, separate for each super-region. The dots mark 2018, the beginning of forecasts, and 2030, the SDG target year.
