# Supplementary material for: Measuring and forecasting progress towards the education-related SDG targets
Source: Nature. 2020 Apr 15;580(7805):636–9. doi: 10.1038/s41586-020-2198-8 (PMC7332421; doi:10.1038/s41586-020-2198-8)
Supplement: Supplementary file 2 — Reporting Summary [file 41586_2020_2198_MOESM2_ESM.pdf]

nature research

Corresponding author(s): Emmanuela Gakidou

Last updated by author(s): Feb 4, 2020

Reporting Summary

Nature Research wishes to improve the reproducibility of the work that we publish. This form provides structure for consistency and transparency in reporting. For further information on Nature Research policies, see Authors & Referees and the Editorial Policy Checklist.

Statistics

For all statistical analyses, confirm that the following items are present in the figure legend, table legend, main text, or Methods section.

n/a	Confirmed	
☐	☒	The exact sample size (n) for each experimental group/condition, given as a discrete number and unit of measurement
☐	☒	A statement on whether measurements were taken from distinct samples or whether the same sample was measured repeatedly
☒	☐	The statistical test(s) used AND whether they are one- or two-sided<br>*Only common tests should be described solely by name; describe more complex techniques in the Methods section.*
☒	☐	A description of all covariates tested
☐	☒	A description of any assumptions or corrections, such as tests of normality and adjustment for multiple comparisons
☐	☒	A full description of the statistical parameters including central tendency (e.g. means) or other basic estimates (e.g. regression coefficient) AND variation (e.g. standard deviation) or associated estimates of uncertainty (e.g. confidence intervals)
☐	☒	For null hypothesis testing, the test statistic (e.g. F, t, r) with confidence intervals, effect sizes, degrees of freedom and P value noted<br>*Give P values as exact values whenever suitable.*
☐	☒	For Bayesian analysis, information on the choice of priors and Markov chain Monte Carlo settings
☒	☐	For hierarchical and complex designs, identification of the appropriate level for tests and full reporting of outcomes
☒	☐	Estimates of effect sizes (e.g. Cohen's d, Pearson's r), indicating how they were calculated

Our web collection on statistics for biologists contains articles on many of the points above.

Software and code

Policy information about availability of computer code

Data collection	No primary data collection was carried out for this analysis.
Data analysis	All analyses were conducted using R version 3.1.3 and Python 2.7.3.

For manuscripts utilizing custom algorithms or software that are central to the research but not yet described in published literature, software must be made available to editors/reviewers. We strongly encourage code deposition in a community repository (e.g. GitHub). See the Nature Research guidelines for submitting code & software for further information.

Data

Policy information about availability of data

All manuscripts must include a data availability statement. This statement should provide the following information, where applicable:

- Accession codes, unique identifiers, or web links for publicly available datasets
- A list of figures that have associated raw data
- A description of any restrictions on data availability

This study used data that are available from public online repositories, but which in most cases require a straightforward registration process and usage agreement with the data provider. A detailed table of data sources and availability can be found in the supplement. Although the authors are restricted from providing the data directly in most cases, specific data sets may be made available by request and with permission from the data provider. The authors may be contacted for assistance in acquiring data for the replication of this study.

Field-specific reporting

Please select the one below that is the best fit for your research. If you are not sure, read the appropriate sections before making your selection.

☐ Life sciences ☒ Behavioural & social sciences ☐ Ecological, evolutionary & environmental sciences

For a reference copy of the document with all sections, see nature.com/documents/nr-reporting-summary-flat.pdf

Behavioural & social sciences study design

All studies must disclose on these points even when the disclosure is negative.

Study description	A descriptive, population-level, ecological study of inequality
Research sample	All available nationally representative census and survey data containing information about educational attainment.
Sampling strategy	We used all available nationally representative census and survey data containing information about educational attainment. .
Data collection	N/A - secondary analysis
Timing	N/A - Secondary analysis, data originally collected between 1950 and 2018 were included.
Data exclusions	As described in the methods section, with greater detail in the supplement, this study provides modeled estimates of the single-year distribution of years of schooling over time and by country. Data were included from 195 nations and territories that are part of the Global Burden of Disease 2017 study. Data for other areas that do not pertain to this list, or which were found to not be nationally representative, were not included. Data that did not include 5-year age groups, or which were not disaggregated by age or sex were also not included. Data from outside the 1950-2018 time period were also not included.
Non-participation	N/A - secondary analysis of nationally-representative statistics
Randomization	N/A - observational analysis, no experimental groups

Reporting for specific materials, systems and methods

We require information from authors about some types of materials, experimental systems and methods used in many studies. Here, indicate whether each material, system or method listed is relevant to your study. If you are not sure if a list item applies to your research, read the appropriate section before selecting a response.

Materials & experimental systems

n/a	Involved in the study	
☒	☐	Antibodies
☒	☐	Eukaryotic cell lines
☒	☐	Palaeontology
☒	☐	Animals and other organisms
☒	☐	Human research participants
☒	☐	Clinical data

Methods

n/a	Involved in the study	
☒	☐	ChIP-seq
☒	☐	Flow cytometry
☒	☐	MRI-based neuroimaging
